# In situ azetidine polymerization elevates the performance of commercial polymer resin sorbents in carbon dioxide capture

**DOI:** 10.1126/sciadv.aeg5572

**Published:** 2026-09-30

**Authors:** Arkaprabha Giri, UnJin Ryu, Jiaqi Zhang, Opeyemi Ojelade, Wenyang Zhao, Jacob Hoffman, Mark Robertson, Jordi Espín, Madison Nichols, Surya Parker, Omid Ghaffari Nik, Christopher W. Jones

**Affiliations:** ^1^ School of Chemical & Biomolecular Engineering, Georgia Institute of Technology, Atlanta, GA 30332, United States.; ^2^ CarbonCapture Inc., 1242 Palmetto St., Los Angeles, CA 90013, United States.

## Abstract

Commercial, amine-containing porous polymer resins are popular sorbents for CO_2_ removal from air, otherwise known as direct air capture (DAC). However, their limited amine density and poor oxidative stability constrain performance. Herein, we introduce a simple one-step method to add additional amine groups onto a commercial resin, such as Lewatit VP OC 1065, by grafting branched poly(propyleneimine) oligomers via in situ acid-catalyzed ring-opening polymerization of azetidine. This “grafting-from” modification strategy enhances the CO_2_ capacity by >1.4-fold compared to pristine resin, reaching 1.54 and 3.34 mmol⋅g^−1^ under dry DAC (400 ppm CO_2_, 30°C) and flue-gas (15% CO_2_, 40°C) conditions, respectively. Under humid DAC conditions, the capacity reaches ∼2.5 mmol⋅g^−1^ at 25°C and further increases to ∼3.0 mmol⋅g^−1^ at −5°C. The optimal sample shows enhanced oxidation resistance under air and maintains improved cycling stability over 150 adsorption-desorption cycles under simulated flue-gas conditions. Overall, this work demonstrates a scalable, versatile strategy to upgrade commercial materials into more efficient and durable sorbents for carbon capture.

## INTRODUCTION

To limit global warming to <2°C, ∼10 Gt CO_2_ must be removed from the atmosphere annually by 2050, growing to 20 Gt CO_2_ per year by 2100 (*1*–*4*). Among the available negative emission technologies (NETs), direct air capture (DAC) has emerged as an attractive option due to its potential scalability, offering modest land-use requirements, adaptability in plant site selection, and access to extensive geological reservoirs for long-term CO_2_ storage (*5*–*8*). Extensive research efforts have focused on the development of sorbents for DAC (*9*–*17*). Based on the current performance of commercially available sorbents, DAC costs are projected to be high for early deployments, with cost reductions expected via material and process optimization and economies of scale (*18*–*20*). Achieving economically viable DAC demands major improvements in solid sorbent design and performance (*21*). The U.S. National Academies have highlighted the need for next-generation sorbents with higher adsorption capacity, faster diffusion kinetics, improved cycle stability, low-cost and scalable materials, and reduced sensible heat loads from inert supports (*22*). The U.S. Department of Energy (DOE) has set a target of atmospheric CO_2_ capture and storage at a cost of less than $100 per net metric ton, emphasizing the urgent need for innovation in sorbent-based DAC systems (*23*).

In this context, self-supported polymeric amine materials hold great promise as highly efficient DAC sorbents due to (i) their resistance to amine leaching during regeneration (compared to physically impregnated amine sorbents), (ii) reduced sensible heat loads owing to the absence of inert supports, and (iii) low-cost, scalable fabrication compared with many benchmark amine-impregnated or grafted solids such as MOFs, COFs, mesoporous silicas (e.g., SBA-15, MCM-41), and porous carbons (*24*–*31*). Recent years have witnessed growing interest in the development of such polymeric sorbents demonstrating promising DAC performance (*32*–*37*). In particular, primary amine-functionalized poly(styrene-divinylbenzene) (PS-DVB) resins are especially attractive owing to their high thermal and hydrothermal stability, permanent porosity, hydrophobic polymer backbone, and cost-effective scalable synthesis (*38*). Among them, Lewatit VP OC 1065 (LWT), a commercially available meso−/macroporous PS-DVB resin functionalized with primary amines, has emerged as a benchmark sorbent in DAC studies (Fig. 1A) (*39*–*42*). However, oxidative degradation remains a bottleneck for this class of resins for long term use (*43*, *44*).

**Fig. 1.**
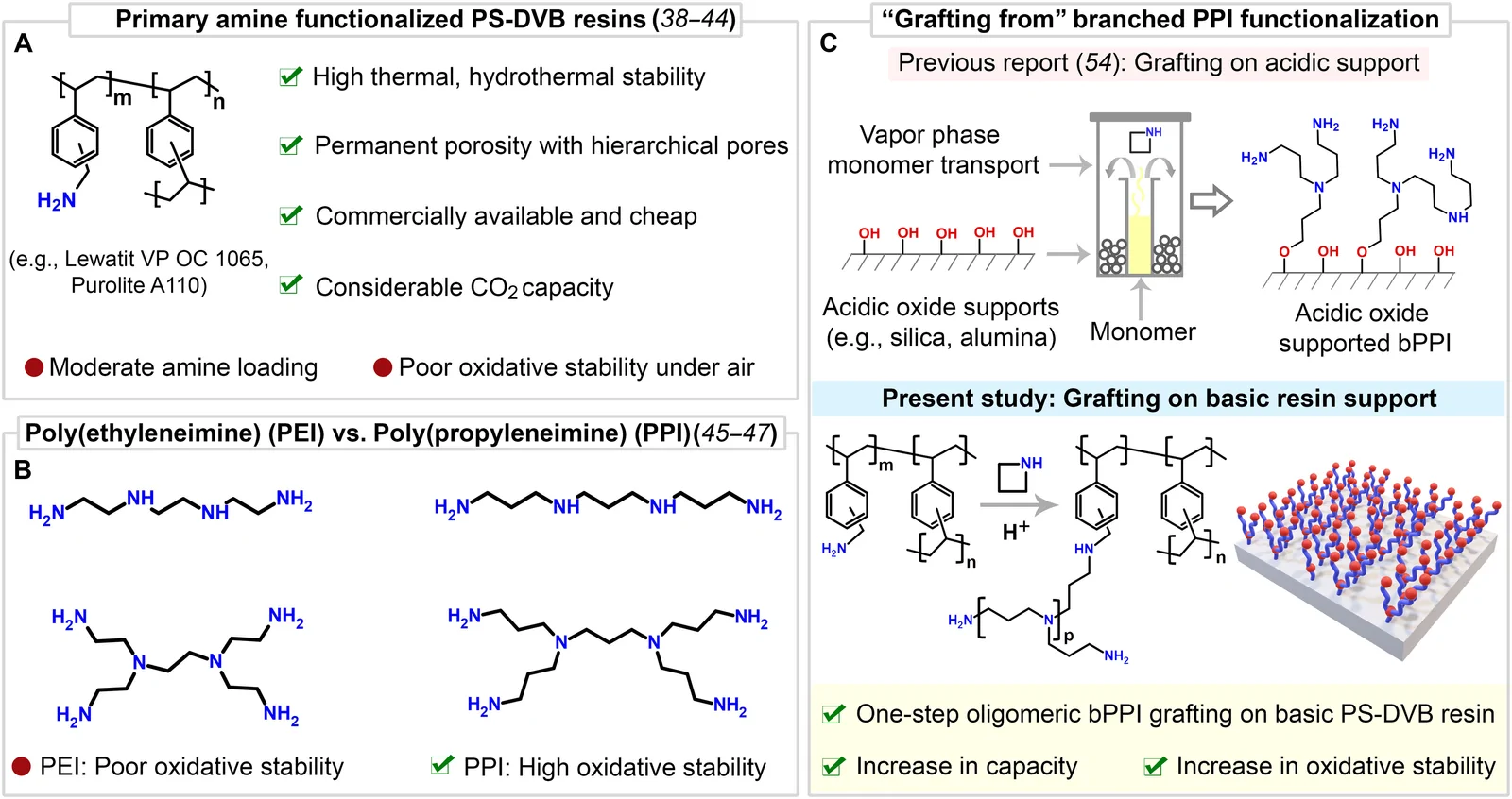
“Grafting from” strategy for the fabrication of branched poly(propyleneimine)-functionalized poly(styrene-divinylbenzene) (PS-DVB) resin sorbents for CO_2_ capture. (**A**) Advantages and limitations of commercial primary amine-functionalized PS-DVB resin sorbents for CO_2_ capture. (**B**) Structures of different poly(ethyleneimine) (PEI) and poly(propyleneimine) (PPI) model architectures. (**C**) Comparison between previously reported branched PPI grafting on acidic oxide supports via vapor-phase monomer transport and the present “grafting-from” strategy for branched PPI functionalization of basic PS-DVB resin supports through solvothermal acid-catalyzed in situ ring-opening polymerization of azetidine (*38*–*47*, *54*).

In our previous work, we found that poly(propyleneimine) (PPI) exhibits superior oxidative stability, lower hydrophilicity, and higher amine efficiency compared to the benchmark poly(ethyleneimine) (PEI) (Fig. 1B) (*45*–*47*). Building on these findings, we hypothesized that combining the advantageous properties of PPI with amine-functionalized porous PS-DVB resins could provide a hybrid sorbent platform with higher amine loadings, enhanced CO_2_ capacity, and improved oxidative stability. Herein, we developed a facile methodology to increase the amine content of primary amine-functionalized PS-DVB resins, specifically Lewatit VP OC 1065 (LWT), by chemically grafting branched poly(propyleneimine) (bPPI) oligomer onto the resins through a one-step metal-free modification method, i.e., in situ Brønsted acid-catalyzed ring-opening polymerization of azetidine (Fig. 1C). This “grafting-from” technique offers several advantages over post-synthetic attachment of preformed poly(amines) onto porous supports (Fig. 2, fig. S1) (*16*, *19*, *48*). By facilitating controlled polymer growth directly from the surface, it prevents crosslinking-induced loss of anchoring sites and primary/secondary amine functionalities, while promoting uniform amine distribution, minimized pore blockage, and high amine loading. In addition, azetidine and it’s salts are commercially available, inexpensive (e.g., azetidine hydrochloride, ≈ $2–5 kg^−1^), exhibit greater stability, and have lower volatility compared to other heterocyclic amine precursors, such as aziridine, making the overall grafting process more user-friendly.

**Fig. 2.**
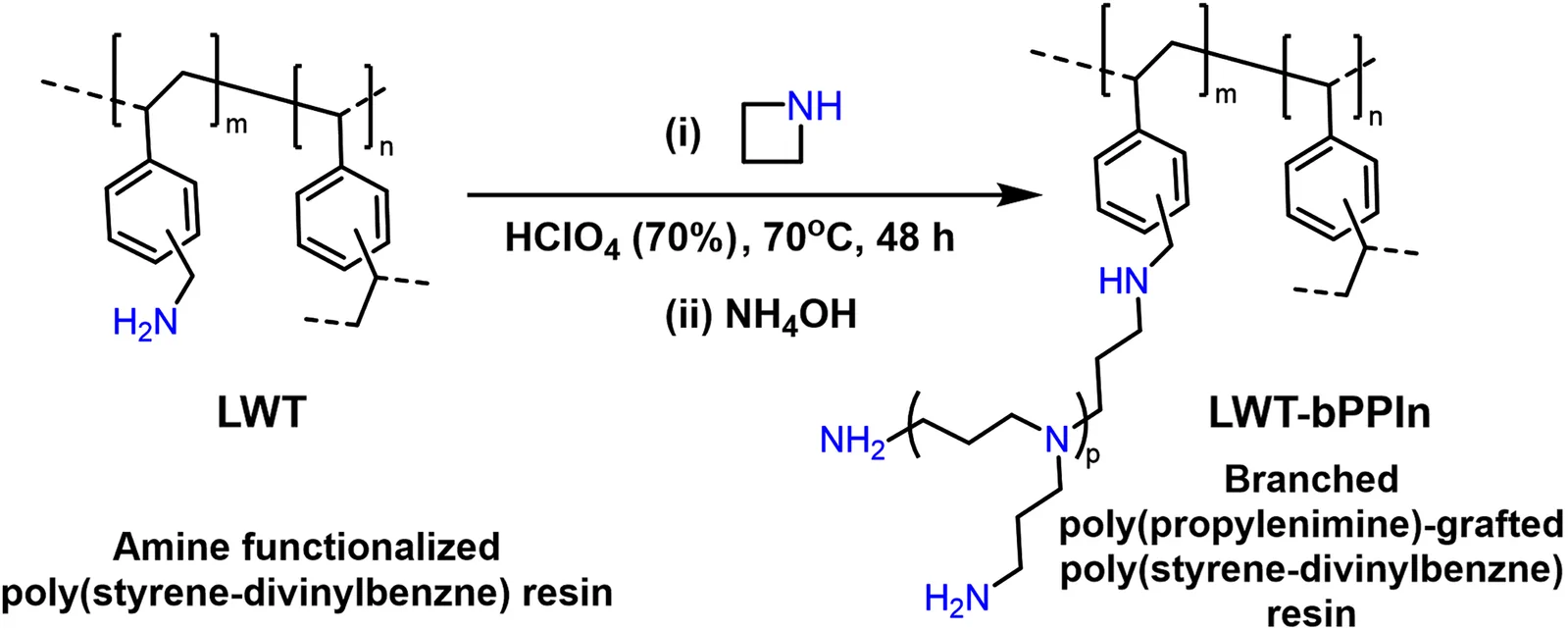
Schematic illustration of branched poly(propyleneimine) (bPPI) oligomer grafting onto a commercial primary amine-containing resin through acid-catalyzed ring-opening polymerization of azetidine.

We evaluated both the pristine (LWT) and bPPI oligomer-grafted resins (LWT-bPPIn, n = 1–9) for CO_2_ sorption from simulated air (400 ppm CO_2_/N_2_) under ambient and sub-ambient temperatures (−20 to 25°C) across a wide range of relative humidities (0%–90%) to mimic realistic weather conditions for widespread DAC deployment. Under dry DAC conditions, the bPPI grafted resins exhibited a >1.4-fold enhancement in CO_2_ uptake compared to the pristine resin. Breakthrough measurements under humid conditions (70% RH) further revealed a pseudo-equilibrium capacity of 2.5 mmol g^−1^ at 25°C, which increased to 3.1 mmol g^−1^ at −5°C for the optimal LWT-bPPI5, showing up to a 36% improvement in the capacity compared to LWT. Furthermore, accelerated aging tests under humid air at elevated temperature demonstrated that bPPI grafting significantly enhances the oxidative stability of the polymer resin. LWT-bPPI5 showed excellent cycling stability both under ambient/sub-ambient DAC conditions with mild laboratory regeneration requirements (80°C). Finally, under standard flue-gas conditions (15% CO_2_, 40°C), LWT-bPPI5 achieved a 1.6-fold higher CO_2_ uptake with superior cycling performance over 150 cycles compared to pristine LWT. Therefore, this study introduces a simple, scalable synthetic route for grafting bPPI oligomer on commercial, primary amine containing PS-DVB resin supports, yielding highly stable and recyclable sorbents for DAC and flue-gas capture across a wide range of temperatures and relative humidities.

## RESULTS

### Synthesis and characterization of branched poly(propyleneimine) oligomer-grafted porous polymeric resin

In previous studies, the cationic ring-opening polymerization of azetidine to afford randomly branched poly(propyleneimine) (PPI) was systematically investigated using various acid catalysts, and the underlying polymerization mechanism has been elucidated by different groups (*49*–*53*). Among the various Brønsted acids examined, we observed that HClO_4_ was the most effective catalyst, affording higher degrees of polymerization in solution within a relatively shorter time (*53*). In addition, we previously developed a unique methodology for the chemical grafting of bPEI and bPPI on acidic oxide supports (such as silica and alumina) through vapor-phase transport of small nitrogen containing heterocyclic monomers including aziridine and azetidine, followed by surface initiated polymerization (Fig. 1C) (*54*, *55*). However, this vapor-phase approach is not directly applicable to the present system because the support, Lewatit VP OC 1065 (LWT), contains weakly basic primary amine functionalities rather than acidic surface sites. Therefore, we hypothesized that bPPI oligomers could be chemically grafted onto LWT via in situ polymerization of azetidine under solvothermal conditions by using an optimum amount of acid catalyst (Figs. 2 and 3A). To explore this, we optimized the reaction by systematically tuning the amount of HClO_4_ catalyst (0.25–3.7 equivalents w.r.t. azetidine) and varying the azetidine loading (7–20 equivalents w.r.t. the N content of LWT resin) (Table 1) (fig. S2). Since pristine LWT resins consist of beads with varying particle sizes (300 μm to +850 μm) exhibiting varying CO_2_ uptake capacities, the beads were ground prior to functionalization to ensure a homogeneous and reproducible grafting process (*42*). Accordingly, all optimization reactions were performed using powdered LWT samples obtained by grinding the resin beads and sieving the resulting powder through a 300 μm mesh. The obtained bPPI oligomer-grafted resins (LWT-bPPIn, n = 1–9) (Table 1) were thoroughly washed with copious amounts of aqueous ammonium hydroxide and methanol to remove acidic impurities and surface-adsorbed bPPI. The samples were then dried overnight under vacuum. We further performed a scale-up synthesis of the LWT-bPPI5 (6-fold) while maintaining the optimized reaction conditions (Table 1, entry 6), demonstrating the potential scalability of this methodology (see detailed procedure, Materials and methods).

**Fig. 3.**
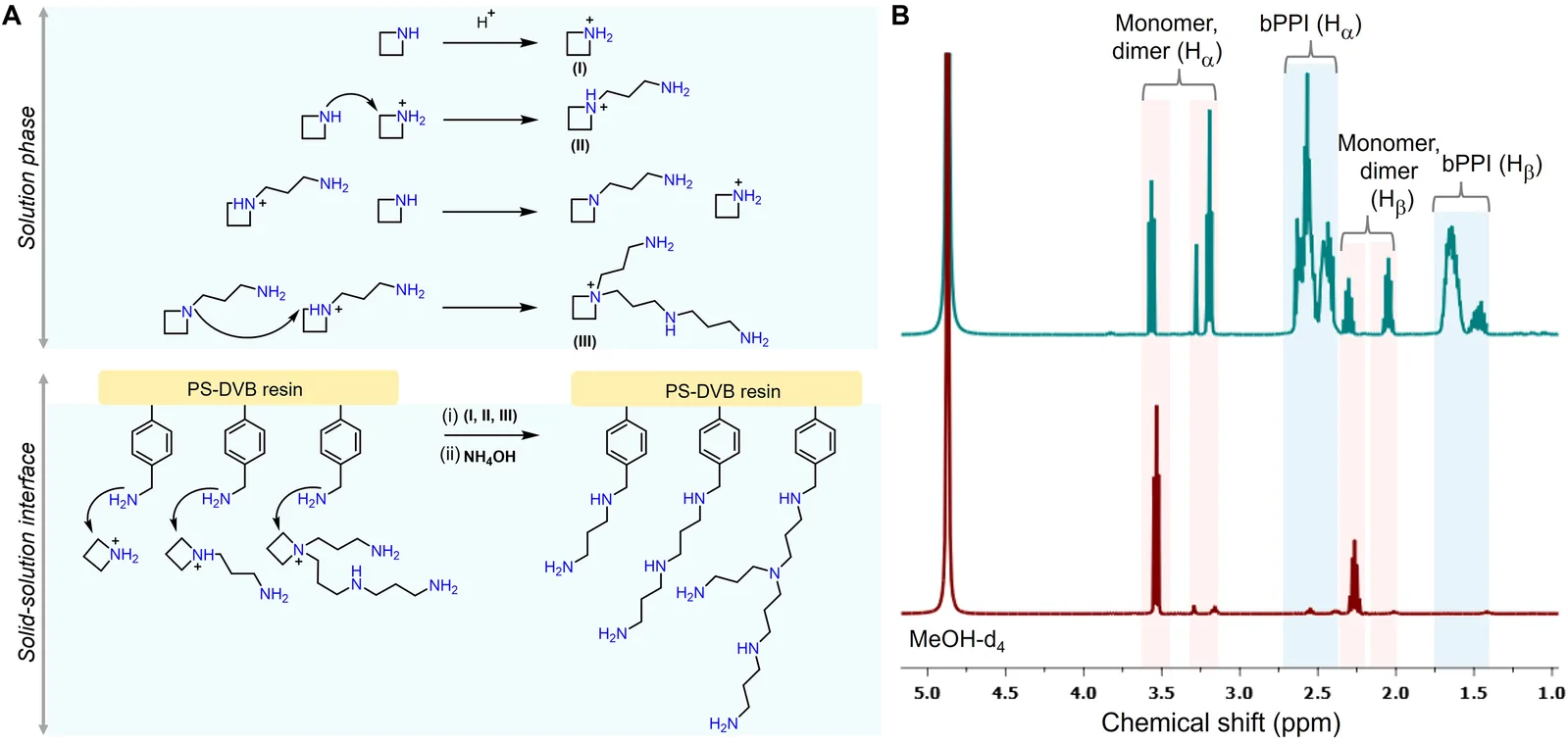
Proposed mechanism for in situ azetidine polymerization and bPPI grafting onto amine-functionalized PS-DVB resin. (**A**) Plausible mechanism for the chemical grafting of branched poly(propyleneimine) (bPPI) on amine-functionalized poly(styrene-divinylbenzene) (PS-DVB) resin through in situ acid-catalyzed ring opening polymerization of azetidine. The mechanism has been adopted from ref. *51*. (**B**) Stacked ^1^H NMR spectra (in MeOH-D_4_) of the reaction filtrates obtained using 0.5 equiv. of HClO_4_ with respect to azetidine (top) and 3.7 equiv. of HClO_4_ (bottom).

**Table 1.** Optimization for grafting branched poly(propyleneimine). Optimization of the reaction conditions for the synthesis of branched poly(propyleneimine) (bPPI)-grafted polymeric resin via in situ acid-catalyzed cationic ring-opening polymerization of azetidine. Optimization of reaction conditions: LWT (grounded in powder and sieved through 300 μm mesh, 50 mg), anhydrous methanol (2 mL), azetidine (7–20 eq. w.r.t. N content of LWT), HClO_4_ (70%, 0.25–3.7 eq. w.r.t. azetidine), temperature (70°C), reaction time (48 h).

Entry	Resin	Azetidine (eq. w.r.t. −NH_2_ in LWT)	HClO_4_ (eq. w.r.t. azetidine)	Specific N content (mmol g^−1^)^#^
1	LWT	–	–	5.7 ± 0.2
2	LWT-bPPI1	7	3.7	5.8
3	LWT-bPPI2	7	2	5.9
4	LWT-bPPI3	7	1	6.0
5	LWT-bPPI4	7	0.86	7.1
6	LWT-bPPI5	7	0.5	8.7 ± 0.1
7	LWT-bPPI6	7	0.25	7.7
8	LWT-bPPI7	10	0.5	8.3
9	LWT-bPPI8	15	0.5	8.3
10	LWT-bPPI9	20	0.5	8.1

^#^ Specific N content was estimated from CHN elemental analysis.

The pristine resin (LWT) and bPPI oligomer-grafted resins (e.g., LWT-bPPIn) were comparatively characterized using various spectroscopic and microscopic techniques to investigate the structural, functional, and textural changes occurring during the in situ polymerization of azetidine on the amine-functionalized porous resin surface. The N content of the resins before and after bPPI functionalization was determined by CHN elemental analysis (Table 1 and table S1). The specific N content of pristine LWT resin was found to be 5.7 ± 0.2 mmol g^−1^. Upon chemical grafting of bPPI, the specific N content of the resulting LWT-bPPIn resins increased to as high as 8.7 ± 0.1 mmol g^−1^ (Table 1, Entry 6–10). Interestingly, when the HClO_4_ catalyst concentration exceeded 1 eq. relative to azetidine, minimal increase in specific N content was observed in the final LWT-bPPIn resins (LWT-bPPI1 to LWT-bPPI3) (fig. S2). To understand the effect of acid concentration on azetidine polymerization, we analyzed the ^1^H NMR spectra of the filtrates obtained after the grafting reactions performed using 3.7 and 0.5 equivalent of HClO_4_ with respect to azetidine (Fig. 3B, Table 1, entry 1, 6) (fig. S3A). At a higher acid loading (3.7 equiv. HClO_4_), the spectrum corresponding to the filtrate of the reaction mixture is dominated by sharp signals corresponding to protonated azetidine monomer, while the characteristic bPPI resonances are nearly absent (Fig. 3B). This observation suggests that excessive acid concentration suppresses polymer growth by protonating the amine functionalities of both the monomer and the surface amine sites on the resin support, thereby significantly reducing their nucleophilicity required for ring-opening polymerization. Consequently, chain propagation becomes kinetically unfavorable, leading predominantly to protonated monomeric or dimeric species, resulting in minimal change in N content of the resin.

In contrast, using <1 eq. of acid catalyst led to a notable increase in N content (LWT-bPPI4 to LWT-bPPI9), with the highest value of 8.7 ± 0.1 mmol/g (for LWT-bPPI5, corresponding to approximately 0–5 repeating units per anchoring site), obtained when 0.5 equivalent of HClO_4_ was used. The ^1^H NMR analysis of filtrate of the reaction mixture also shows broad resonances centered at δ ∼2.6–2.4 and 1.7–1.5 ppm, which are characteristic of the α- and β-methylene protons (H_α_, H_β_) of bPPI, respectively (Fig. 3B) (fig. S3B). The spectral features closely resemble those of pure bPPI, confirming substantial azetidine polymerization under these conditions. Further, the average length of the oligomers formed in the solution phase was estimated from the ^1^H NMR spectrum using Eq. 1 by calculating the degree of polymerization (DP) from the ratio of the integrals of the polymer H resonances to those of the residual azetidine monomer/dimer H resonances (*53*)

$$\mathrm{DP}=\frac{\mathrm{mol}\;H\;\text{polymer}}{\mathrm{mol}\;H\;\text{monomer}/\text{dimer}}$$ (1)

The calculated DP value was ∼6.1, corresponding to an average oligomer length of approximately 6 repeating units. We also qualitatively estimated the degree of branching of the solution-phase bPPI from the ^1^H NMR spectrum by analyzing the α-methylene resonances between 2.4 and 2.7 ppm (fig. S3B). The peak areas were normalized by the corresponding number of α-protons associated with primary (1°), secondary (2°), and tertiary amines (3°) (two, four, and six protons, respectively). The calculated 1°:2°:3° amine ratio was approximately 0.3:0.5:0.2, confirming the formation of branched PPI oligomers (fig. S3C).

Pyrolysis thermogravimetric analysis (TGA) of the starting LWT resin exhibited thermal stability up to 300°C under air (fig. S4). In contrast, the LWT-bPPIn (n = 4–9) resins showed up to ∼15 wt.% mass loss at approximately 180–210°C, indicating the thermal decomposition of the grafted PPI oligomers (*56*). Differential scanning calorimetry (DSC) measurements revealed nearly identical heat flow profiles for LWT and LWT-bPPI5, with a subtle broad thermal feature appearing at approximately 90.2°C in both cases (fig. S5). The similarity between the two profiles confirms that the grafted bPPI exists as short oligomeric chains with limited influence on the bulk polymer matrix dynamics of the parent resin. Structural characterization of unfunctionalized and bPPI-grafted resins (e.g., LWT-bPPI5) was performed using diffuse reflectance infrared Fourier transform spectroscopy (DRIFTS), and ^13^C solid-state cross-polarization magic angle spinning (CP/MAS) NMR spectroscopy. In DRIFTS analysis (Fig. 4A), the variation in the peak patterns in the 3100–3400 cm^−1^ region for LWT and LWT-bPPI5 indicate the presence of primary and secondary amine functionalities. Peaks at 3000–3090 cm^−1^ corresponded to aromatic C-H stretching of the PS-DVB backbone, identical in both resins. Notably, appearance of new peaks and peak broadening were observed in the 2700–2980 cm^−1^ region in LWT-bPPI5 associated with the aliphatic C-H stretching, attributed to the propylenic -CH_2_- groups in the grafted bPPI oligomers. In the 1560–1650 cm^−1^ region, multiple peaks were observed, arising from C=C stretching of the benzene ring in the PS-DVB backbone as well as N-H bending modes. Additionally, a distinct peak at 1122 cm^−1^ was present only in LWT-bPPI5, assignable to C-N stretching vibrations of the bPPI units, suggesting the grafting of bPPI onto the LWT resin (*46*, *57*).

**Fig. 4.**
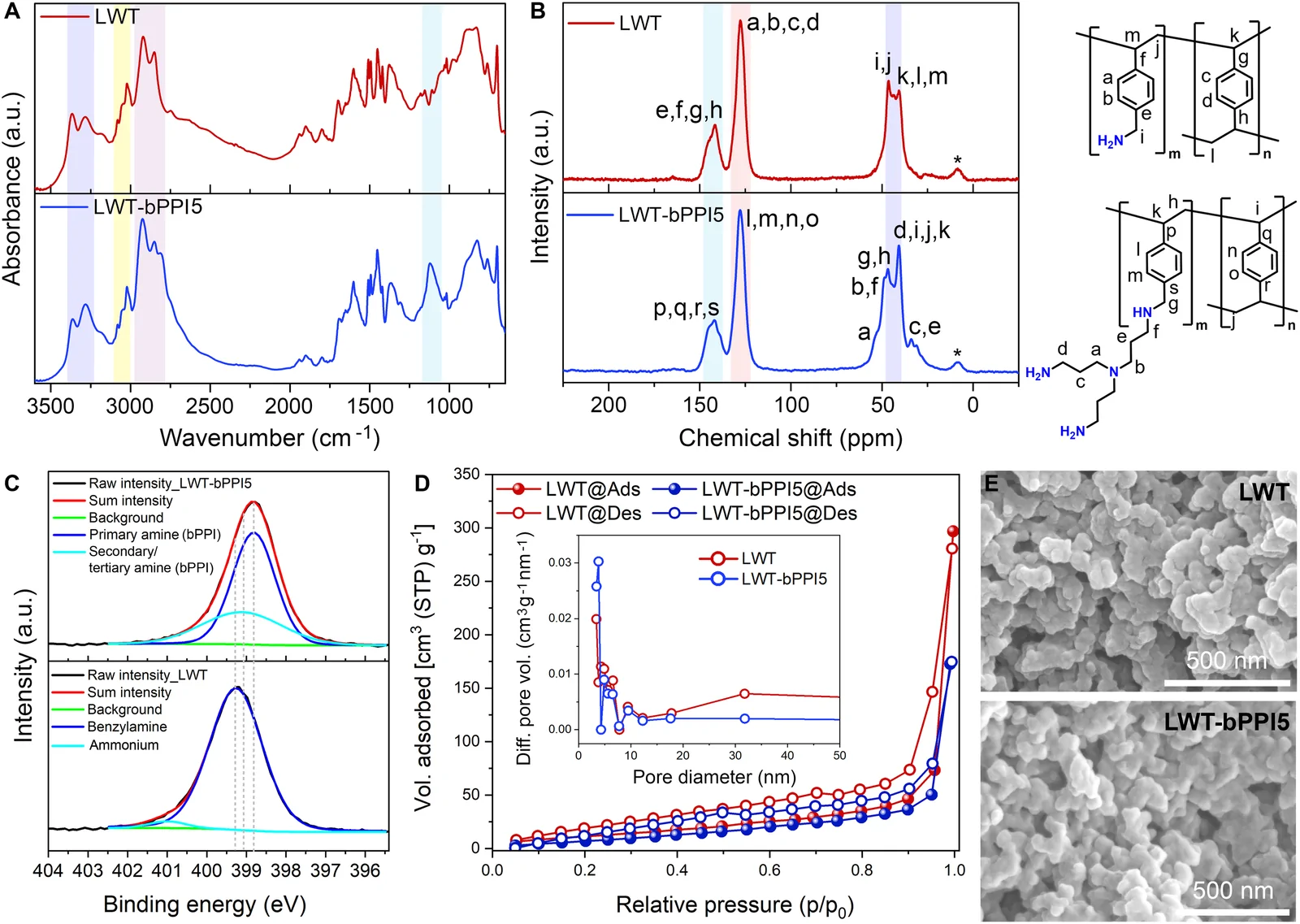
Comprehensive structural and textural characterization of LWT and bPPI-grafted LWT (LWT-bPPI5). (**A**) Diffuse reflectance infrared Fourier transform spectroscopic (DRIFTS) analysis of LWT and LWT-bPPI5. (**B**) Solid-state ^13^C cross-polarization magic angle spinning nuclear magnetic resonance spectroscopic (SS ^13^C CP/MAS NMR) analyses of LWT and LWT-bPPI5. (**C**) N1s XPS spectrum of pristine LWT and LWT-bPPI5. (**D**) N_2_ physisorption isotherms of LWT and LWT-bPPI5 at 77 K. inset: pore size distribution of LWT and LWT-bPPI5 (BJH method). (**E**) Field emission scanning electron microscopic (FESEM) images of LWT (top) and LWT-bPPI5 (bottom). The corresponding images are reused in fig. S7, A and D as enlarged views.

In ^13^C SS CP/MAS NMR, both the LWT and LWT-bPPI5 resins exhibited identical features in the aromatic region (120–150 ppm) (Fig. 4B). The intense peak at 120–135 ppm was attributed to unsubstituted aromatic carbons of the PS-DVB backbone, while peaks at 140–150 ppm corresponded to substituted aromatic carbons of PS-DVB (*44*, *58*). In contrast, notable differences were observed in the aliphatic region (i.e., 25–60 ppm), suggesting the integration of bPPI oligomer into the pristine LWT resin. Newly emerged peaks at 28–34 ppm were assigned to the central carbon of the propylene units in bPPI, whereas peaks in the range of 48–60 ppm were attributed to carbons near N atoms of the bPPI units. Other aliphatic carbons of the PS-DVB backbone appeared at 36–48 ppm. A common peak near ∼12 ppm observed in both resins possibly originating from the aliphatic carbon of the ethylvinylbenzene unit present in the commercial DVB monomer.

In the XPS spectra of pristine LWT, the deconvolution of the N1s peak showed a dominant signal at 399.3 eV, assignable to benzyl amine groups, along with a minor peak at 401.1 eV attributed to ammonium ions (Fig. 4C, fig. S6). After bPPI grafting we observed the overall peak position shift to the lower binding energy region. For LWT-bPPI5, the deconvoluted N 1 s spectrum displayed two peaks at 398.8 and 399.2 eV, attributable to primary and secondary/tertiary amines of the bPPI units, respectively. Notably, the N/C ratios derived from XPS (a surface-sensitive technique) deviated from those obtained via bulk CHN elemental analysis for both pristine LWT and LWT-bPPI5 (N/C ratio of LWT: 0.15 from XPS, 0.09 from CHN elemental analysis; N/C ratio for LWT-bPPI5: 0.24 ± 0.02 from XPS, 0.14 from CHN elemental analysis) (table S2). In primary amine containing weak base anion exchange resins, like LWT, the amine groups are likely introduced via post-synthetic modification of the PS-DVB resin (*33*, *34*). The amine incorporation depends on the resin’s swellability and reagent diffusion, leading to a higher amine density at the surface of the polymer microgel. Consequently, bPPI grafting preferentially occurs at these surface sites.

To evaluate the textural properties of the pristine and bPPI-grafted resins, N_2_ physisorption measurements were conducted at 77 K (Fig. 4D). Both resins exhibited Type IV adsorption isotherms, with a sharp increase in N_2_ uptake at relative pressures (p/p_0_) > 0.9, indicative of capillary condensation within macropores, primarily arising from interparticle voids. A decrease in specific BET surface area (from 48 m^2^ g^−1^ to 37 m^2^ g^−1^) and total pore volume (from 0.46 to 0.27 cm^3^ g^−1^) was observed after bPPI grafting, indicating that the in situ polymerization of azetidine may fill some pores, as further supported by changes in the Barrett–Joyner–Halenda (BJH) pore size distribution (Fig. 4D, inset). FESEM images showed that unfunctionalized LWT (beads are ground into a powder form and sieved through a 300 μm mesh) and LWT-bPPI5 consist of interconnected microgel particles with sizes in the range of 100–150 nm. The FESEM images of LWT-bPPI5 suggest no significant change in the macroscale morphology before and after the bPPI grafting of LWT (Fig. 4E, fig. S7).

To check the nature of pore environment before and after bPPI grafting onto the LWT resin, we have measured water adsorption isotherms at 25°C (fig. S8, table S3). The aromatic-rich PS-DVB backbone renders pristine LWT hydrophobic, resulting in low water uptake (3.3 ± 0.3 mmol g^−1^ at 50% RH, 5.3 ± 0.4 mmol g^−1^ at 70% RH). This may reduce the energy required for regeneration, making the resin particularly attractive for DAC applications. After bPPI-grafting, the hydrophilicity of the resin slightly increases. However, the water uptake capacity of LWT-bPPI5 (5.2 ± 0.6 mmol g^−1^ at 50% RH, 9.0 ± 1.0 mmol g^−1^ at 70% RH) remain lower or comparable to many benchmark poly(amine) functionalized DAC sorbent materials, such as tetraethylenepentamine (TEPA)-impregnated sorbents [g-Al_2_O_3_ (12 mmol g^−1^ at 70% RH), Mg_2_(dobpdc) (14–16 mmol g^−1^), MIL-101(Cr) (24 mmol g^−1^ at 70% RH)] (*59*, *60*), PEI-impregnated Mg_0.55_Al-CO_3_ layered double hydroxides, Mg_0.55_Al-O mixed metal oxides (18–20 mmol g^−1^ at 70% RH) (*61*), and PEI-functionalized COF-999 (5 mmol g^−1^ at 50% RH) (*27*). This may be attributed to the longer three-carbon alkyl spacer between the nitrogen atoms in PPI, which enhances its nonpolar hydrocarbon character relative to amines containing ethylene spacers, such as PEI and TEPA.

### Evaluation of CO_2_ sorption capacity using thermogravimetric analysis

We evaluated the DAC performance of various LWT-bPPIn resins after 6 h of exposure with 400 ppm CO_2_/N_2_ at 30°C under dry conditions using a TGA (Fig. 5A, fig. S9). Among the tested materials, LWT-bPPI5, containing the highest bPPI loading (8.5 mmol g^−1^ amine content, Table 1), showed a 1.75-fold higher CO_2_ uptake (1.54 ± 0.05 mmol g^−1^) compared to unfunctionalized LWT (0.88 ± 0.08 mmol g^−1^) under these conditions. Notably, the DAC performance of LWT-bPPI5 is comparable to or surpasses that of many benchmark amine-functionalized sorbents, including silica, alumina, zeolites, mixed metal oxides, MOFs, and COFs, reported in the literature (fig. S10) (table S5). This improvement in CO_2_ capacity was most pronounced for LWT-bPPI samples synthesized using less than 1 equivalent of HClO_4_ (with respect to azetidine, Entry 5–10, Table 1) during functionalization, e.g., LWT-bPPI4 (0.86 eq., 1.25 mmol g^−1^), LWT-bPPI5 (0.5 eq., 1.54 mmol g^−1^), LWT-bPPI6 (0.25 eq., 1.38 mmol g^−1^), LWT-bPPI7 (0.5 eq., 1.42 mmol g^−1^), LWT-bPPI8 (0.5 eq., 1.45 mmol g^−1^), and LWT-bPPI9 (0.5 eq., 1.23 mmol g^−1^) (Fig. 5A, fig. S9). The amine efficiency also increases from 0.15 (for LWT) to 0.18 (for LWT-bPPI5) after the bPPI functionalization at 30°C under dry DAC conditions. Overall, the trend in CO_2_ uptake follows the amine content of the bPPI-grafted resins. These results support our hypothesis that lowering the acid-to-azetidine ratio to <1 enhances the efficiency of bPPI grafting from the LWT surface, ultimately yielding sorbents with a larger number of active amines and thus, enhanced DAC performance. We also explored the effect of the pristine LWT particle size on the grafting process. LWT-bPPI5 samples prepared using different particle size ranges of LWT (300–500 μm, <300 μm, and < 125 μm) under identical conditions showed the highest CO_2_ uptake for particles below 300 μm (fig. S9, C and D). This observation suggests that reducing the particle size of pristine LWT to an optimal range facilitates more effective grafting, likely by improving pore accessibility for functionalization.

**Fig. 5.**
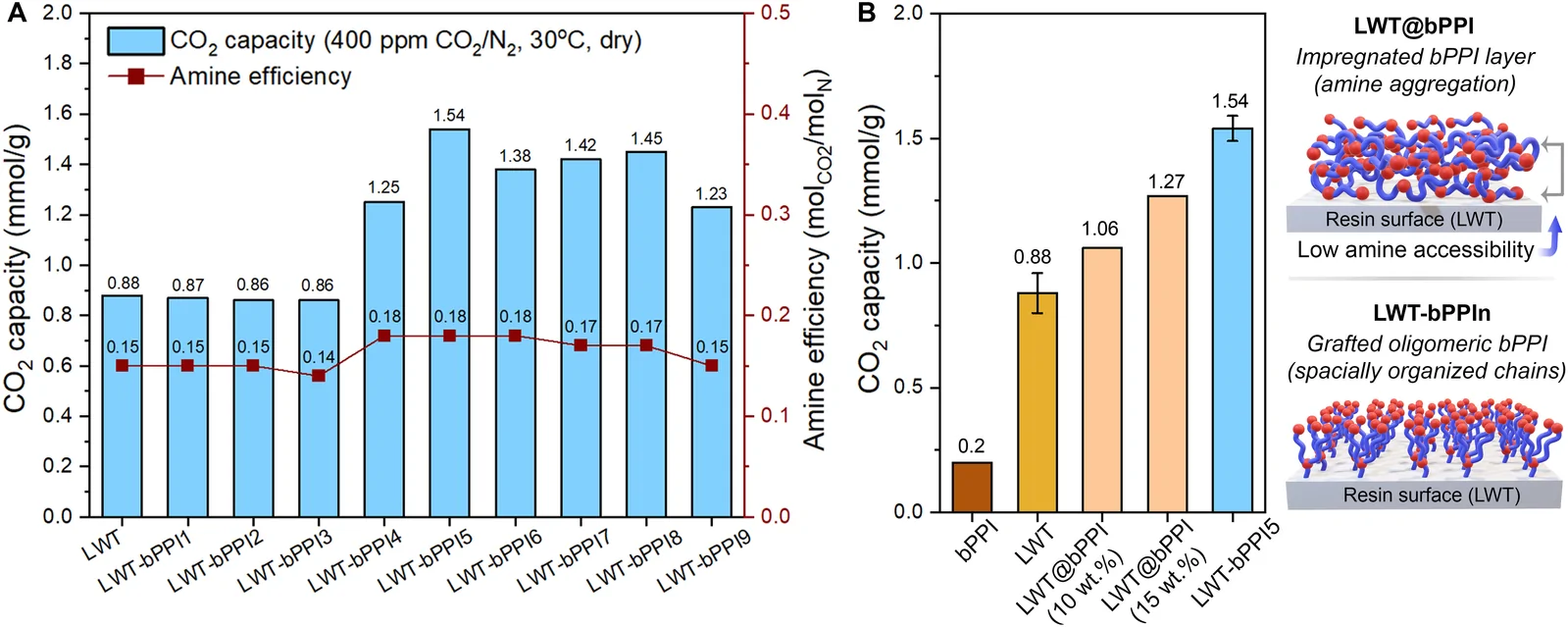
Enhancement of CO_2_ uptake and amine efficiency in bPPI-grafted LWT resins. (**A**) Thermogravimetric analysis (TGA) of pristine LWT and bPPI grafted LWT resins, i.e., LWT-bPPI1 to LWT-bPPI9 under 400 ppm CO_2_/N_2_ at 30°C (6 h adsorption, followed by activation at 80°C under N_2_ flow). Bar chart depicting the CO_2_ uptake capacities of LWT and LWT-bPPIn, along with their corresponding amine efficiencies. (**B**) Comparative CO_2_ sorption performance of the bPPI-grafted resin LWT-bPPI5, pristine bPPI polymer, pristine LWT, and physically impregnated LWT@bPPI samples (10 and 15 wt.% bPPI loading) under 400 ppm CO_2_/N_2_ at 30°C (6 h adsorption followed by regeneration at 80°C under N_2_ flow). Pictorial illustration of physically impregnated LWT@bPPI, and in situ grafted LWT-bPPIn sorbents are shown on the right [red spheres indicate amine functionalities, blue curvy lines indicate aliphatic carbon chains in the poly(amine)s].

We additionally compared the DAC performance of LWT-bPPI5 with pristine bPPI polymer (M_w_: ∼900 g mol^−1^) and also physically impregnated LWT@bPPI sorbents (10 wt.% and 15 wt.% bPPI loading) (Fig. 5B) (fig. S11). The pristine bPPI polymer exhibited a very low CO_2_ uptake capacity (0.2 mmol g^−1^), underscoring the crucial role of the porous support in enhancing the accessibility of amine sites toward CO_2_ capture, as the presence of amine functionalities alone does not guarantee efficient DAC performance. Similarly, physically impregnated samples also showed lower CO_2_ uptake (1.06, 1.27 mmol g^−1^, respectively) than LWT-bPPI5 (1.57 mmol g^−1^) and the other batches of bPPI grafted polymers (LWT-bPPIn, n = 5–9, 10–15% bPPI loading) under 400 ppm CO_2_/N_2_ at 30°C and 0% RH. This difference likely originates from the heterogeneous distribution and aggregation of physically impregnated higher molecular weight bPPI chains, which reduce amine accessibility (Fig. 5B). In contrast, the in situ “grafting-from” strategy generates short, covalently anchored bPPI oligomers with improved spatial distribution and higher amine accessibility, resulting in enhanced CO_2_ capture performance.

To evaluate whether this structural advantage persists at elevated CO_2_ concentrations, we examined the optimized material under simulated flue-gas conditions (15% CO_2_/N_2_, 40°C, 0% RH). Consistent with the improved amine efficiency, LWT-bPPI5 exhibited significantly high CO_2_ uptake reaching 3.34 mmol g^−1^ compared to 2.15 mmol g^−1^ for LWT (fig. S12). Importantly, LWT-bPPI5 achieves 50% of its total CO_2_ uptake within just 6 minutes (t_1/2_), demonstrating rapid sorption. Together, these findings highlight that precise control over the acid-to-azetidine ratio governs facile surface-grafting, which in turn dictates amine accessibility and CO_2_ capture performance over a broad range of operating conditions.

#### CO_2_ breakthrough experiments

Given that most of Earth’s land surface experiences temperatures below 25°C and relative humidity above 35%, assessing sorbent performance under such realistic conditions is essential for geologically flexible DAC technologies (*62*–*64*). We therefore carried out dynamic breakthrough experiments with LWT and LWT-bPPI5 under a broad range of temperatures from −20°C to 25°C and relative humidities ranging from 0% to 90% (Fig. 6, figs. S13 to S17). Breakthrough capacities and pseudo-equilibrium capacities were calculated at the times when the CO_2_ concentration reached 5% and 95% of C_0_ (i.e., 400 ppm CO_2_/N_2_), respectively (figs. S13 and S14). Under dry conditions, LWT and LWT-bPPI5 exhibited contrasting temperature-dependent behavior. For LWT, as the temperature decreased from 25°C to −20°C, the pseudo-equilibrium capacity gradually increased from 0.96 to 1.55 mmol g^−1^. Analysis of the breakthrough curves revealed that the breakthrough time increased modestly from 25°C to 15°C, beyond which it declined progressively toward −20°C, accompanied by a gradual broadening of the mass transfer zone (figs. S13A and S15A, table S4). This behavior suggests that although lower temperatures thermodynamically favor CO_2_ adsorption, the adsorption kinetics become increasingly diffusion-limited at sub-ambient temperatures. In sharp contrast, LWT-bPPI5 showed a continuous decrease in equilibrium capacity from 1.41 ± 0.16 to 0.20 mmol g^−1^ over the same range, a trend further supported by volumetric CO_2_ uptake measurements (fig. S18). Concurrently, the breakthrough time decreased substantially from ∼31 to ∼8 min with a progressive narrowing of the mass transfer zone across the same temperature range (figs. S14A and S16A, table S4). The shorter breakthrough time and diminished equilibrium capacity of LWT-bPPI5 at lower temperatures likely stems from the limited mobility of the grafted bPPI chains caused by both the reduced thermal energy and enhanced amine clustering via multidentate hydrogen bonding due to the higher amine density in the pores.

**Fig. 6.**
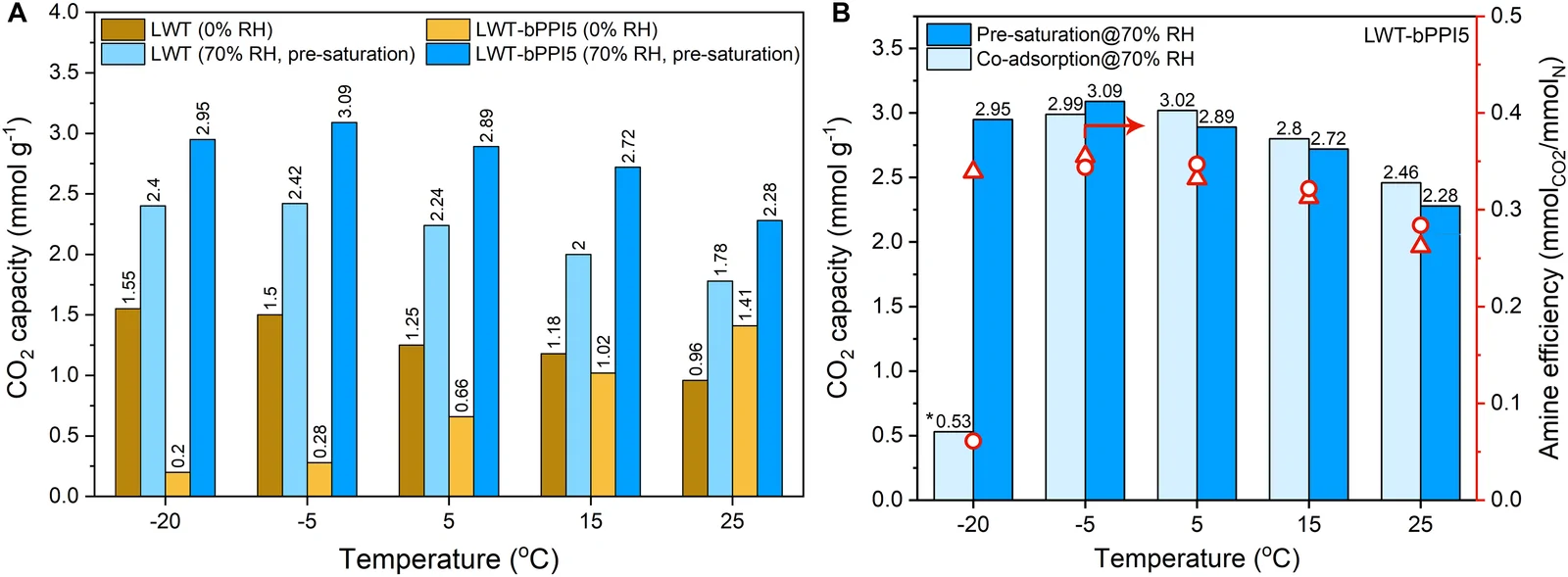
Temperature- and humidity-dependent CO_2_ uptake and amine efficiency of LWT-bPPI5 under DAC conditions. (**A**) Pseudo-equilibrium CO_2_ uptake (400 ppm CO_2_/N_2_) capacity of LWT-bPPI5 and LWT at diverse ambient and sub-ambient temperatures (25°C to −20°C) under dry (0% RH) and humid (70% RH) pre-saturation conditions. (**B**) Pseudo-equilibrium CO_2_ uptake capacity and the corresponding amine efficiencies of LWT-bPPI5 resin under pre-saturation (capacity: blue bar, amine efficiency: red triangle) and co-adsorption conditions (capacity: light blue bar, amine efficiency: red circle) at 70% RH and different temperatures. (*) indicates estimated CO_2_ uptake up to ∼12 h (C/C_0_ ≈ 0.92) of sorption at −20°C under co-adsorption conditions at 70% RH.

Under humid, pre-saturated conditions (70% RH), both sorbents displayed improved performance compared to dry conditions at the respective temperatures; however, LWT-bPPI5 consistently outperformed unfunctionalized LWT across the entire tested temperature window (Fig. 6A). For LWT, the equilibrium CO_2_ uptake increased to 1.78 mmol g^−1^ at 25°C under humid conditions, which is 1.85-times higher than under dry conditions. Previous studies by Reimer and coworkers investigated the water-induced swelling behavior of LWT using advanced solid-state NMR techniques and demonstrated that adsorbed water can substantially alter the local polymer environment and accessibility of amine functionalities (*65*). Inspired by these findings, we hypothesize that humidity-induced swelling of the LWT network improves access to previously inaccessible amine sites under DAC conditions. Consistent with this interpretation, the breakthrough time increased substantially under humid conditions (∼175 min) compared to dry conditions (∼103 min) at 25°C, indicating enhanced dynamic accessibility of the adsorption sites due to pore swelling (figs. S13B and S15B, table S4). Lowering of temperature further increased the equilibrium capacity to 2.4 mmol g^−1^ at −20°C. However, decreasing the temperature from 15 to −20°C resulted in shorter breakthrough times together with broader mass-transfer zones, indicating increasingly diffusion-limited adsorption behavior at lower temperatures (figs. S13B and S15B, table S4). This behavior likely arises because, although water-induced swelling of the LWT network increases the accessibility of amine sites, lowering the temperature simultaneously enhances water uptake within the pore structure. The excess adsorbed water partially fills the pores and creates more tortuous diffusion pathways, thereby slowing CO_2_ transport to the adsorption sites.

For LWT-bPPI5, the pseudo-equilibrium CO_2_ capacity increased from 2.28 mmol g^−1^ at 25°C to 3.09 mmol g^−1^ at −5°C, remaining high (2.95 mmol g^−1^) even at −20°C, with an amine efficiency of approximately 0.36 mmol CO_2_ mmol^−1^ N (Fig. 6B, fig. 14C). Interestingly, the breakthrough time also increased substantially under humid conditions, from ∼271 min at 25°C to ∼349 min at −5°C, compared to only 30 min and 8 min, respectively, under dry conditions. In addition, the breakthrough profiles remained steep and S-shaped with relatively narrow mass-transfer zones between 25 and − 5°C (figs. S14C and S16B, table S4), indicating rapid adsorption kinetics and that a large fraction of the equilibrium adsorption capacity remains dynamically accessible during adsorption. This observation strongly suggests that water influences the bPPI oligomers by disrupting amine-amine interactions while simultaneously inducing network swelling, synergistically enhancing amine accessibility toward CO_2_ and significantly reducing diffusional resistance in LWT-bPPI5. However, at −20°C, the breakthrough time decreased with a broad mass transfer zone, likely due to reduced bPPI chain mobility associated with low thermal energy and partial water condensation within the pores. Nevertheless, even under these conditions, LWT-bPPI5 maintains significantly higher dynamic CO_2_ capture performance than pristine LWT.

Across the full temperature range, LWT-bPPI5 exhibited 23–36% higher equilibrium uptake than LWT under 70% RH, demonstrating the beneficial role of bPPI functionalization in humid DAC-relevant atmospheres. In the co-adsorption method, where CO_2_ and water vapor were introduced simultaneously to a dry adsorbent, LWT-bPPI5 showed capacities similar to those of pre-saturated samples in the temperature range of −5°C to 25°C (Fig. 6B, fig. S14B). At 25°C and 70% RH, the CO_2_ uptake was 2.47 mmol g^−1^, which further increased to 3.02 mmol g^−1^ at 5°C (amine efficiency: 0.36 mmol CO_2_ mmol^−1^ N) accompanied by long breakthrough time and sharp breakthrough profile, indicating facile CO_2_ diffusion and rapid sorption kinetics. Notably, the breakthrough curve at −5°C exhibited 2.99 mmol g^−1^ uptake with a non-monotonic profile, an initial rapid rise, a distinct dip, and a subsequent rise to saturation (fig. S14B). This unusual behavior likely originates from competitive co-adsorption between H_2_O and CO_2_ during the initial stage. Initially, adsorbed water molecules partially occupy the pore environment and temporarily hinder CO_2_ diffusion and amine accessibility, resulting in the initial breakthrough increase. As hydration progresses, water disrupts amine-amine interactions and induces polymer swelling, enhancing amine accessibility and resulting in the subsequent decrease in breakthrough concentration before gradual saturation occurs. However, at −20°C, the co-adsorption experiments revealed a substantial reduction in breakthrough performance, indicating strong kinetic limitations. Complete saturation required extended exposure, resulting in a low uptake of 0.53 mmol g^−1^ of CO_2_ after ∼12 h (Fig. 6B, fig. S14B). Unlike the pre-saturation conditions, co-adsorption of H_2_O and CO_2_ into a mostly dry sorbent does not allow sufficient time for extensive amine hydration, suppressing chain mobility and hindering efficient CO_2_ diffusion at low temperatures. In pre-hydrated samples, by contrast, the polymer network remains swollen and the amines are already solvated, thereby enhancing chain mobility, and amine accessibility and enabling higher CO_2_ uptake, even at −20°C. At 25°C under co-adsorption conditions, LWT-bPPI5 maintains a stable pseudo-equilibrium CO_2_ capacity with a slight increase from 2.45 to 2.58 mmol g^−1^ as RH increases from 50% to 90%, indicating good tolerance to high humidity (fig. S17).

Having revealed temperature-dependent mobility limitations of the grafted bPPI chains under fixed humidity conditions, we next decoupled the humidity effect by systematically examining CO_2_ uptake as a function of relative humidity at 30°C and 5°C using dynamic vapor sorption (DVS Carbon) analysis. At 30°C, the CO_2_ uptake increases monotonically with increasing relative humidity from 0% to 70%. In contrast, at the lower temperature of 5°C, a decrease in CO_2_ uptake is observed when transitioning from dry conditions to 20% RH, followed by a steady increase at higher humidity levels (≥50% RH) (fig. S19). We attribute this behavior to insufficient water content at low RH to enhance amine mobility and accessibility, combined with competitive adsorption of water and CO_2_ at sub-ambient temperature. As a result, a slight reduction in CO_2_ capacity is observed under these conditions. Overall, LWT-bPPI5 exhibited striking moisture-induced enhancements in performance, with a ∼1.8-fold increase in CO_2_ capacity at 25°C and a remarkable ∼15-fold increase at −20°C relative to dry conditions (Fig. 6). Improved performance was achieved under both pre-saturation conditions, which simulates a sorbent state after direct steam-stripping desorption, and during CO_2_/H_2_O coadsorption, which simulates the sorbent after a desorption process that removes all water from the material in each cycle. Such dramatic improvements may arise from either (i) a mechanistic shift from carbamate formation (two amine sites to bind one CO_2_) under dry conditions to carbamic acid and/or bicarbonate pathways (one amine site to bind one CO_2_) under humid conditions, or (ii) water-induced network swelling and mobilization of the grafted bPPI chains that increases amine accessibility, or a combination of both (*66*). In this context, we carried out mechanistic investigations to elucidate the relative contributions of these pathways.

### Mechanistic investigation of CO_2_ adsorption under dry and humid conditions using DRIFTS

The in situ DRIFTS spectra of LWT and LWT-bPPI5 recorded under dry and humid CO_2_ environments provide valuable insights into the nature of adsorbed species and the underlying CO_2_ capture mechanism (Fig. 7, fig. S20). Under dry conditions (25°C, 0% RH), the characteristic stretching bands observed at around 1663, 1580 (ν_as_), 1552 (ν_as_), 1484, and 1428 (ν_s_) cm^−1^ are attributed to various symmetric, asymmetric stretching vibrations of the COO^−^ groups and 1407 and 1316 cm^−1^ are assigned for NCOO^−^ skeletal vibrations, while the peaks at 1632 (δ_as_), 1537 (δ_as_), and 1473 (δ_s_) cm^−1^ correspond to the bending vibrations of NH_3_^+^ groups (Fig. 7A, fig. S20A) (*17*, *59*, *67*). Under humid co-adsorption conditions (25°C, 75% RH), no significant spectral shifts or the emergence of new features was detected for either LWT or LWT-bPPI5, suggesting that the CO_2_ adsorption mechanism remains largely unchanged compared to the dry condition and continues to proceed via ammonium carbamate formation (Fig. 7B, fig. S20B) (*39*). However, the potential presence of bicarbonate (HCO_3_^−^) species under humid conditions merits exploration in the future using complementary techniques such as solid-state ^13^C NMR spectroscopy, as these species may form in low concentrations and exhibit weak IR absorption bands that make their identification challenging (*59*, *68*).

**Fig. 7.**
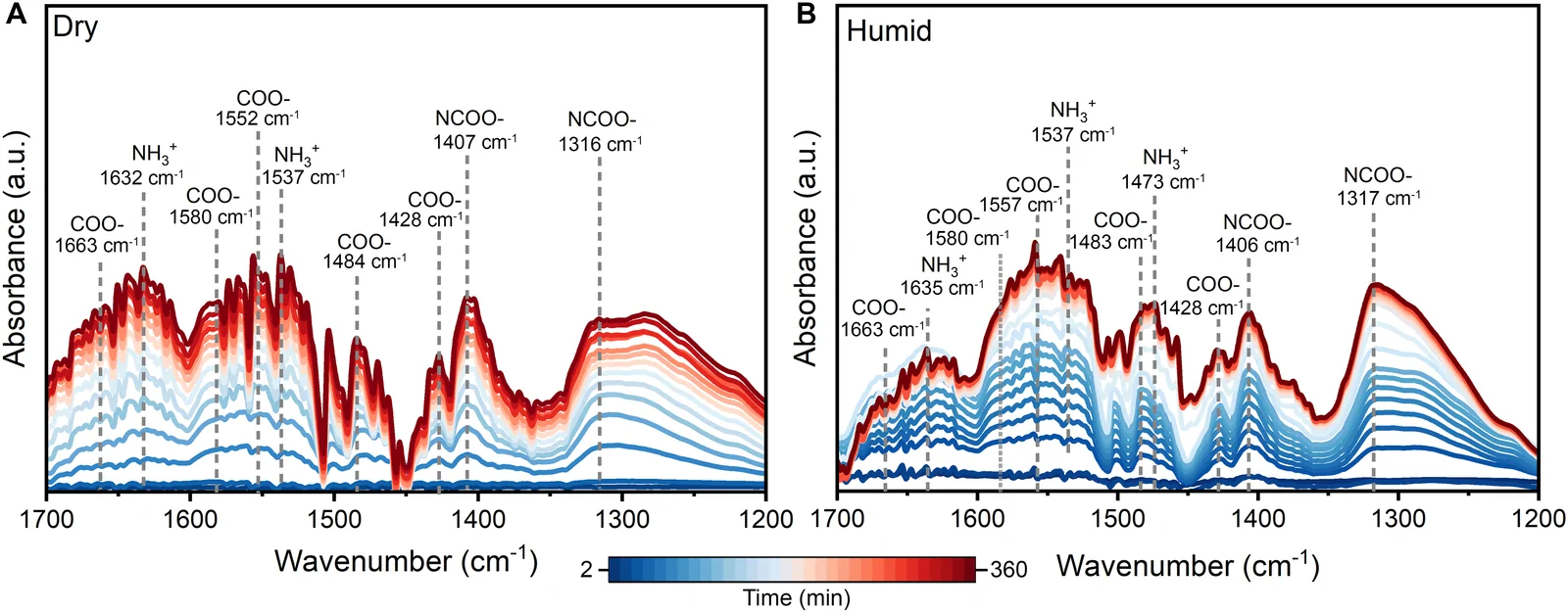
In situ DRIFTS analysis for CO_2_ sorption. In situ DRIFT spectra of LWT-bPPI5 as a function of adsorption time at 25°C under (**A**) dry and (**B**) humid conditions (75% RH). Adsorption conditions: 400 ppm CO_2_/N_2_; flow rate: 40 sccm; activation, 80°C under 60 sccm N_2_ for 2 h.

### Temperature programmed desorption studies

Alkyl ammonium carbamate ion pairs (R-NH_3_^+^⋯^−^OOC-NH-R) are known to exhibit strong chemisorption with higher binding energies than carbamic acid (R-HN-COOH). To gain a qualitative understanding of the relative contributions of strong chemisorption and weak chemisorption/physisorption to the overall CO_2_ uptake in LWT and LWT-bPPI5 under both dry and humid conditions, we conducted temperature-programmed desorption (TPD) analyses (figs. S21 and S22). After exposing the samples to 400 ppm CO_2_/N_2_ at 25°C under both dry and humid (70% RH, pre-saturation) conditions, the desorption was carried out using a controlled temperature ramp of 0.5°C min^−1^. CO_2_ desorbed below 25°C was assigned to weak chemisorption/physisorption, whereas desorption above 25°C was assigned to strong chemisorption, primarily arising from ammonium carbamate formation (*59*).

For unfunctionalized LWT, the contribution from weak CO_2_-sorbent interaction increased from 23% to 38% under dry conditions and from 15% to 33% under humid conditions (70% RH) as the temperature decreased from 25°C to −20°C (figs. S21 and S22). This trend likely originates from the enhanced contribution of physisorption at lower temperatures (*38*). In contrast, LWT-bPPI5 showed reduced contributions for weak CO_2_-sorbent interaction (physisorption), rising only from 6% to 12.5% under dry conditions and from 7% to 25% under humid conditions over the same temperature range. This difference arises from the denser amine packing in LWT-bPPI5, which favors alkyl ammonium carbamate formation, whereas the higher surface area, pore volume, and lower amine loading in LWT lead to more dispersed amine sites, leading to a larger fraction of weakly bound CO_2_. This trend is supported by desorption energy (E_n_) values, i.e., LWT-bPPI5 exhibits a higher E_n_ (105.6 kJ mol^−1^) than LWT (75.8 kJ mol^−1^) (fig. S23) at 25°C and 70% RH (*69*). Nonetheless, strong chemisorption remains dominant (>60%) for both materials under all conditions, confirming that alkyl ammonium carbamate formation is the primary CO_2_ binding mechanism, consistent with the in situ DRIFTS results (figs. S21 and S22).

### Oxidative stability and recyclability

To preliminarily evaluate the oxidative stability of the sorbents, both LWT and LWT-bPPI5 samples were subjected to an accelerated aging test by exposing them to air (21% O_2_/N_2_) at 80°C for 144 hours. Following this oxidative treatment with 48 h intervals, their CO_2_ adsorption capacities were evaluated using dynamic vapor sorption (DVS Carbon) at 30°C, 70% RH under 400 ppm CO_2_/N_2_ using pre-saturation method (Fig. 8). LWT exhibited a pronounced decline in adsorption capacity, losing approximately ∼35% of its initial CO_2_ uptake after 144 h of oxidation. In contrast, the LWT-bPPI5 sample showed only a minor reduction of about ∼18% under identical conditions. Exposure to humid air (75% RH) at 80°C accelerated the aging of LWT, resulting in an ∼35% loss in CO_2_ capacity within 24 h, whereas LWT-bPPI5 exhibited a much smaller reduction of ∼10% (fig. S24). In situ DRIFTS of oxidized LWT revealed strong bands at 1700–1720 cm^−1^, likely corresponding to aldehydes, along with features at 1690–1650 cm^−1^ attributed to C=N and C=O species (amide, carboxylate, or urea) (figs. S25A and S26). These oxidation signatures were far weaker for LWT-bPPI5, confirming significantly reduced amine oxidation (fig. S25B). These results clearly demonstrate that grafting branched bPPI onto the LWT framework significantly enhances its resistance to oxidative degradation. Consistent with previous reports showing the superior oxidative stability of PPI relative to other poly(amine) systems, we propose that the chemically grafted bPPI oligomers generate a protective local environment around the benzylic anchoring sites within the resin network. This local shielding effect likely limits direct oxygen access to the vulnerable benzylic carbons, thereby suppressing oxidative degradation pathways and enhancing the long-term stability of the sorbent (*45*, *46*, *70*, *71*).

**Fig. 8.**
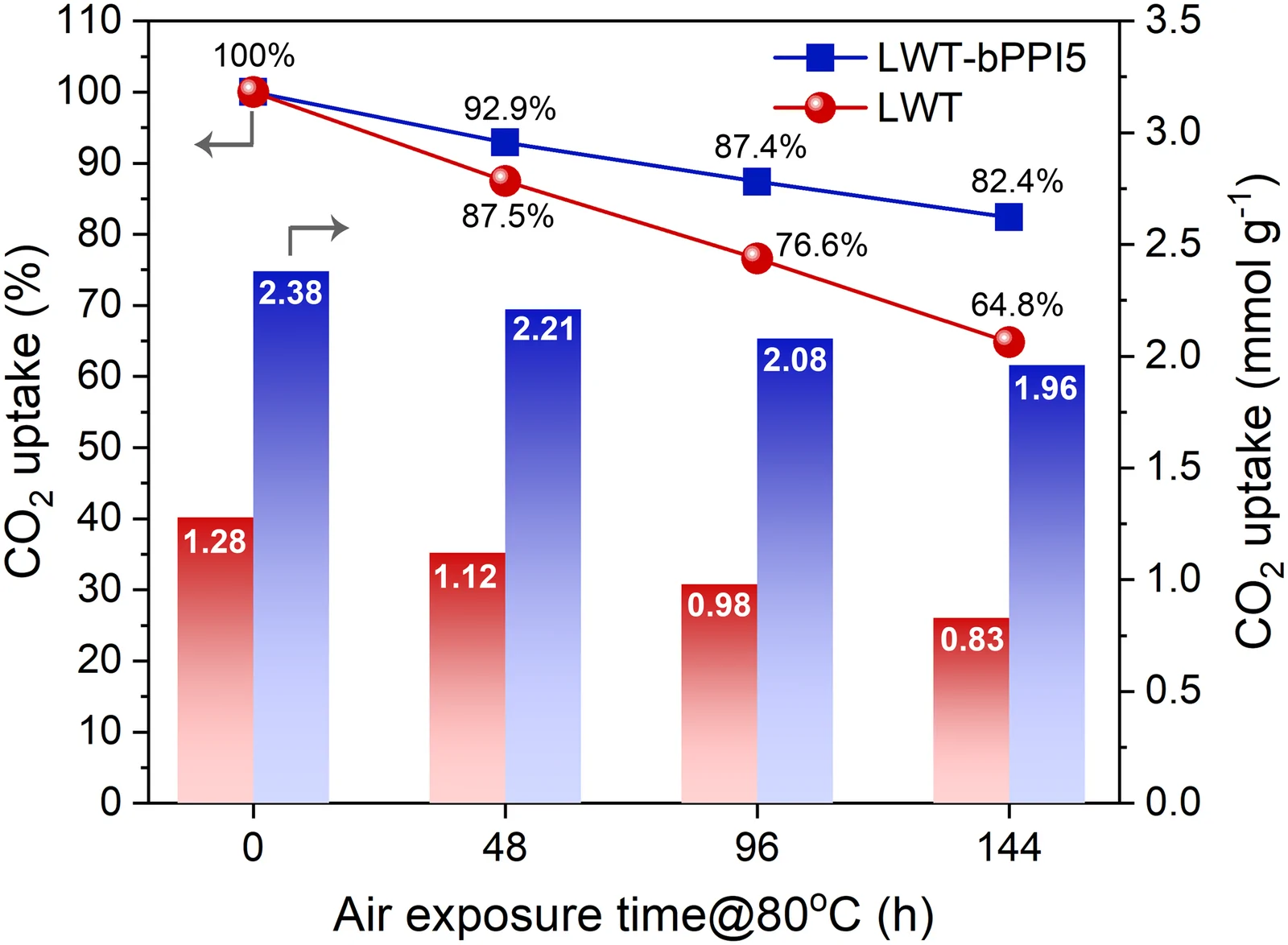
Oxidative stability of LWT and LWT-bPPI5. Bar chart showing the CO_2_ uptake capacities in mmol g^−1^ of LWT (red bar) and LWT-bPPI5 (blue bar) under 400 ppm CO_2_/N_2_, 70% RH (pre-saturation method) at 30°C after different time of ageing under dry air (21% O_2_/N_2_) at 80°C. The decrease in the adsorption capacities in % of LWT and LWT-bPPI5 resins were shown in red circles and blue squares, respectively. Data was measured via DVS Carbon.

TGA cycling tests confirmed the excellent regenerability of LWT-bPPI5, which maintained a CO_2_ uptake capacity of ∼1.3 mmol g^−1^ over 19 rapid adsorption-desorption cycles (3 h adsorption at 30°C under 400 ppm CO_2_/N_2_, followed by 15 min regeneration at 80°C under dry DAC conditions; Fig. 9A, fig. S27). To assess the cycling stability of LWT-bPPI5 under humid conditions (70% RH) and both ambient (25°C) and sub-ambient (5°C) temperatures, fixed-bed breakthrough experiments were carried out using consecutive adsorption-desorption temperature-swing cycles under simulated air (400 ppm CO_2_/N_2_; Fig. 9B, fig. S28). After each adsorption step (400 ppm CO_2_/N_2_ with a flow rate of 40 sccm), the sorbent was regenerated at 80°C under a continuous N_2_ flow (60 sccm) for 1 h to ensure complete desorption of CO_2_. The breakthrough profiles demonstrated that LWT-bPPI5 maintained an almost constant pseudo-equilibrium CO_2_ adsorption capacity (C/C_0_ = 0.95) of ∼2.45 mmol g^−1^ per cycle over 10 cycles at 25°C and 70% RH, and ∼ 2.95 mmol g^−1^ per cycle over 10 cycles at 5°C and 70% RH. The negligible loss in uptake capacity over 20 cycles and the nearly identical FTIR spectral features and nearly unchanged N-content (8.7 mmol g^−1^) obtained from the elemental analysis of the pristine and the recycled LWT-bPPI5 samples highlights the promising stability of LWT-bPPI5, supporting its significant potential for durable CO_2_ capture under both ambient and sub-ambient DAC conditions (fig. S29).

**Fig. 9.**
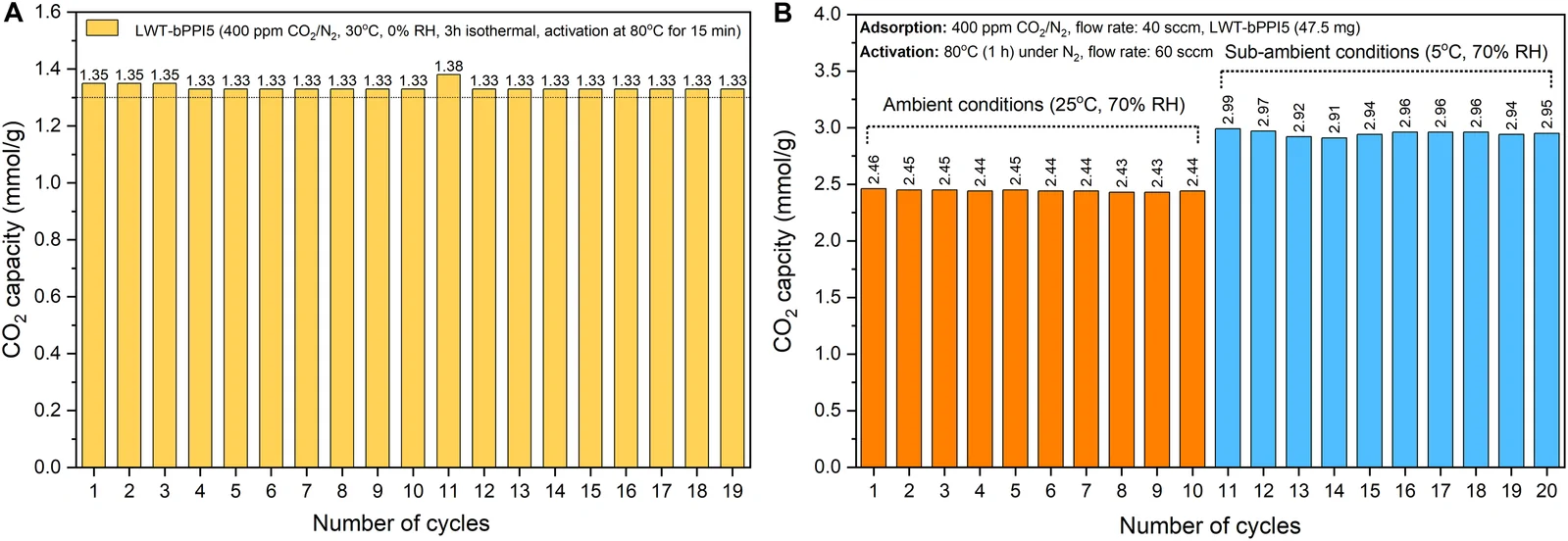
Cycling stability of LWT-bPPI5 under ambient and sub-Ambient DAC conditions. (**A**) Cycling Performance of LWT-bPPI5 under 400 ppm CO_2_/N_2_ at 30°C (3 h adsorption under 400 ppm CO_2_/N_2_ flow followed by 15 min regeneration at 80°C under N_2_ flow). (**B**) Pseudoequilibrium (at C/C_0_ = 0.95) capacities of LWT-bPPI5 measured via fixed bed breakthrough analysis over 20 cycles under ambient and sub-ambient co-adsorption conditions: 10 cycles under 70% RH at 25°C, 10 cycles under 70% RH at 5°C, (flow rate: 40 sccm 400 ppm CO_2_/N_2_). After each adsorption cycle, the sorbent was regenerated at 80°C under a nitrogen flow of 60 sccm.

While the material exhibits excellent stability under DAC-relevant conditions, practical carbon capture operating conditions often involve higher CO_2_ partial pressures and repeated thermal cycling. We therefore evaluated the long-term durability of LWT-bPPI5 under simulated flue-gas condition by conducting a comparative long-term recycling study in TGA for LWT and LWT-bPPI5 over 150 adsorption-desorption cycles (1 h adsorption at 40°C followed by 1 h desorption at 80°C under air) under simulated flue-gas (15% CO_2_/N_2_, 0% RH) (Fig. 10) (fig. S30). The bare LWT exhibits a more pronounced loss in CO_2_ uptake capacity than LWT-bPPI5. Linear fitting of the capacity decay revealed a degradation rate of 0.006 mmol g^−1^ cycle^−1^ for LWT-bPPI5, compared to 0.016 mmol g^−1^ cycle^−1^ for LWT (exponential fitting) (fig. S31). In contrast, when regeneration was performed under N_2_ at 80°C, LWT-bPPI5 showed no measurable loss in performance, while pristine LWT exhibited only ∼2% loss over 50 adsorption-desorption cycles, clearly indicating that the capacity decay observed during regeneration under air primarily originates from oxidative degradation of the amine functionalities by O_2_ rather than thermal degradation (fig. S30). Furthermore, the combustion TGA analysis of LWT-bPPI5 after 150 adsorption-desorption cycles under air revealed excellent retention of amine loading, with only ∼2.5 wt.% loss (fig. S32).

**Fig. 10.**
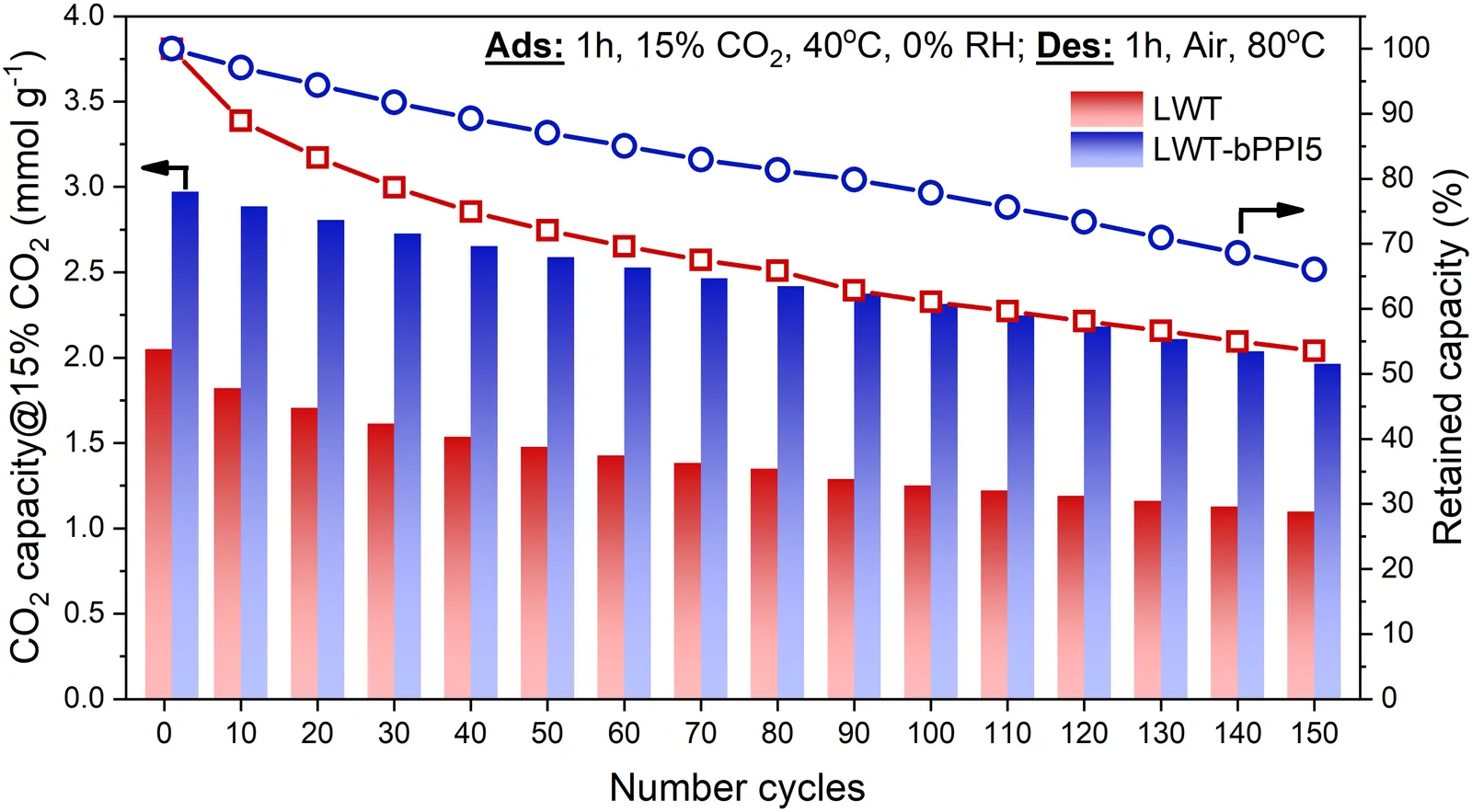
Recyclability of LWT and LWT-bPPI5 under simulated flue gas conditions and regeneration under air. Thermogravimetric analysis of CO_2_ uptake (mmol g^−1^, in bar plots) and retained capacity (%, in points and lines) of LWT (red) and LWT-bPPI5 (blue) under simulated flue gas conditions (15% CO_2_/N_2_ at 40°C, 0% RH) at different numbers of adsorption-desorption cycles. The adsorption-desorption cycle consists of 1 h adsorption at 40°C under followed by 1 h 15% CO_2_/N_2_ and desorption at 80°C under air (21% O_2_/N_2_).

In addition to conventional thermal regeneration, steam-assisted regeneration is considered a promising and energy-efficient alternative for DAC processes. To evaluate the hydrothermal robustness of the materials, both LWT and LWT-bPPI5 were exposed to continuous steam generated from boiling deionized water for 72 h. After steam treatment, the samples were thoroughly dried, re-activated, and their CO_2_ uptake capacities were measured by TGA under DAC-relevant conditions (400 ppm CO_2_/N_2_) at 15°C and 54% relative humidity (pre-saturated). Notably, LWT-bPPI5 retained the majority of its CO_2_ capture capacity after prolonged steam exposure, exhibiting only ∼8% loss (fig. S33). This behavior highlights the strong covalent attachment of the bPPI oligomers to the resin framework, which effectively mitigates amine leaching, structural rearrangement, or degradation, issues commonly encountered in non-covalently grafted or physically impregnated PEI-based sorbents under steam treatment. The excellent steam stability of LWT-bPPI5 therefore underscores its structural robustness and practical potential for integration into steam-assisted regeneration schemes, further enhancing its suitability for long-term DAC applications.

## DISCUSSION

In summary, we developed a facile and scalable one-step strategy to graft branched poly(propyleneimine) oligomers onto primary amine-functionalized commercial porous resins via acid-catalyzed in situ polymerization of azetidine. The specific N content of the optimized resin (LWT-bPPI5) increased from 5.7 ± 0.2 to 8.7 ± 0.1 mmol g^−1^, resulting in up to a 75% enhancement in CO_2_ uptake under dry DAC conditions at 30°C. Under humid conditions (70% RH), LWT-bPPI5 achieved capacities of ∼2.5 mmol g^−1^ at 25°C and ∼ 3.0 mmol g^−1^ at −5 to −20°C, corresponding to an improvement of ∼36% compared to pristine LWT. In addition, the sorbent exhibited excellent oxidative and steam stability, recyclability, and regeneration under mild conditions (80°C). Under flue-gas conditions (15% CO_2_, 40°C), LWT-bPPI5 achieved a 55% increase in CO_2_ uptake with excellent cycling performance over 150 cycles compared to pristine LWT. The present study thus demonstrates a promising route for fabricating bPPI-grafted porous resins with excellent recyclability and high CO_2_ capacity under DAC and flue-gas conditions, across a broad range of temperatures, and humidities (fig. S10, table S5). As these commercial resins are currently amongst the leading candidate materials for large scale DAC deployment, this one step strategy could provide a boost to near-term DAC deployments.

Nonetheless, several aspects warrant further investigation. Although the current approach has been validated at the gram-scale, process optimization at the kilogram-to-ton scale will be crucial for further industrial translation. Milder acid catalysts (HCl, HBr, CH_3_SO_3_H, etc.) can be utilized and this will make the process more industrially friendly (*53*). Efforts are already underway in our laboratory to extend this strategy to other primary- and secondary-amine-functionalized porous supports to further generalize the bPPI-grafting methodology. In this context, preliminary results demonstrate that the strategy can be successfully applied to another commercially available primary-amine-containing PS-DVB resin, Purolite A110. Upon bPPI grafting, the N content increased by ∼34% (7.1 mmol g^−1^ for pristine resin to 9.5 mmol g^−1^ for bPPI grafted resin, table S1), accompanied by a ∼1.5-fold enhancement in CO_2_ uptake capacity from 1.15 mmol g^−1^ to 1.73 mmol g^−1^ under 30°C, 400 ppm CO_2_/N_2_, and 0% RH (fig. S34). Long-term field tests of sorption/desorption cycling under realistic conditions (400 ppm CO_2_/air and 15% CO_2_/air) are needed to further evaluate operational stability. Complementary techniques such as solid-state ^13^C NMR should be employed to probe the formation of bicarbonate and carbamate species and further elucidate the CO_2_ adsorption mechanism(s). Finally, detailed in situ spectroscopic studies on the oxidative degradation pathways of both pristine and bPPI-grafted LWT under varying environmental conditions would further clarify their enhanced stability. Overall, the present work provides a foundation for the development of other next-generation poly(amine) sorbents for practical DAC applications.

## MATERIALS AND METHODS

### Chemicals

All reagents were acquired from commercial sources and used as received. Lewatit VP OC 1065 (LWT) ion exchange resin with Lot No. BCCL5490 (Product No.: 94136–100G-F), aqueous perchloric acid (aq. HClO_4_, ∼70%), and anhydrous methanol (99.8%) were obtained from Millipore Sigma. Purolite A110 ion exchange resin (N047U/24/6, Product code: 67009, S.R.#: 6835) was procured from Ecolab. Azetidine (purity ≥99%) was obtained from Ambeed Chemicals. Reagent grade methanol, and ammonium hydroxide were obtained from VWR and Fisher Scientific, respectively. Branched poly(propylene imine) (M_w_: ∼900 g mol^−1^) used for the physical impregnation with LWT was synthesized in lab (*47*).

### Synthesis of branched poly(propyleneimine)-grafted porous polymeric resin (LWT-bPPIn)

The present invention provides a method for the synthesis of chemically grafted branched poly(propyleneimine) (bPPI) onto amine-bearing polymeric resins, such as, commercial poly(styrene-divinylbenzene) resin (PS-DVB resin), specifically Lewatit VP OC 1065 (LWT). First, 50 mg of commercial amine functionalized poly(styrene-divinylbenzene) resin, i.e., LWT (in the form of powder, particle size <300 μm, vide infra), was dispersed in 2 mL anhydrous methanol in 25 mL Schlenk tube followed by addition of 130–364 μL of azetidine (i.e., ∼7 to ∼20 equivalents with respect to amine groups present in LWT) under argon atmosphere. The mixture was stirred gently (∼300 rpm) for 30 min under room temperature (21–25°C) to ensure uniform dispersion and to promote adsorption of azetidine onto the resin. Subsequently, 41.6–615.5 μL (i.e., 0.25 to 3.7 equivalent with respect to azetidine) of perchloric acid (∼70% HClO_4_ aq.) was added slowly to the stirred reaction mixture. The reaction vessel was sealed, and the mixture was heated to 70°C (ramp rate: ∼1°C min^−1^, stirring speed of 350 rotation per minute with a 1 in. by 5/16 in. stirring bar) for 48 hours to facilitate in situ polymerization of azetidine, yielding branched poly(propyleneimine) (bPPI) chains covalently anchored to the resin framework. Upon completion, the reaction mixture was cooled down to room temperature and the resulting product, designated as LWT-bPPIn (where n = 1–9), was isolated as an off-white solid. The solid was washed thoroughly with aqueous ammonium hydroxide followed by methanol to remove residual acid and any unbound or loosely adsorbed bPPI species. The purified product was dried under dynamic vacuum at ambient temperature overnight. The detailed reagent compositions for each LWT-bPPIn variant are provided in Table 1. We further scaled up the reaction to 300 mg for the synthesis of LWT-bPPI5 while maintaining the exact stoichiometric ratios of the reagents, catalysts, and solvent (300 mg LWT, 780 μL azetidine, 499.2 μL 70% HClO_4_, 12 mL anhydrous methanol in a 50 mL Schlenk tube). The reaction time (48 h) and temperature (70°C) were kept unchanged (obtained bPPI-grafted resin after drying at room temperature under vacuum for overnight: 370–380 mg). By maintaining the stoichiometry, the reaction can be scaled up to a few gram-scale.

### Synthesis of branched poly(propyleneimine)-grafted Purolite A110

Purolite A110 is another commercially available weak base ion-exchange resin with primary amine functionalized poly(styrene-divinyl benzene) backbone. We procured Purolite A110 resin (Purolite A110 N047U/24/6, Product code: 67009, S.R.#: 6835) directly from Ecolab. bPPI grafting on Purolite A110 was performed following the optimized protocol analogous to that used for Lewatit VP OC 1065 (*vide supra*). Briefly, Purolite A110 (300 mg, powder, < 300 μm) was dispersed in anhydrous methanol (12 mL) in a Schlenk tube, followed by the addition of azetidine (1006 μL, 14.93 mmol, 7 equiv. relative to amine groups) under Ar. After stirring at room temperature for 30 min, perchloric acid (∼70% HClO_4_ aq., 644 μL, 0.5 equiv. relative to azetidine) was added dropwise. The sealed reaction mixture was heated at 70°C for 48 h for in situ azetidine polymerization. The resulting Purolite A110-bPPI was isolated, thoroughly washed with aqueous ammonium hydroxide and methanol, and dried under vacuum. CHN elemental analysis showed an increase in N content from 7.11 mmol g^−1^ (pristine) to 9.50 mmol g^−1^ (bPPI-grafted).

### Characterization

The structural characterization of the pristine and bPPI-grafted resins was carried out through various spectroscopic and microscopic techniques. Diffuse reflectance infrared Fourier transform spectroscopy (DRIFTS) analysis was carried out using Thermo Scientific Nicolet iS50 Fourier transform infrared (FTIR) spectrometer and an in situ DRIFTS cell. Before the measurement, ∼50 mg of resin sample was activated under a constant nitrogen flow of 60 standard cubic centimeters per minute (sccm) at 110°C for 2 h, then cooled to 25°C, and spectra were collected under a N_2_ atmosphere. Solid-state cross-polarization/magic angle spinning (CP/MAS) ^13^C nuclear magnetic resonance (NMR) using 400 MHz Bruker Avance III with a 9.5 T wide-bore magnet. Dry powdered resin samples were packed into 4 mm zirconia rotors. Adamantane and glycine were used as reference standards prior to analysis and the MAS rate was maintained at 12 kHz. The X-ray photoelectron spectroscopic (XPS) analyses of the resins were carried out using Thermo Scientific K-Alpha XPS instrument. The CHN elemental analysis of the resins were conducted at Atlantic Microlab, Inc. Thermal stability of all the resins was checked using a thermogravimetric analyzer, Q500 TA instrument under air. Before each analysis, the dried samples were activated at 110°C for 1 h. The textural characterizations of the resins were carried out using N_2_ physisorption isotherm analysis using Autosorb iQ/Anton Paar instrument. Prior to the measurement, all of the sorbent materials were degassed at 80°C under vacuum for 6 h to remove adsorbed CO_2_ or moisture. The Brunauer-Emmett-Teller (BET) specific surface area was estimated in the relative pressure (p/p_0_) range of 0.05–0.30. The total pore volume was calculated based on the N_2_ sorption at a p/p_0_ of 0.95, and the Barrett-Joyner-Halenda (BJH) model was used to calculate the pore size distribution of the sorbent material with Quantachrome ASiQwin software. The morphological analysis was carried out using Hitachi SU8230 Field emission scanning electron microscopy (FESEM) with accelerating voltage of 10 kV. Powdered resin samples were sprinkled onto carbon tape fixed on an aluminum stub. A ∼10 nm gold-palladium coating was applied by sputtering prior to imaging.

### CO_2_ uptake study and recyclability

We evaluated the preliminary direct air capture of CO_2_ (DAC) performances of various resins thermogravimetrically at 30°C under dry conditions using Q500 TA instrument. Prior to each measurement, the samples (∼10 mg) were activated at 80°C with a ramp rate of 10°C min^−1^ followed by isothermal at 80°C for 3 h under a constant N_2_ flow (90 sccm). The samples were subsequently cooled to 30°C using the same ramp rate and allowed to equilibrate for 5 min. Adsorption was then carried out isothermally for 6 h at 30°C under 400 ppm CO_2_/N_2_. The CO_2_ uptake capacities of all the amine grafted resins were estimated as the mass gained during the 6 h of adsorption. CO_2_ desorption was performed by heating the samples to 80°C at a rate of 10°C/min, followed by an isothermal hold of 15–60 min under a N_2_ flow of 90 sccm. The amine efficiencies of both the pristine and bPPI-grafted resins were determined from the amount of CO_2_ adsorbed relative to the total amine loading on the polymer, as obtained from CHN analysis. The recyclability of the bPPI-grafted resins (e.g., LWT-bPPI5, ∼10 mg) was evaluated over 19 rapid adsorption-desorption cycles using a Q500 TA instrument under 400 ppm CO_2_/N_2_ dry conditions at 30°C. Each cycle comprised 3 h of adsorption under 400 ppm CO_2_/N_2_ (flow rate: 90 sccm) at 30°C, followed by 15 min of activation at 80°C under N_2_ flow (90 sccm). The measurements under simulated flue gas mixtures were carried out using a Q500 TA instrument, using two gas streams, Gas 1, a dry N_2_ cylinder and Gas 2, a dry mixture of certified 15% CO_2_ 85% N_2_, both set at 100 mL min^−1^. Prior to each measurement, the samples (∼20 mg) were activated at 100°C with a ramp rate of 10°C min-1 followed by an isothermal step at 100°C for 1 h under a constant N_2_ flow. The samples were subsequently cooled down to 40°C using the same ramp rate and allowed to equilibrate for 15 min. Adsorption was then carried out isothermally for 6 h at 40°C under 15% CO_2_ 85% N_2._ Long 150 air cyclic test experiments for the LWT and LWT-bPPI5 samples were conducted on a Discovery TGA 550, from TA instruments using two gas streams: Gas 1, a dry, CO_2_-free Air cylinder and Gas 2, a mixture of certified 15% CO_2_ 85% N_2,_ both set at 100 mL min^−1^. ∼20–25 mg sample was first heated to 80°C at a ramp rate of 10°C/min under Gas 1 atmosphere and held at this temperature for 60 min. Subsequently, the sample was cooled down to 40°C, and kept under isothermal conditions for 15 min, after which the gas was switched to Gas 2 for 60 min to collect the CO_2_ uptake. This adsorption-desorption cycle was repeated 150 times in total.

### CO_2_ dynamic breakthrough analysis

Breakthrough experiments were performed in a custom-built fixed-bed reactor using dried resin samples (90–100 mg) packed in a 1/4″ stainless-steel tube (∼5 cm length, 4 mm i.d.), sealed with glass wool. A coiled 1/8″ stainless-steel tube upstream allowed controlled pre-heating or pre-cooling of the gas feed, and the reactor bed was immersed in a refrigerated circulating bath (water:ethylene glycol, 1:1 v/v) connected to a Julabo CD-600F chiller (−20 to 80°C). Gas flow rates were precisely controlled with Alicat mass flow controllers. Samples were pre-activated at 80°C for 2 h under N_2_ (60 sccm). Breakthrough tests were then conducted with dry or humid (0% and 70% RH) 400 ppm CO_2_/N_2_ (40 sccm), with humidity regulated by a WETSYS/SETARAM humidifier and outlet CO_2_/H_2_O concentrations monitored using a LI-850 (LI-COR) infrared gas analyzer. For co-adsorption humid experiments, the resin was directly exposed to humid 400 ppm CO_2_/N_2_ (40 sccm) immediately after activation. On the other hand, for presaturation humid experiments, the resin was first pre-humidified with 70% RH/N_2_ (40 sccm) at the target temperatures (−20°C to 25°C), followed by adsorption under humid 400 ppm CO_2_/N_2_ (40 sccm).

### In situ DRIFTS study for CO_2_ adsorption

In situ DRIFTS measurements were carried out on a Nicolet iS10 FT-IR spectrometer to monitor surface species formed under simulated DAC conditions. Prior to each experiment, ∼50 mg of resin was loaded into the DRIFTS cell and activated at 110°C under a continuous flow of dry N_2_ (60 sccm) for 2 h. After activation, the temperature was reduced to 25°C and the sample was allowed to equilibrate for 30 min. Subsequently, the activated sorbents were exposed to a CO_2_ stream (400 ppm in N_2_) under either dry or humid conditions at a flow rate of 40 sccm. For humid experiments, the 400 ppm CO_2_/N_2_ was passed through a bubbler containing saturated sodium chloride solution at room temperature, generating a stable relative humidity of 75%. IR spectra were collected periodically during gas exposure (64 scans per spectrum, 4 cm^−1^ resolution). To isolate the vibrational features associated specifically with CO_2_ adsorption, each spectrum recorded during CO_2_ dosing was baseline-corrected using the spectrum of the activated sample acquired immediately before the introduction of 400 ppm CO_2_/N_2_.

### Temperature programmed desorption (TPD) study

TPD experiments were conducted immediately after the CO_2_ breakthrough runs at 25°C, 70% RH. Once pseudo-equilibrium was reached (C/C_0_ = 0.95), the inlet stream of 400 ppm CO_2_/N_2_ (40 sccm) was switched to pure N_2_ (60 sccm), and the sample was purged for 45 minutes at 25°C. Subsequently, the temperature was ramped to 80°C at controlled heating rates (0.3–0.7°C min^−1^). Throughout the process, outlet CO_2_ concentrations were continuously monitored using a LI-850/LI-COR analyzer to obtain the desorption profiles. The energy of CO_2_ desorption of LWT and LWT-bPPI5 under 70% RH at 25°C were determined using the TPD analysis method described by Cvetanović and Amenomiya (*69*), applying the following equation

$$2\text{ln}\left(T_{m}\right)-\text{ln}\beta =\frac{E_{d}}{RT_{m}}+\text{ln}\frac{E_{d}}{\mathrm{AR}}$$ (2)

where *T*_m_ is the peak-maximum temperature in the CO_2_ TPD profile, β is the heating rate, *E*_*d*_ is the desorption energy, A is the pre-exponential factor, and R is the universal gas constant (8.314 J mol^−1^ K^−1^). Using the experimentally measured *T*_m_ values at different β, *E*_*d*_ (kJ mol^−1^) can be determined.

### Oxidation study

Three long cyclic oxidative studies for the LWT and LWT-bPPI5 samples were conducted using a DVS Carbon instrument (Surface Measurement Systems) using two gas streams: Gas 1, a mixture of 400 ppm CO_2_/N_2_, and Gas 2, dry CO_2_-free air. Prior to first adsorption cycle, the sample was first heated to 80°C at a ramp rate of 5°C min^−1^ under dry CO_2_-free air and held at this temperature for 30 min. Subsequently, the sample was cooled to 30°C, after which humidity was introduced to achieve 70% relative humidity (RH) at 30°C. The sample was equilibrated under humid air until the mass change rate (dm/dt) was below 0.001 mg min^−1^ for at least 10 min. Following humid equilibration, the gas stream was switched to Gas 1 (400 ppm CO_2_) while maintaining 30°C and 70% RH. CO_2_ adsorption was allowed to proceed until saturation, using the same equilibrium criterion (dm/dt < 0.001 mg min^−1^ for 10 min). After completion of the adsorption step, the sample was heated again to 80°C under dry CO_2_-free air and then exposed to dry air at 80°C for 48 h to assess stability under prolonged oxidative conditions. The sample was subsequently cooled to 30°C, and the wet CO_2_ uptake at 30°C and 70% RH was re-measured following the same protocol described above. This adsorption-exposure-recovery sequence was repeated for three consecutive cycles to evaluate the long-term stability and recyclability of the sorbent under realistic dilute CO_2_ and humid air conditions.

In situ oxidation experiments for the LWT and LWT-bPPI5 samples were conducted using a DRIFTS cell. Prior to oxidation, approximately 50 mg of the resin samples were placed in the cell and activated at 80°C for 2 h under a nitrogen flow of 60 sccm. Following activation, the samples were exposed to air (21% O_2_/N_2_) at 80°C with a flow rate of 60 sccm. The incoming gas was pre-humidified to 75% RH using a bubbler containing saturated sodium chloride solution at room temperature. The experiments were carried out for 24 h, and spectra were collected at fixed time intervals. After the 24 h oxidation study, the samples were collected, activated at 80°C for 2 h under a N_2_ atmosphere, and their adsorption capacities were evaluated using TGA. The measurements were carried out at 30°C under dry conditions for 6 h to determine the adsorption performance.

### Steam stability study

The steam stability test is carried out by placing ∼150 mg of solid in a filter paper cup at a prescribed distance from a round bottom flask with distilled water. The sample is exposed to continuous steam for 72 h under ambient pressure (100°C). The samples were characterized before and after the steam stability test using a Q5000 TA instrument allowing for wet CO_2_ adsorption (Gas 1: N_2_ at 54% RH, Gas 2: 400 ppm CO_2_ balanced with N_2_ at 54% RH). Prior to the measurement, the solid samples (∼20 mg) were activated at 85°C with a ramp rate of 10°C min^−1^ followed by isothermal at 85°C for 1 h under a constant N_2_ flow. The samples were subsequently cooled to 15°C using the same ramp rate and allowed to equilibrate for 15 min. First, water adsorption using Gas 1 was performed until equilibrium was reached (N_2_ at 54% RH and 15°C isothermally). Next, Gas 2 was used to test the CO_2_ capacity under wet conditions, letting the adsorption run until full equilibrium (400 ppm CO_2_ at 54% RH and 15°C isothermally).

## Supplementary Materials

### This PDF file includes:

Supplementary Text Figs. S1 to S34 Tables S1 to S5 References
