# Supplementary material for: In situ azetidine polymerization elevates the performance of commercial polymer resin sorbents in carbon dioxide capture
Source: Sci Adv. 2026 Sep 30;12(40):eaeg5572. doi: 10.1126/sciadv.aeg5572 (PMC13641014; doi:10.1126/sciadv.aeg5572)
Supplement: Supplementary file 1 [file sciadv.aeg5572_sm.pdf]

Science Advances
AAAS

Supplementary Materials for

In situ azetidine polymerization elevates the performance of commercial polymer resin sorbents in carbon dioxide capture

Arkaprabha Giri *et al.*

Corresponding author: Omid Ghaffari Nik, omid@carboncapture.com;
Christopher W. Jones, cjones@chbe.gatech.edu

Sci. Adv. **12**, eaeg5572 (2026)
DOI: 10.1126/sciadv.aeg5572

This PDF file includes:

Supplementary Text
Figs. S1 to S34
Tables S1 to S5
References

Functionalization of amines with porous supports: advantages and limitations: Different amine functionalization strategies for porous supports (general composition: support: 50-85 wt.%; amine: 15-50 wt.%) possess distinct advantages and limitations. In physically impregnated systems, amine leaching during repeated thermal or steam regeneration cycles often leads to gradual loss of sorption capacity. Although the performance can be partially restored by re-impregnation of amines, this adds operational complexity and reduces long-term practicality. In contrast, chemically grafted amines are covalently anchored to the support, significantly suppressing amine leaching during cycling. However, if oxidative degradation of the grafted amines occurs, regeneration of the sorbent becomes challenging because the active functionalities are permanently bound to the support. This issue becomes particularly important for expensive porous supports such as MOFs or COFs, where replacement of degraded sorbents would substantially increase the overall DAC process cost. Therefore, the development of highly oxidation-resistant amines is critical for extending the operational lifetime of chemically grafted sorbents.

The present 'grafting-from' strategy via *in situ* polymerization of azetidine within the pore structure offers several fundamental advantages over direct grafting of preformed polyamines onto porous supports. In conventional post-grafting methods, the relatively large size of preformed polyamines can partially block pore openings during immobilization. Moreover, the amine functionalities of these large polyamines often aggregate or cluster together, resulting in heterogeneous amine distribution and reduced accessibility of active sites. Furthermore, branched polyamines can interact simultaneously with multiple electrophilic surface anchoring sites, such as benzyl chloride, epoxides, carboxylic acids, which can limit the overall grafting density and reduce the fraction of primary amine groups, functionalities that are particularly important for efficient CO_2 capture from ultra-dilute streams (fig. S1a).

By contrast, the *in situ* one-pot polymerization ('grafting-from') approach reported here enables polymer chains to grow directly from surface-bound nucleophilic anchoring sites (i.e., benzyl amine) within the pore network (fig S1b). This strategy provides improved control over chain formation and spatial localization of amine functionalities. Consequently, it promotes a more uniform distribution of amines throughout the pore structure, minimizes pore blockage, and enhances the utilization efficiency of the incorporated amine groups. This approach also helps preserve accessible primary and secondary amine functionalities by reducing crosslinking-induced

loss of active sites, thereby contributing to improved sorption performance. Importantly, polyamines containing propyl spacers exhibit improved oxidative stability compared to their ethyl-spaced analogues. In this context, the *in situ* grafting of bPPI onto low-cost, commercially available resin supports offers a promising strategy for achieving enhanced long-term recyclability and sorbent durability.

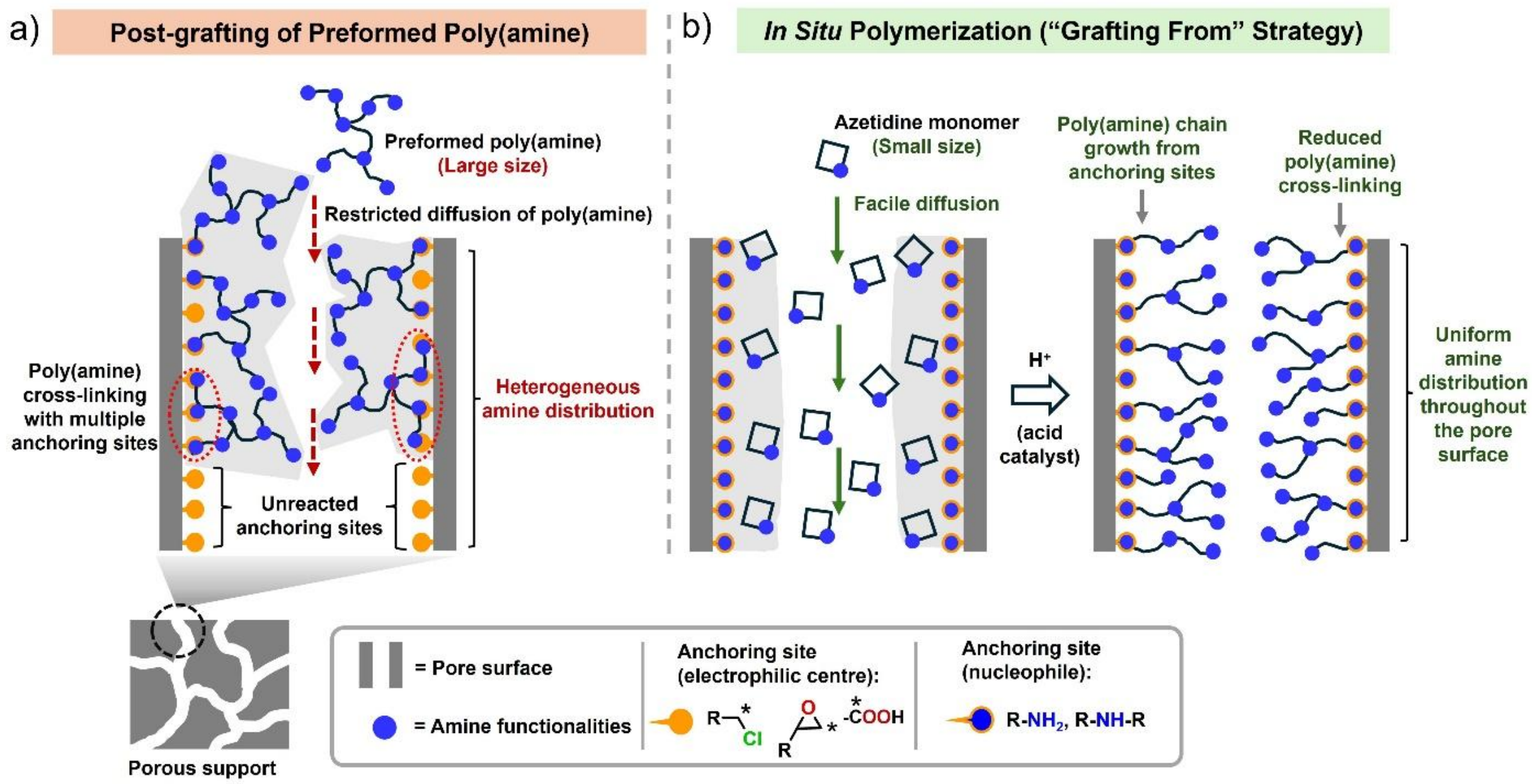

Fig. S1. Schematic depiction of poly(amine)-grafting strategies. (**a**) direct grafting of preformed polyamines onto porous supports, (**b**) 'grafting-from' strategy via *in situ* polymerization of azetidine within the pore structure.

Table S1.

Summary of the elemental (C, H, N) analyses# of various resin samples.

Entry	Resin	C (wt.%)	H (wt.%)	N (wt.%)
1	LWT (duplicate measurements)	79.65	7.83	7.53
		79.1	8.05	8.3
2	LWT-bPPI1	79.45	7.84	8.12
3	LWT-bPPI2	79.75	8.00	8.25
4	LWT-bPPI3	79.13	8.00	8.40
5	LWT-bPPI4	75.05	8.45	9.97
6	LWT-bPPI5 (3 different batches)	74.56	8.82	12.27
		74.32	9.13	12.38
		73.1	9.19	11.9
7	LWT-bPPI6	77.66	8.73	10.78
8	LWT-bPPI7	72.26	8.91	11.65
9	LWT-bPPI8	72.94	8.97	11.60
10	LWT-bPPI9	75.21	8.88	11.38
11	LWT-bPPI5-Recycled	74.12	8.97	12.14
12	Purolite A110	78.48	8.17	9.96
13	Purolite A110-bPPI	72.4	9.2	13.3

#The elemental analyses of the sorbent samples were conducted at Atlantic Microlab, Inc.

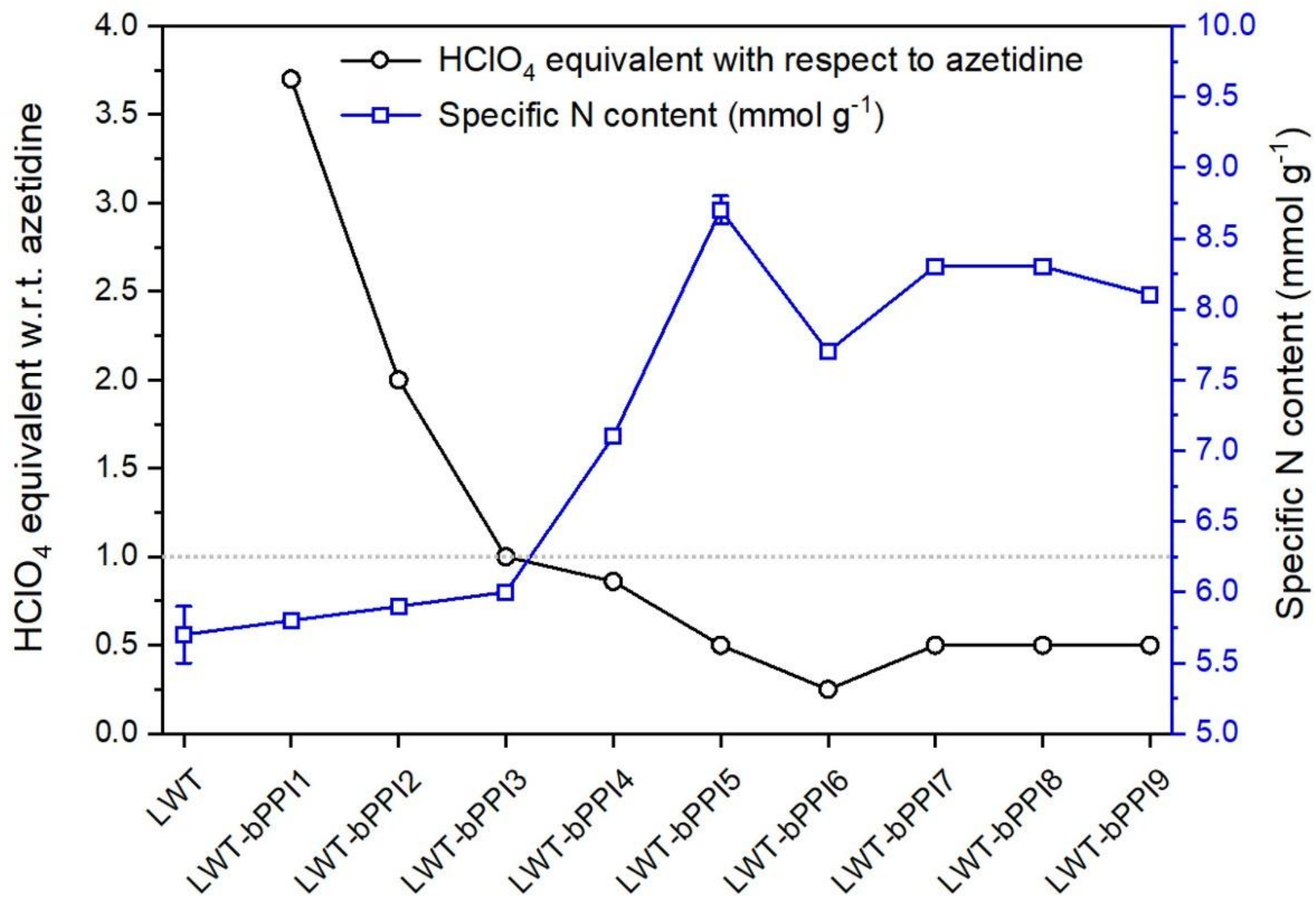

Fig. S2. Influence of $HClO_4$ concentration on the bPPI grafting efficiency. Variation of specific nitrogen content (mmol g^{-1}) of different batches of LWT-bPPIn samples as a function of the $HClO_4$ equivalent (with respect to azetidine) used during the bPPI grafting process.

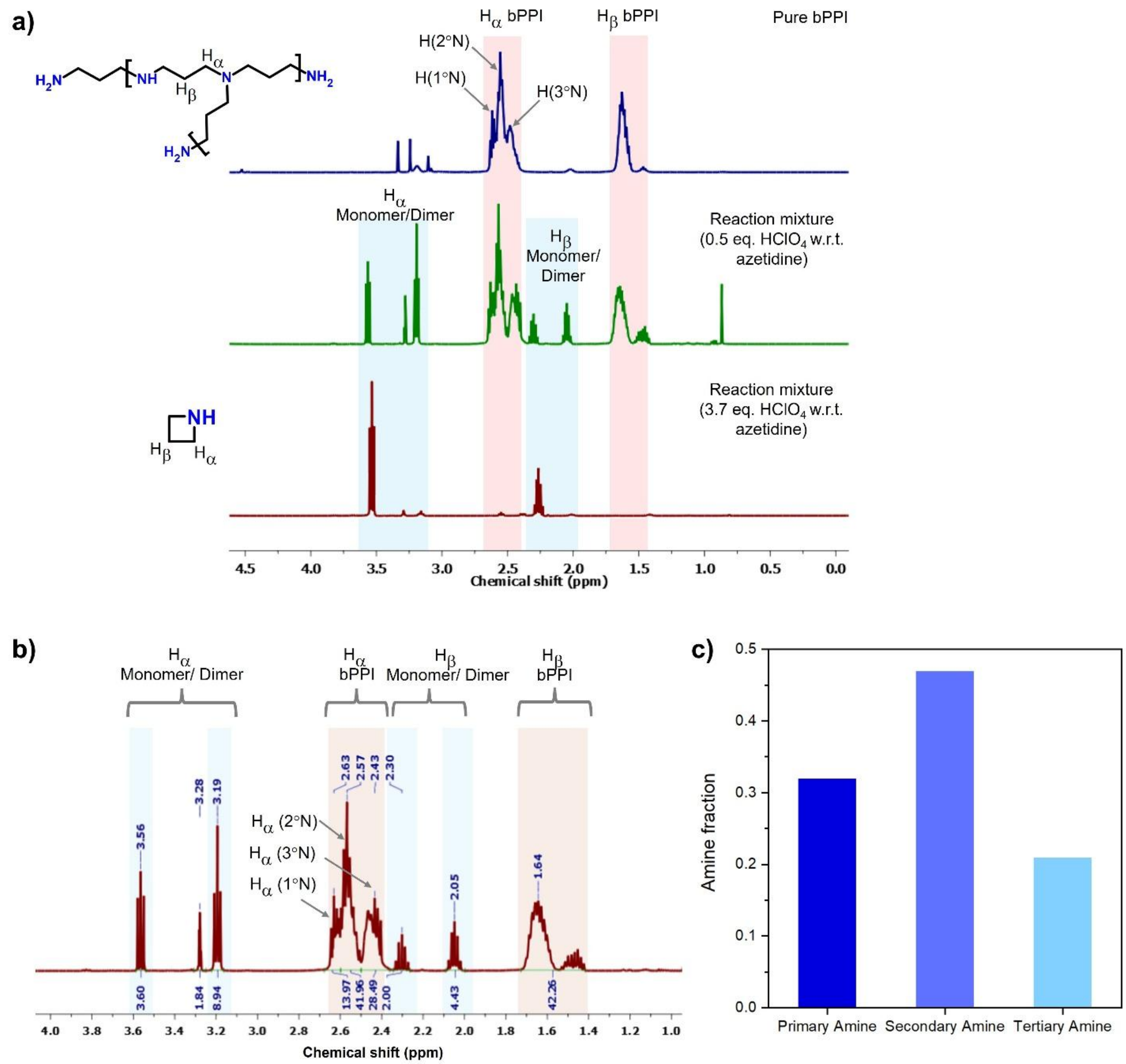

Fig. S3. NMR analyses of the reaction mixture. **(a)** Stacked ^{1}H NMR spectra (in CD_3OD) of the reaction filtrates obtained using 3.7 equiv. of $HClO_4$ with respect to azetidine (maroon) and 0.5 equiv. of $HClO_4$ (green), compared with pure bPPI (blue) (in D_2O). **(b)** ^{1}H NMR (in CD_3OD) spectra of the reaction filtrates obtained using 0.5 equiv. of $HClO_4$ with respect to azetidine. **(c)** Different amine fraction (primary: secondary: tertiary) present in the bPPI.

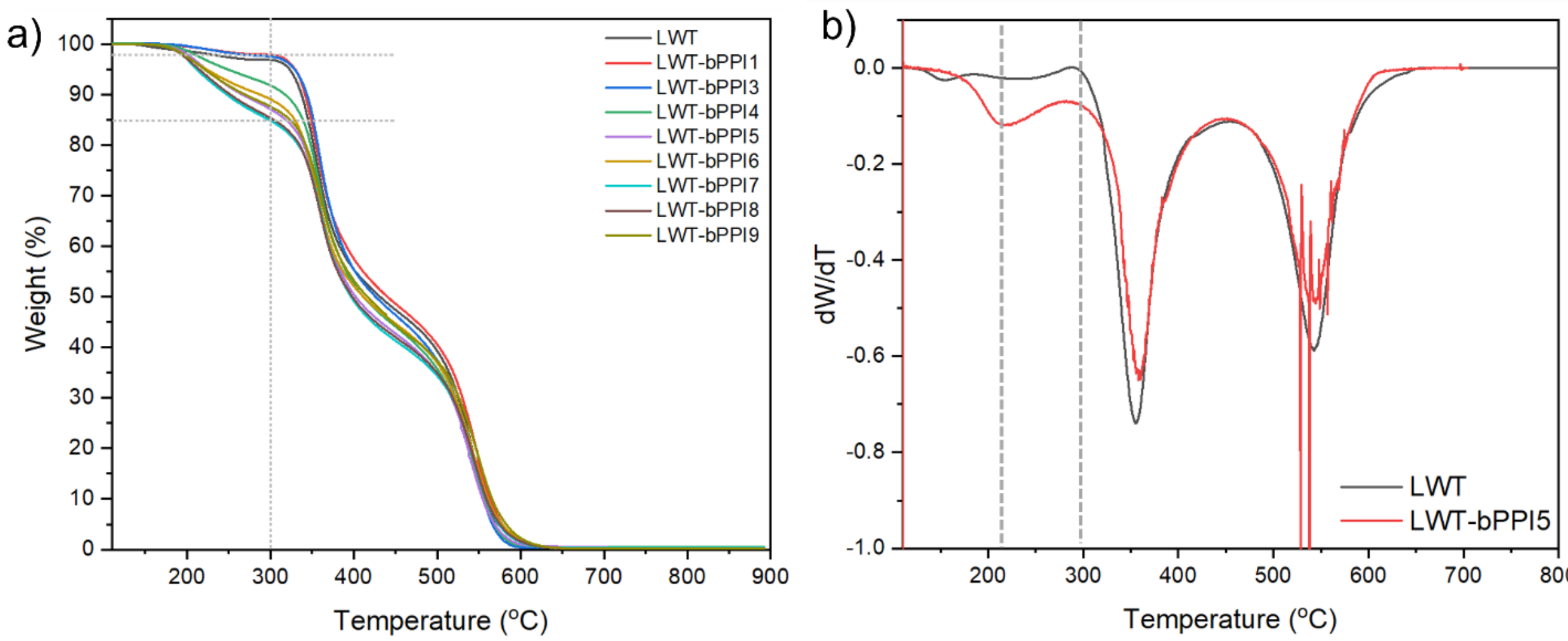

Fig. S4. Thermal stability study of resins. (**a**) Combustion thermogravimetric analysis of LWT and LWT-bPPIn (n=1-9) under air. (**b**) Derivative plot for LWT and LWT-bPPI5.

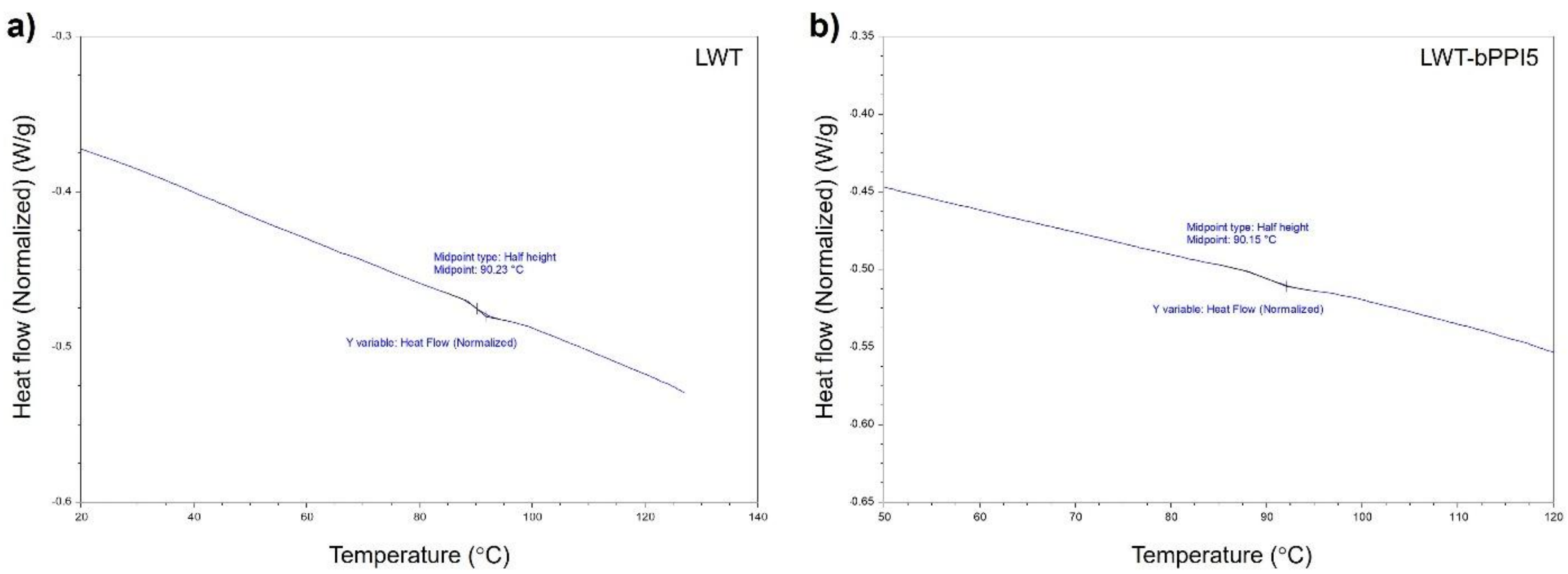

Fig. S5. DSC-TGA analysis of a) LWT and b) LWT-bPPI5.

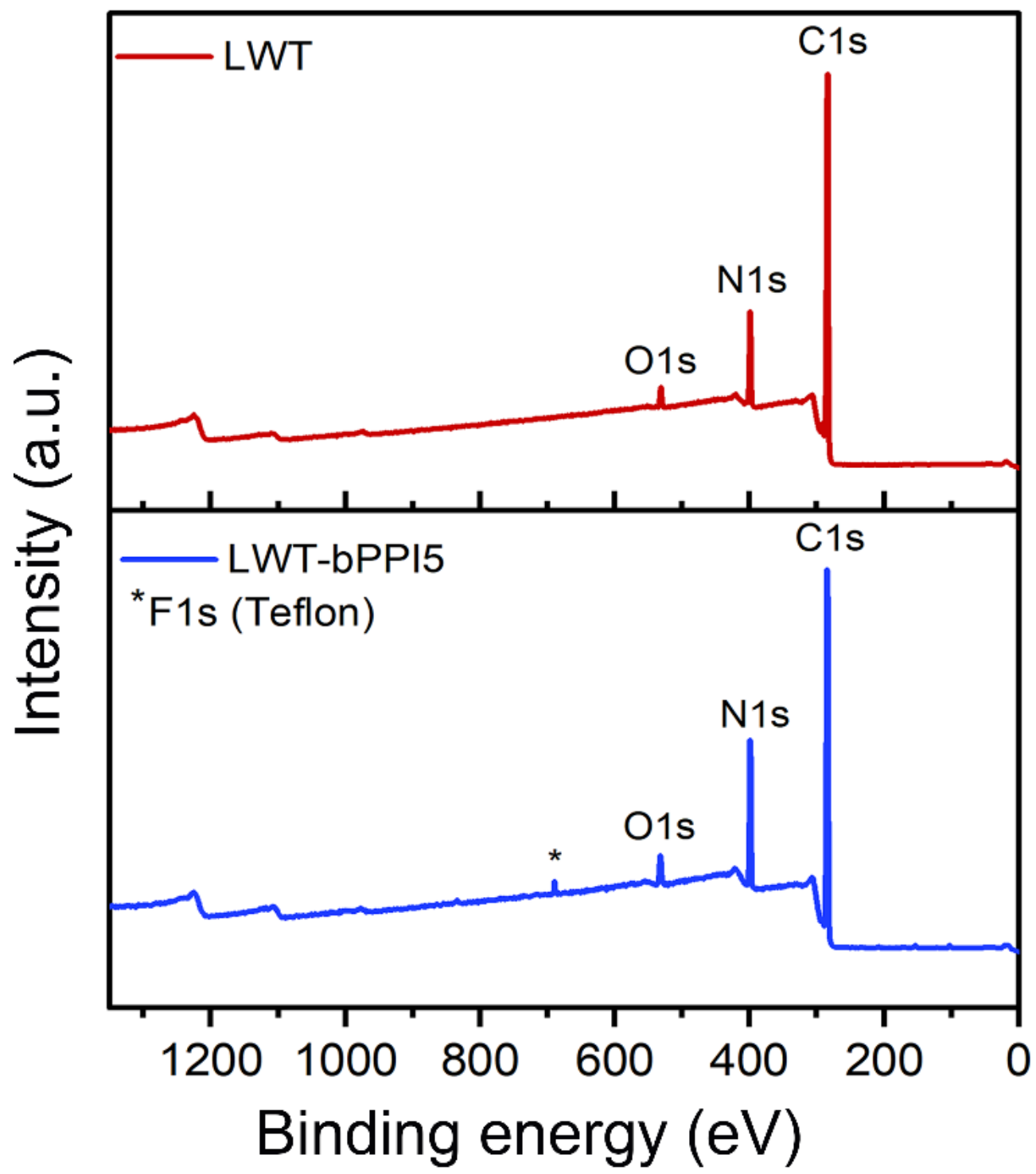

Fig. S6. XPS full spectrum of LWT and LWT-bPPI5.

Table S2.

Comparison of N/C ratio obtained from elemental analysis and XPS analyses of different resin samples.

Resin	Batch	N/C ratio	
		XPS Analysis	CHN Elemental Analysis
LWT-bPPI5	1	0.22	0.14
	2	0.25	0.14
	3	0.26	0.14
LWT	1	0.14	0.09
	2	0.15	0.09

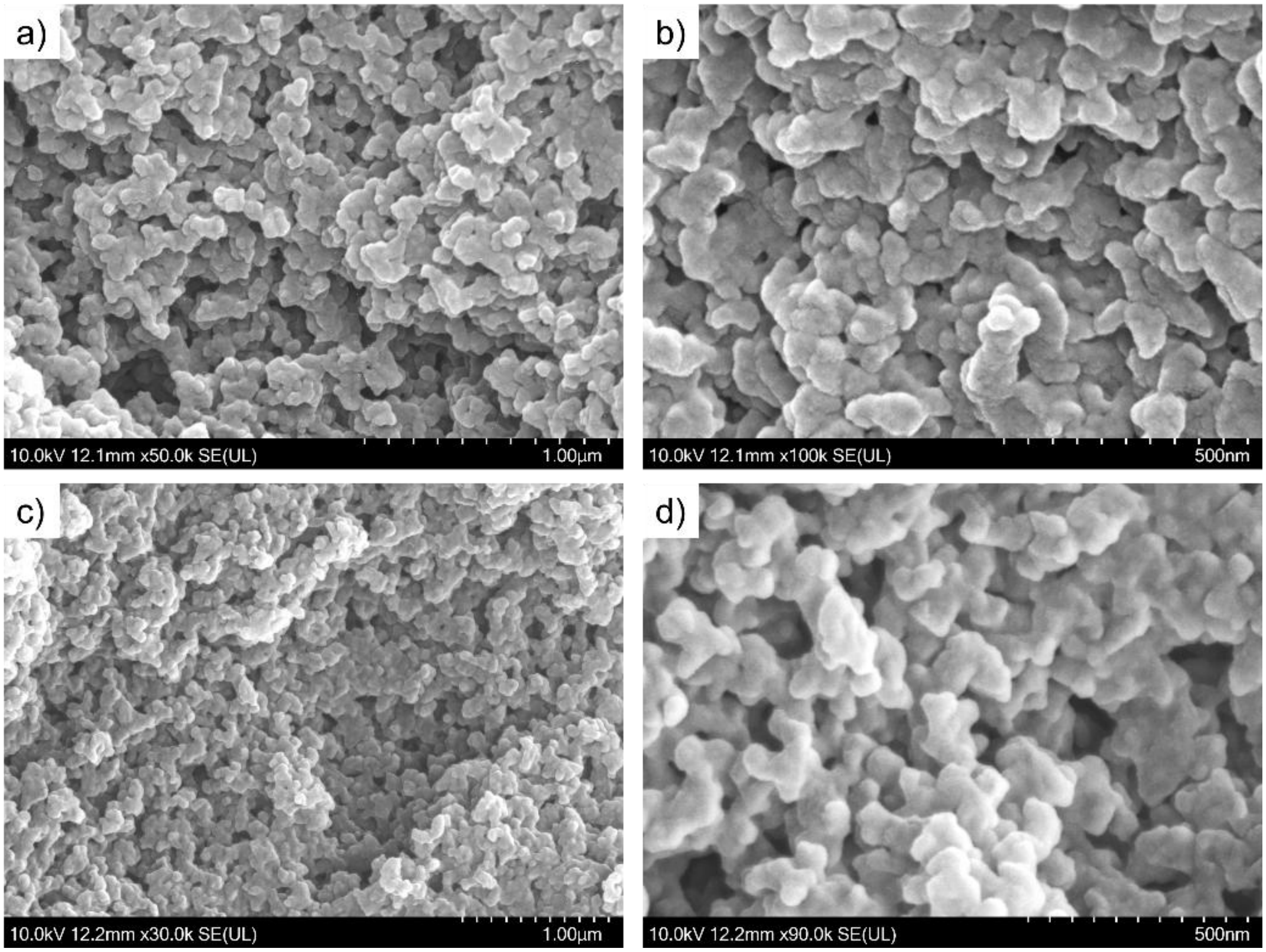

Fig. S7. Morphological analysis of resins. Field emission scanning electron microscopic (FESEM) images of (**a**), (**b**) LWT; (**c**), (**d**) LWT-bPPI5. Images **(a)** and **(d)** are reused in Fig. 3E.

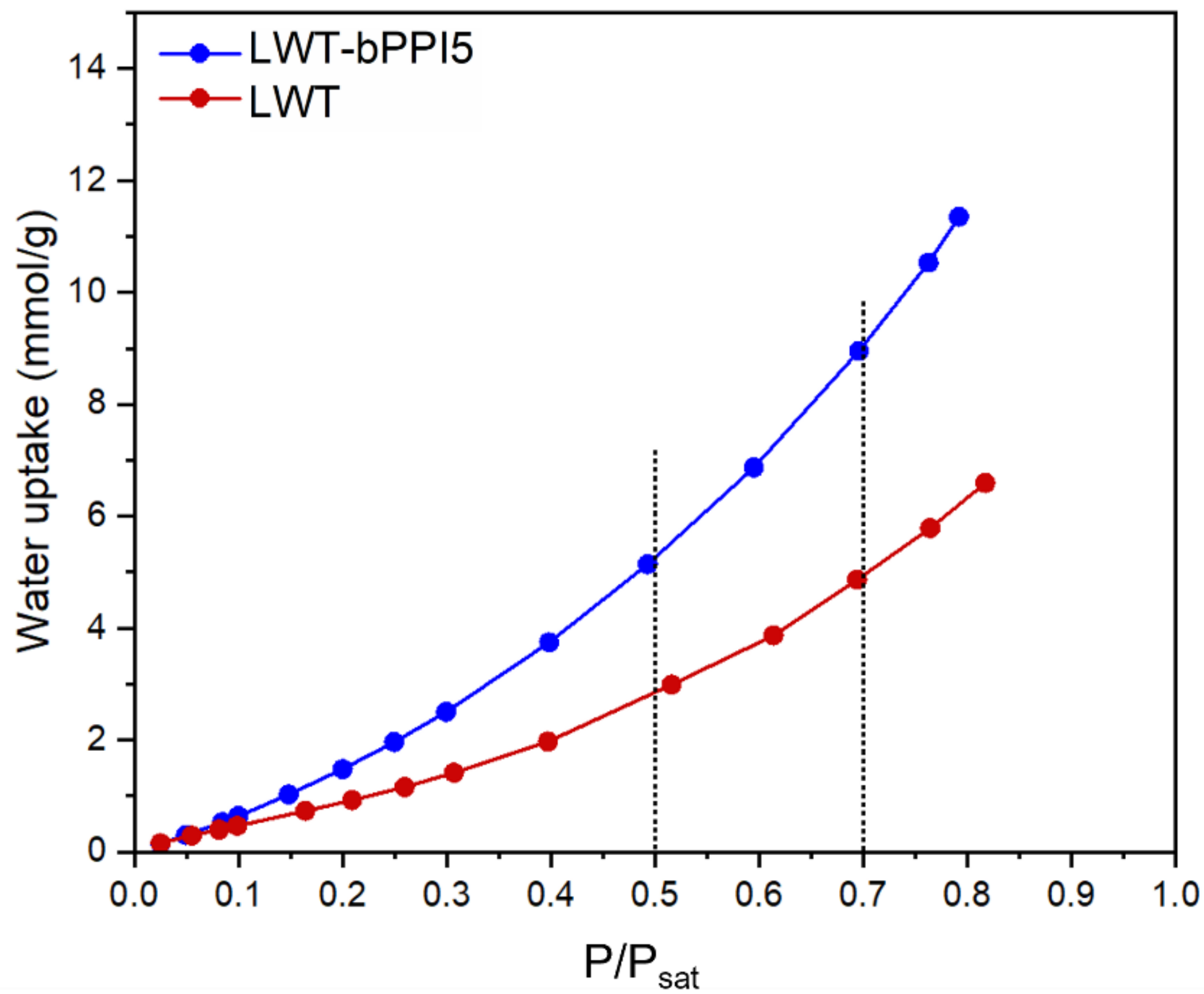

Fig. S8. Water vapor adsorption isotherms of LWT and LWT-bPPI5 at 25°C.

Table S3.

Summary of the water vapor adsorption capacities of different resin batches at 25°C and different relative humidities.

Resin	Batch	Water vapor adsorption capacity at 25°C (mmol g^{-1})	
		@50% RH	@70% RH
LWT-bPPI5	1	5.85	10.2
	2	5.1	8.95
	3	4.5	7.8
LWT	1	5.7	3.5
	2	4.9	3.0

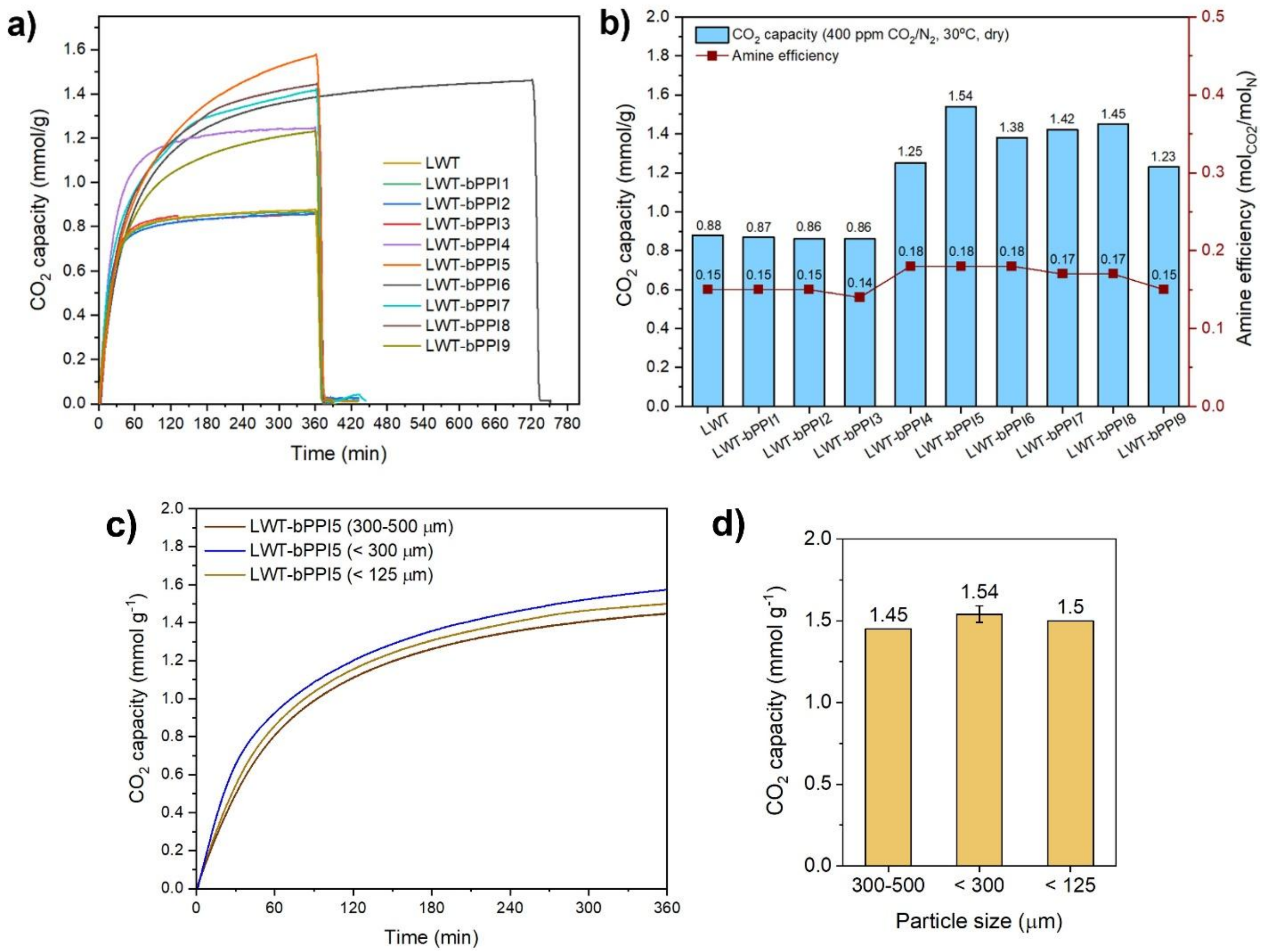

Fig. S9. CO_2 adsorption of different batches of bPPI-grafted resins under dry DAC conditions. (**a**) Thermogravimetric analysis of CO_2 adsorption on LWT and LWT-bPPIn (n=1-9), under 400 ppm CO_2/N_2 at 30°C and 0% relative humidity. (**b**) The corresponding uptake capacity and the amine efficiency in bar plot. (**c**) Thermogravimetric analysis of CO_2 adsorption on LWT-bPPI5 with different particle size (<125 µm, <300 µm, and 300-500 µm), under 400 ppm CO_2/N_2 at 30°C and 0% relative humidity. LWT-bPPI5 samples were synthesized using ground pristine LWT with different particle size ranges, obtained by sieving the ground material through meshes of different sizes, under identical reaction conditions. (**d**) The corresponding uptake capacity of LWT-bPPI5 samples with different particle sizes.

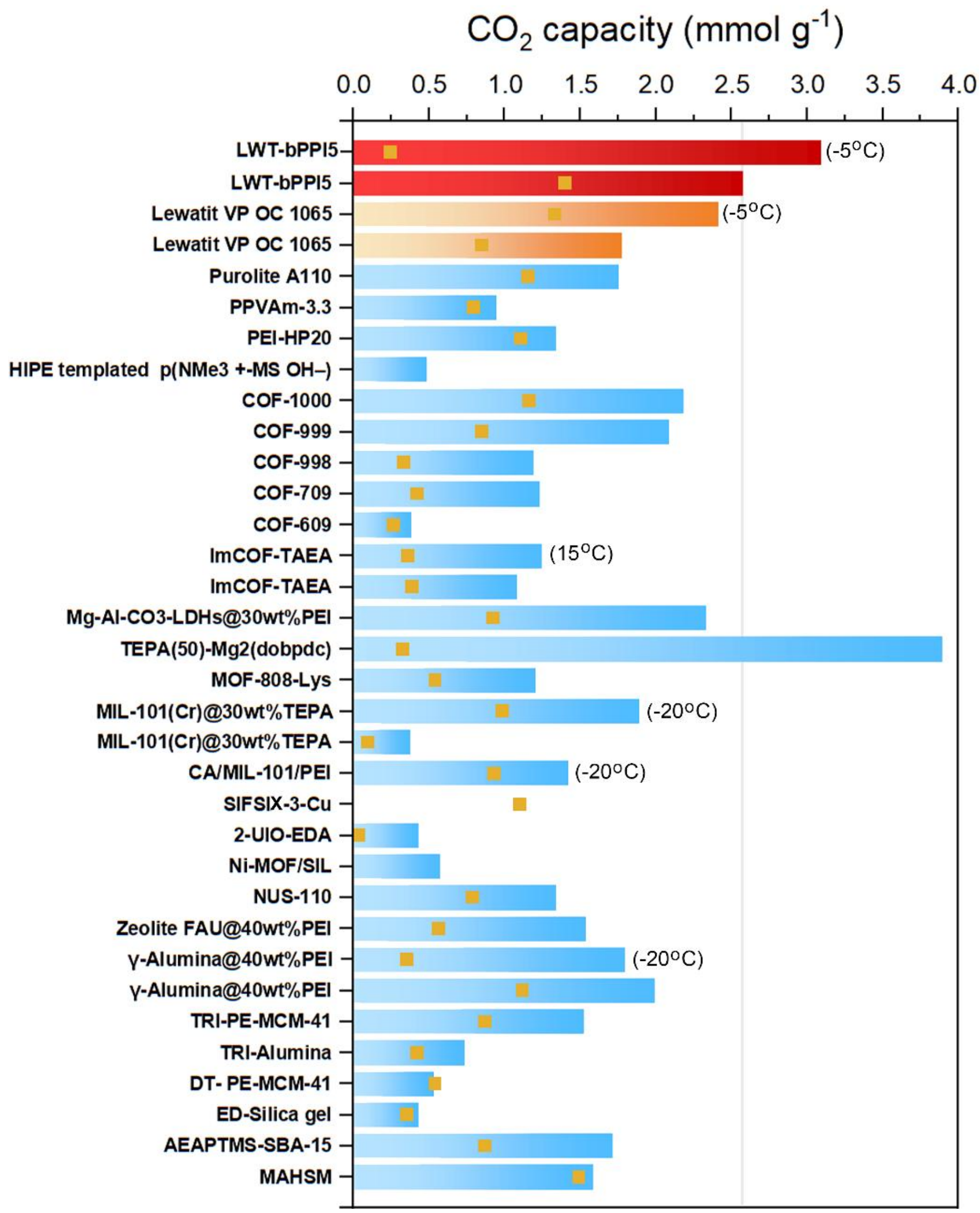

Fig. S10. Comparative CO_2 capture performance of LWT and LWT-bPPI5 alongside benchmark sorbents under direct air capture (DAC) conditions. Comparative CO_2 uptake capacities of LWT-bPPI5 evaluated in this work alongside reported benchmark amine-functionalized solid sorbents under dry (yellow boxes) and humid (bars) DAC-relevant conditions (400 ppm CO_2), including amine-impregnated and grafted silica, alumina, zeolite, mixed metal oxides, metal-organic framework (MOF), covalent organic framework (COF), and organic resin support. All capacities are reported as pseudo-equilibrium or equilibrium CO_2 uptake in mmol g^{-1} at 25°C unless otherwise specified in parentheses. The detailed capacity values, measurement conditions, and corresponding references are provided in Table S5.

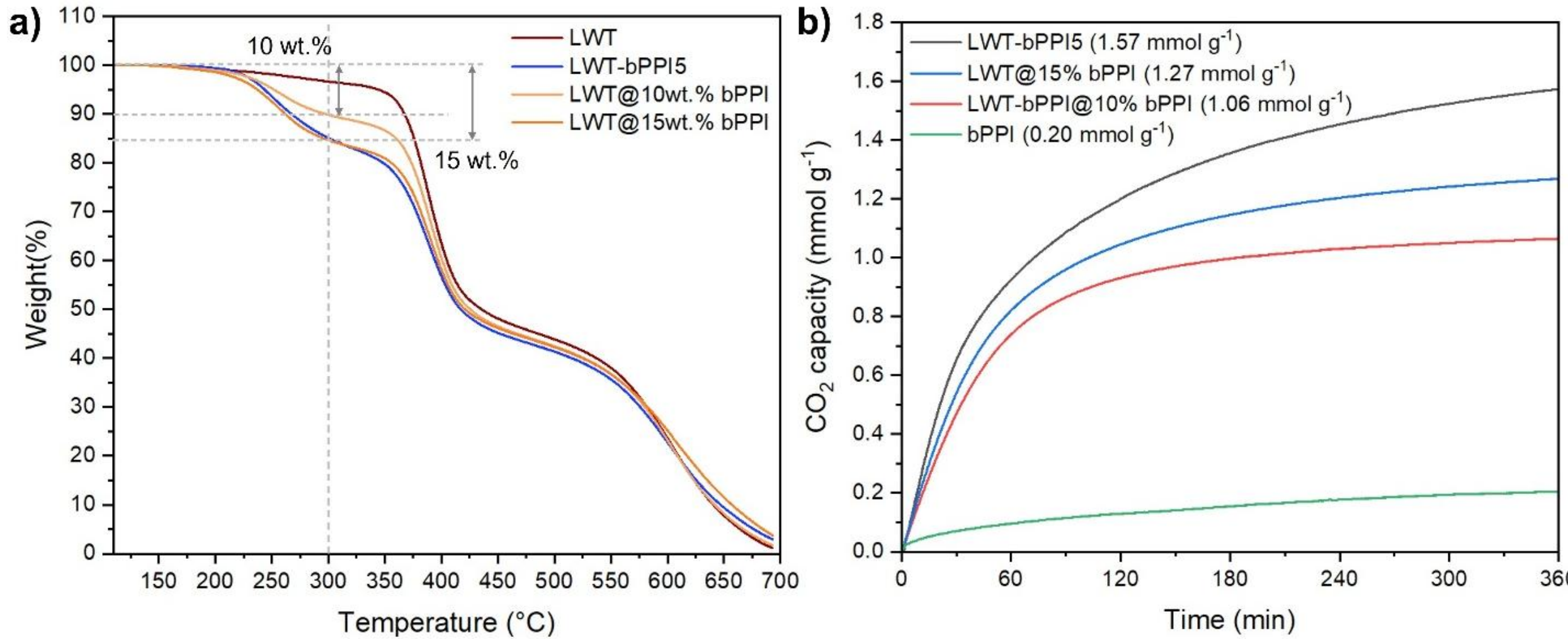

Fig. S11. Comparative thermogravimetric analysis of resins. (a) Pyrolysis thermogravimetric analysis of LWT, LWT-bPPI5, and bPPI impregnated LWT (10 and 15 wt.%) samples under air. **(b)** Thermogravimetric analysis of CO_2 adsorption on LWT-bPPI5, physically impregnated bPPI on LWT resin (LWT@10%bPPI, LWT@15%bPPI), and pristine bPPI polymer under 400 ppm CO_2/N_2 at 30°C and 0% relative humidity.

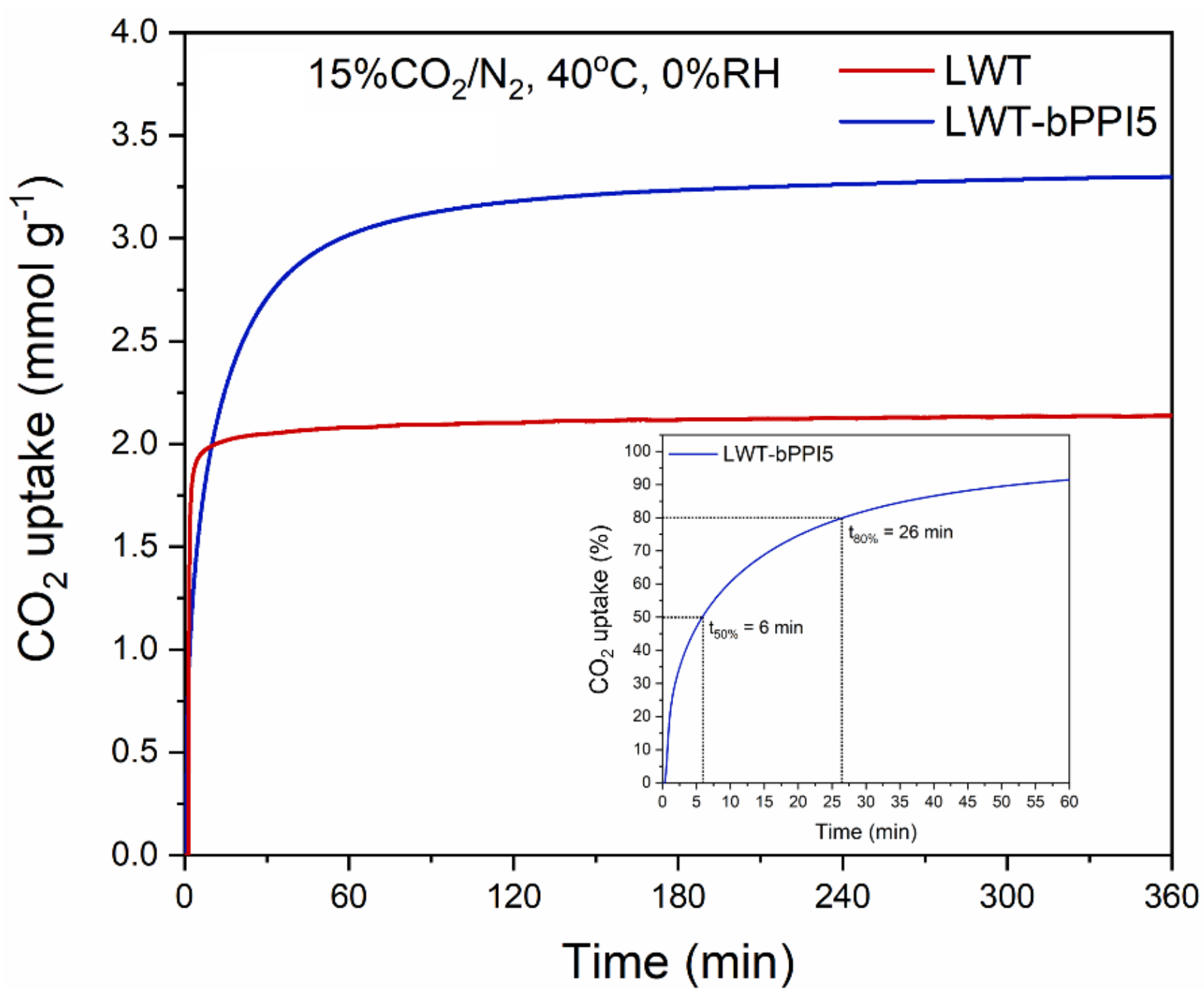

Fig. S12. CO_2 adsorption under flue gas conditions. Thermogravimetric analysis of CO_2 adsorption on LWT and LWT-bPPI5 under simulated flue gas conditions (i.e., 15% ppm CO_2/N_2 at 40°C and 0% RH). $t_{50\%}$ and $t_{80\%}$ indicate the time (in min) required to reach 50% and 80% of the overall capacity.

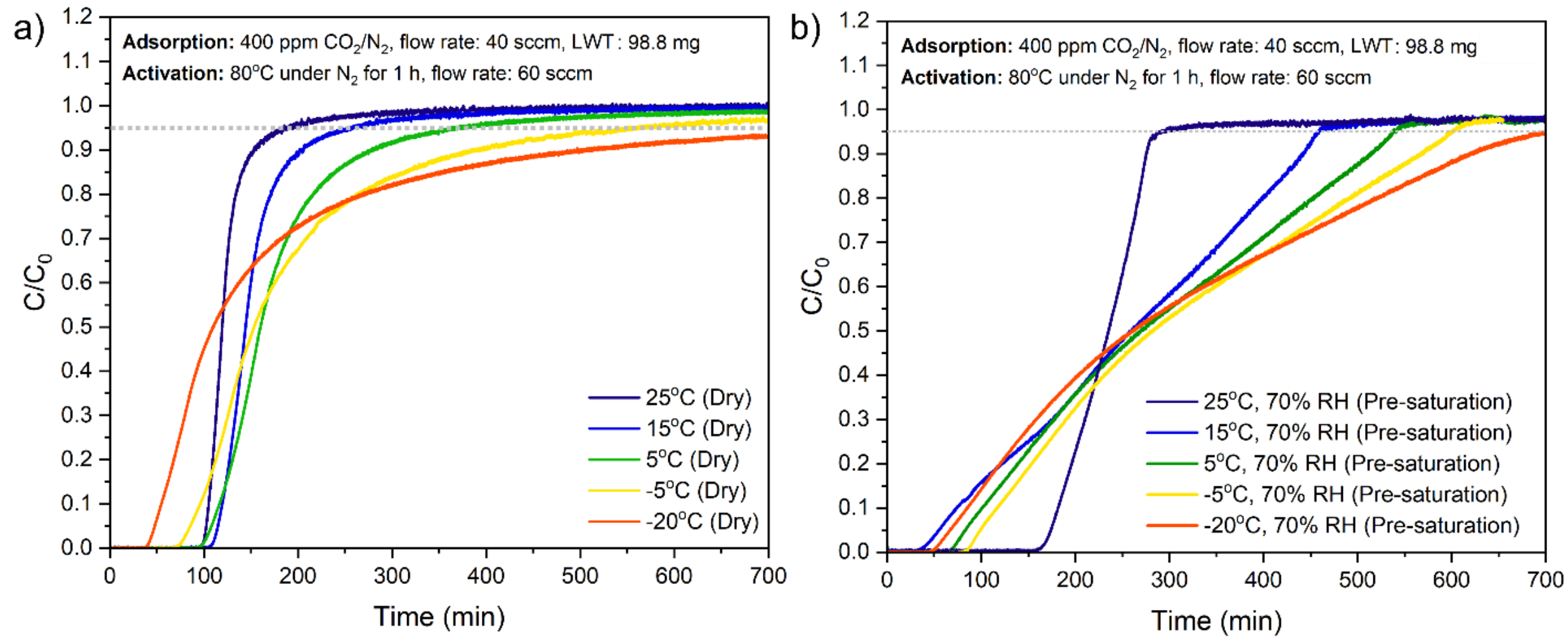

Fig. S13. CO_2 dynamic breakthrough analysis of LWT. CO_2 dynamic breakthrough curves for LWT under 400 ppm of CO_2/N_2 at different temperatures with (**a**) dry (**b**) humid (70% RH) pre-saturation conditions. In case of the presaturation humid experiments, LWT was pre-humidified with 70% RH/N_2 (flow rate: 40 sccm), at the respective temperatures before switching to humid 400 ppm CO_2/N_2 adsorption (flow rate: 40 sccm).

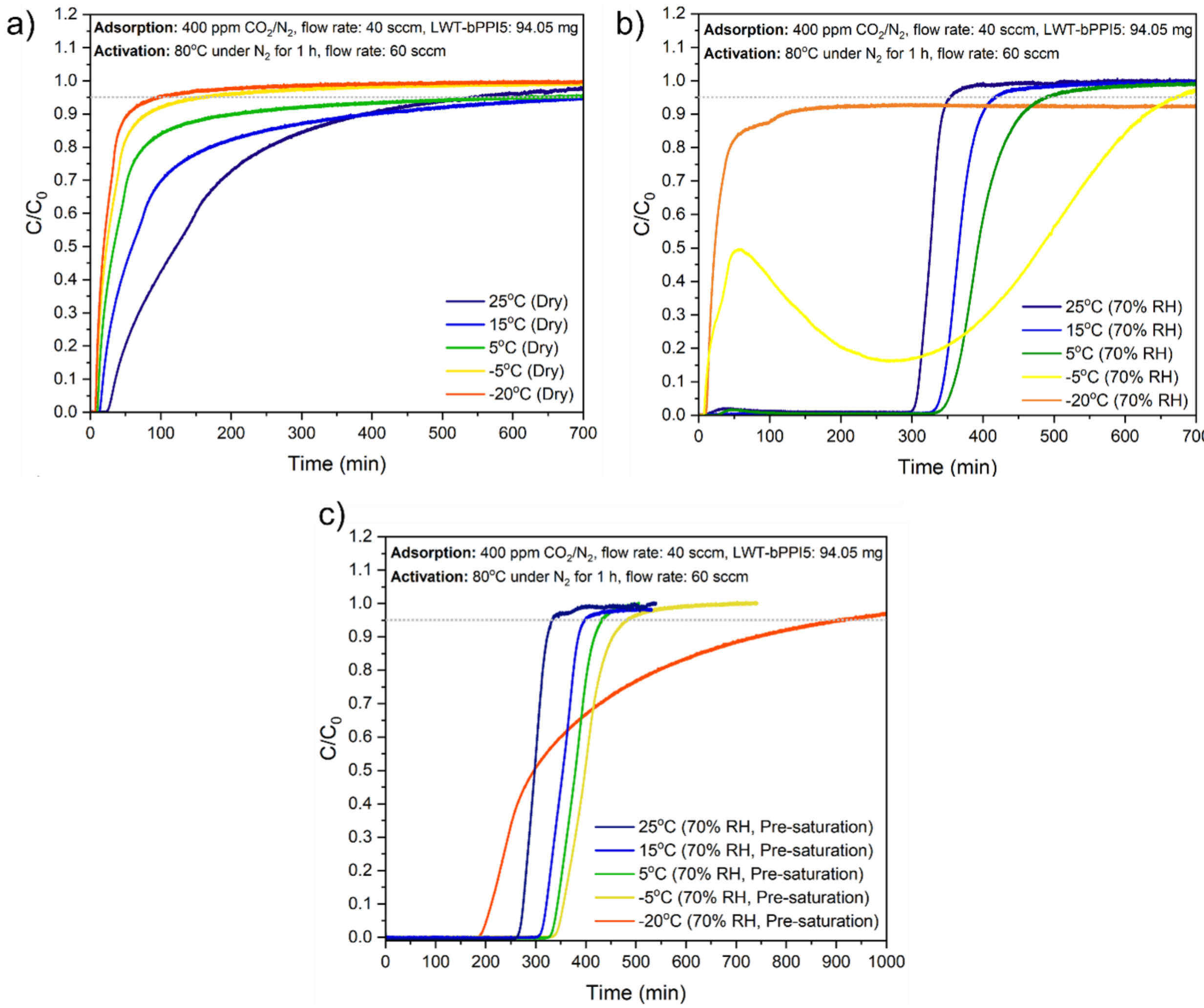

Fig. S14. CO_2 dynamic breakthrough analysis of LWT-bPPI5. CO_2 dynamic breakthrough curves for LWT-bPPI5 under 400 ppm of CO_2/N_2 at different temperatures with (**a**) dry (**b**) humid (70% RH) co-adsorption, (**c**) humid (70% RH) pre-saturation conditions. In case of the presaturation humid experiments, LWT-bPPI5 was pre-humidified with 70% RH/N_2 (flow rate: 40 sccm), at the respective temperatures before switching to humid 400 ppm CO_2/N_2 adsorption (flow rate: 40 sccm).

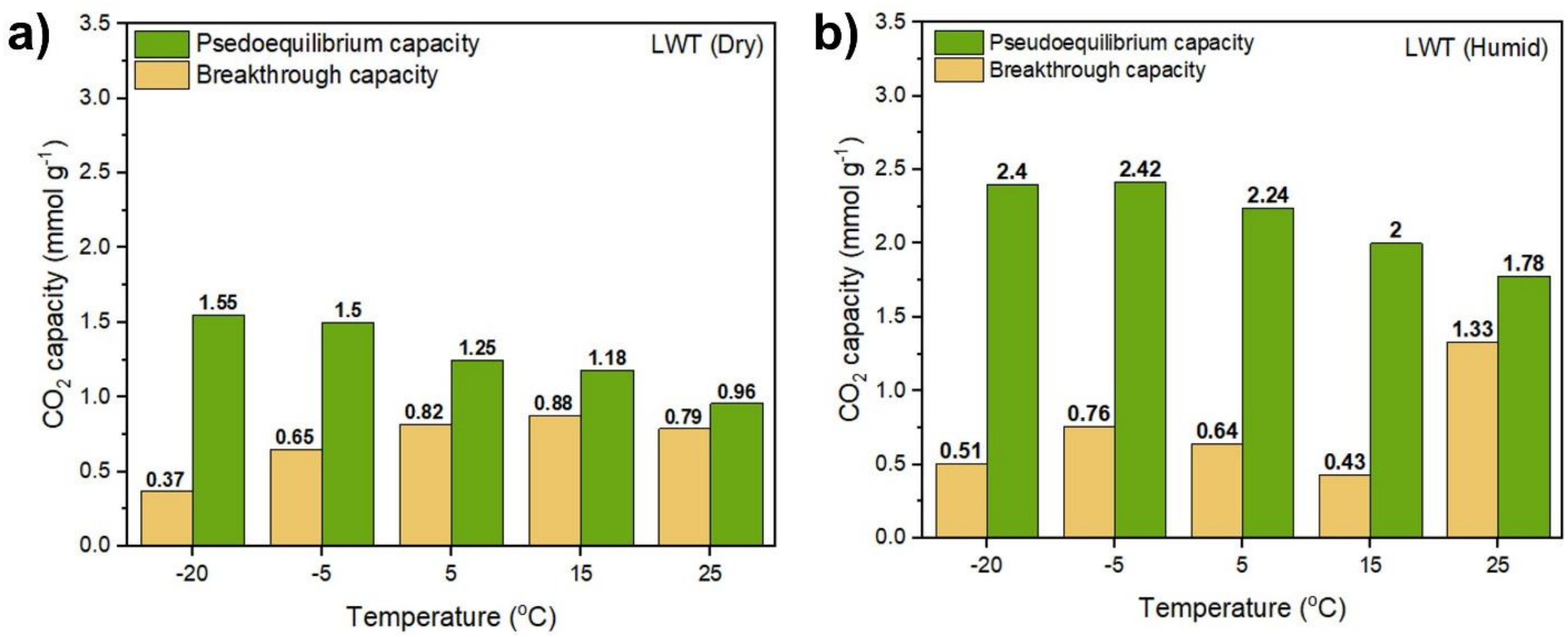

Fig S15. Comparative breakthrough capacities and pseudo-equilibrium capacities of LWT dry and humid DAC conditions. Breakthrough analysis of LWT at different temperatures under **(a)** dry (0% RH) and **(b)** humid (70% RH) DAC conditions, showing pseudo-equilibrium capacity (green bar) and breakthrough capacity (yellow bar). Breakthrough capacities and pseudo-equilibrium capacities were calculated at the times when the CO_2 concentration reached 5% and 95% of C_0 (i.e., 400 ppm CO_2/N_2), respectively.

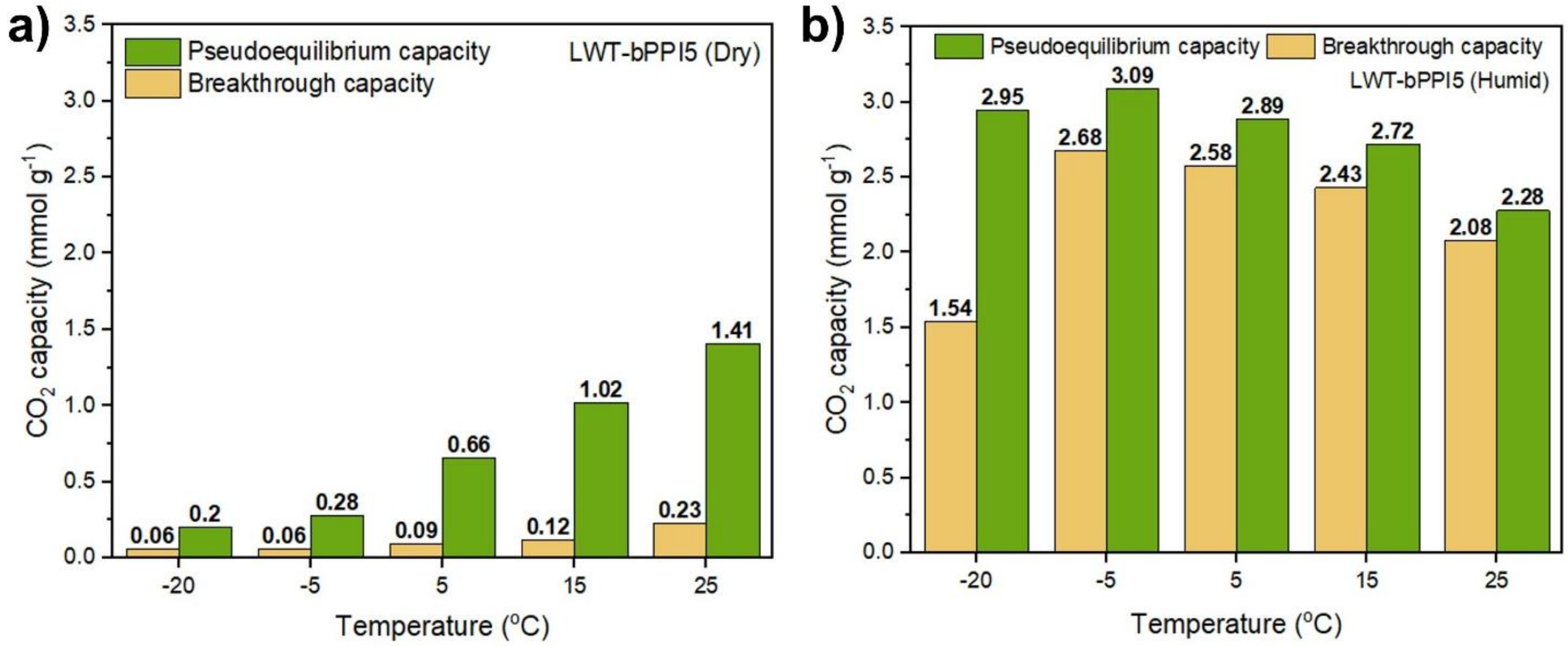

Fig S16. Comparative breakthrough capacities and pseudo-equilibrium capacities of LWT-bPPI5 dry and humid DAC conditions. Breakthrough analysis of LWT-bPPI5 at different temperatures under **(a)** dry (0% RH) and **(b)** humid (70% RH) DAC conditions, showing pseudo-equilibrium capacity (green bar) and breakthrough capacity (yellow bar). Breakthrough capacities and pseudo-equilibrium capacities were calculated at the times when the CO_2 concentration reached 5% and 95% of C_0 (i.e., 400 ppm CO_2/N_2), respectively.

Table S4. Summary of breakthrough analysis parameters for LWT and LWT-bPPI5 under different temperature and humidity (presaturation) conditions [*Mass transfer zone time referred here as the time length between the breakthrough (C/C_0: 5%) and pseudoequilibrium (C/C_0: 95 %) time points] (*72*).

	Temp. (°C)	Pseudoequilibrium capacity (mmol g^{-1})		Breakthrough capacity (mmol g^{-1})		Breakthrough time (min)		Mass transfer zone time (min)*	
		**Dry**	**Humid**	**Dry**	**Humid**	**Dry**	**Humid**	**Dry**	**Humid**
**LWT**	**25**	0.96	1.78	0.79	1.33	103	175	80	120
	**15**	1.18	2.00	0.88	0.43	115	56	150	404
	**5**	1.25	2.24	0.82	0.64	108	84	268	456
	**-5**	1.5	2.42	0.65	0.76	85	100	469	500
	**-20**	1.55	2.4	0.37	0.51	48	67	672	648
**LWT-bPPI5**	**25**	1.41	2.28	0.23	2.08	31	271	514	60
	**15**	1.02	2.72	0.12	2.43	16	316	659	81
	**5**	0.66	2.89	0.09	2.58	11	338	589	94
	**-5**	0.28	3.09	0.06	2.68	8	349	156	129
	**-20**	0.2	2.95	0.06	1.54	8	202	86	698

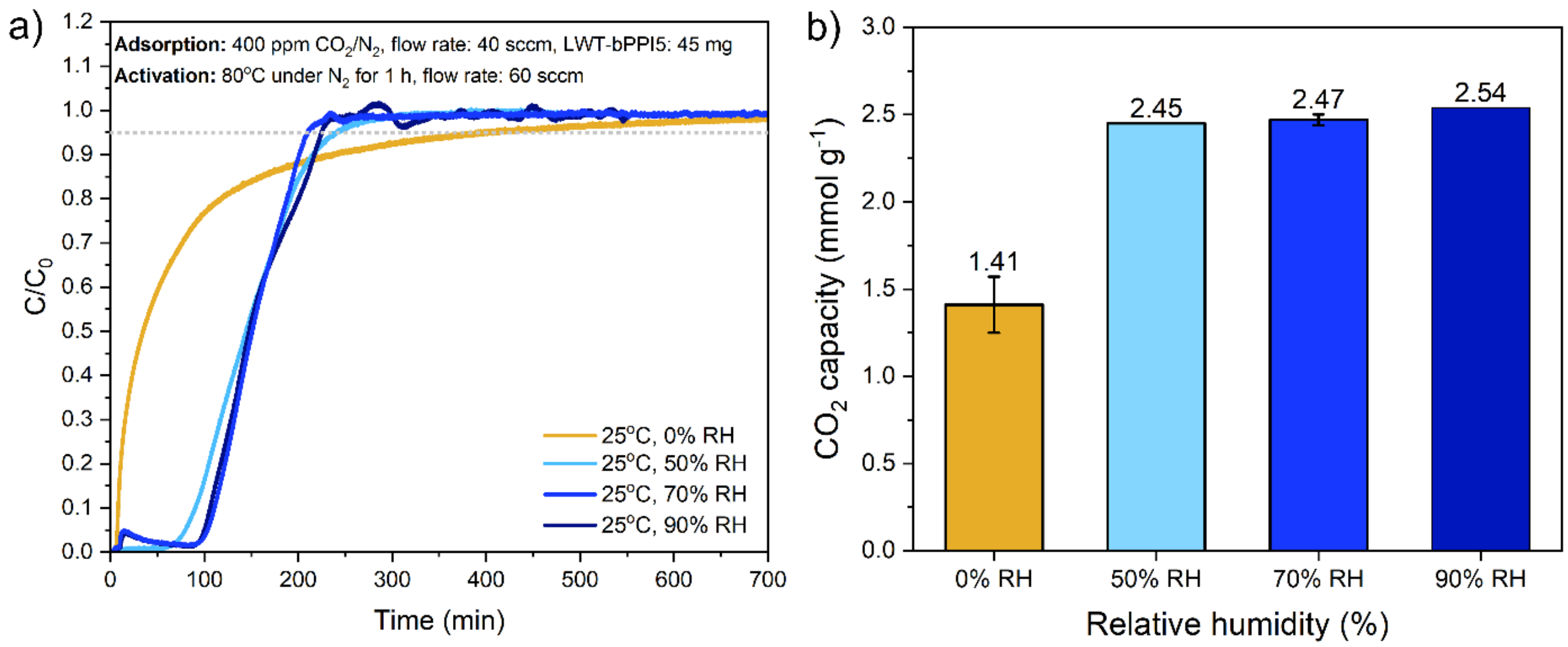

Fig. S17. Effect of humidity on CO_2 sorption. (**a**) CO_2 dynamic breakthrough curves and (**b**) estimated CO_2 uptake (pseudo-equilibrium capacity, $C/C_0 = 0.95$) under 400 ppm of CO_2/N_2 with 0%, 50%, 70%, and 90% relative humidity (RH, co-adsorption method) at 25°C for LWT-bPPI5.

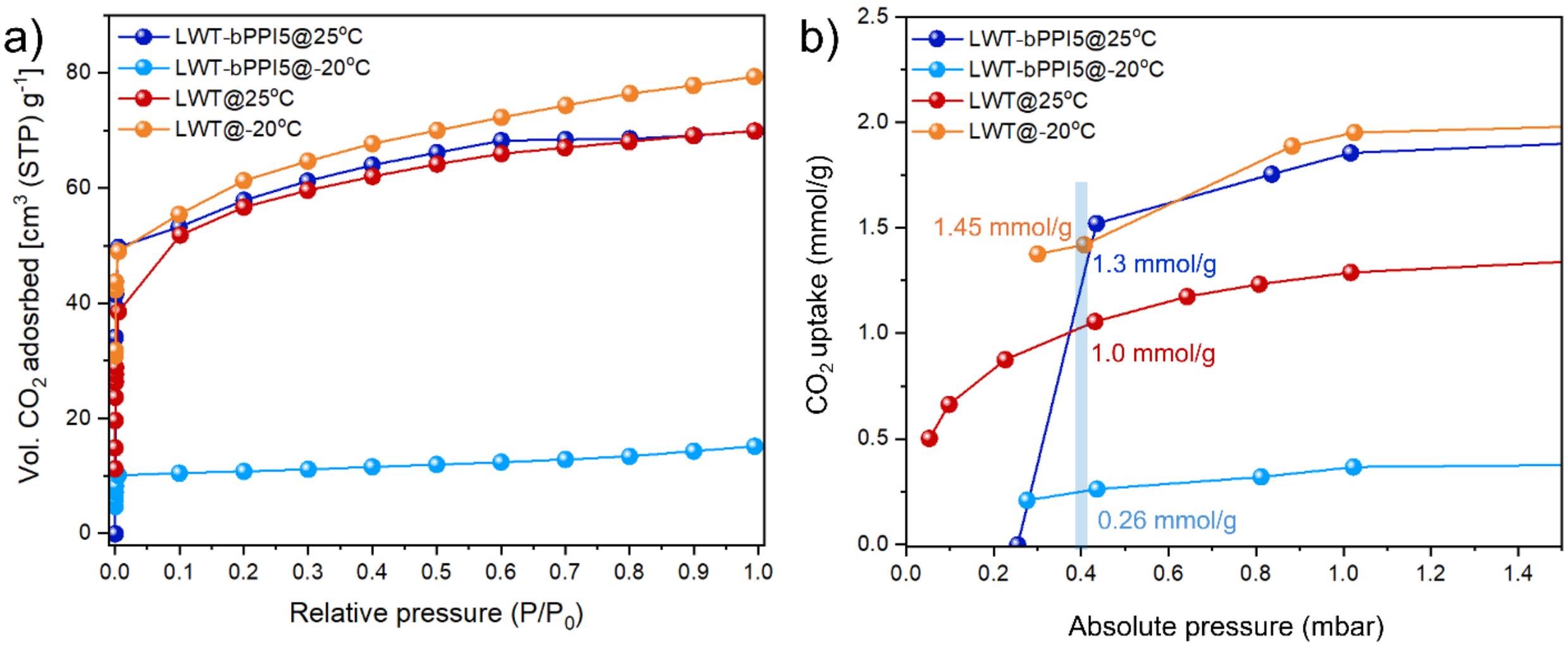

Fig. S18. Single component CO_2 sorption isotherm analyses at different temperatures. (**a**) Single-component CO_2 and N_2 sorption isotherms of LWT and LWT-bPPI5 at ambient (25°C) and sub-ambient (-20°C) temperature. (**b**) Zoomed-in view of the isotherms highlighting the equilibrium uptake by LWT and LWT-bPPI5 at the approximate ambient air CO_2 pressure (~0.4 mbar).

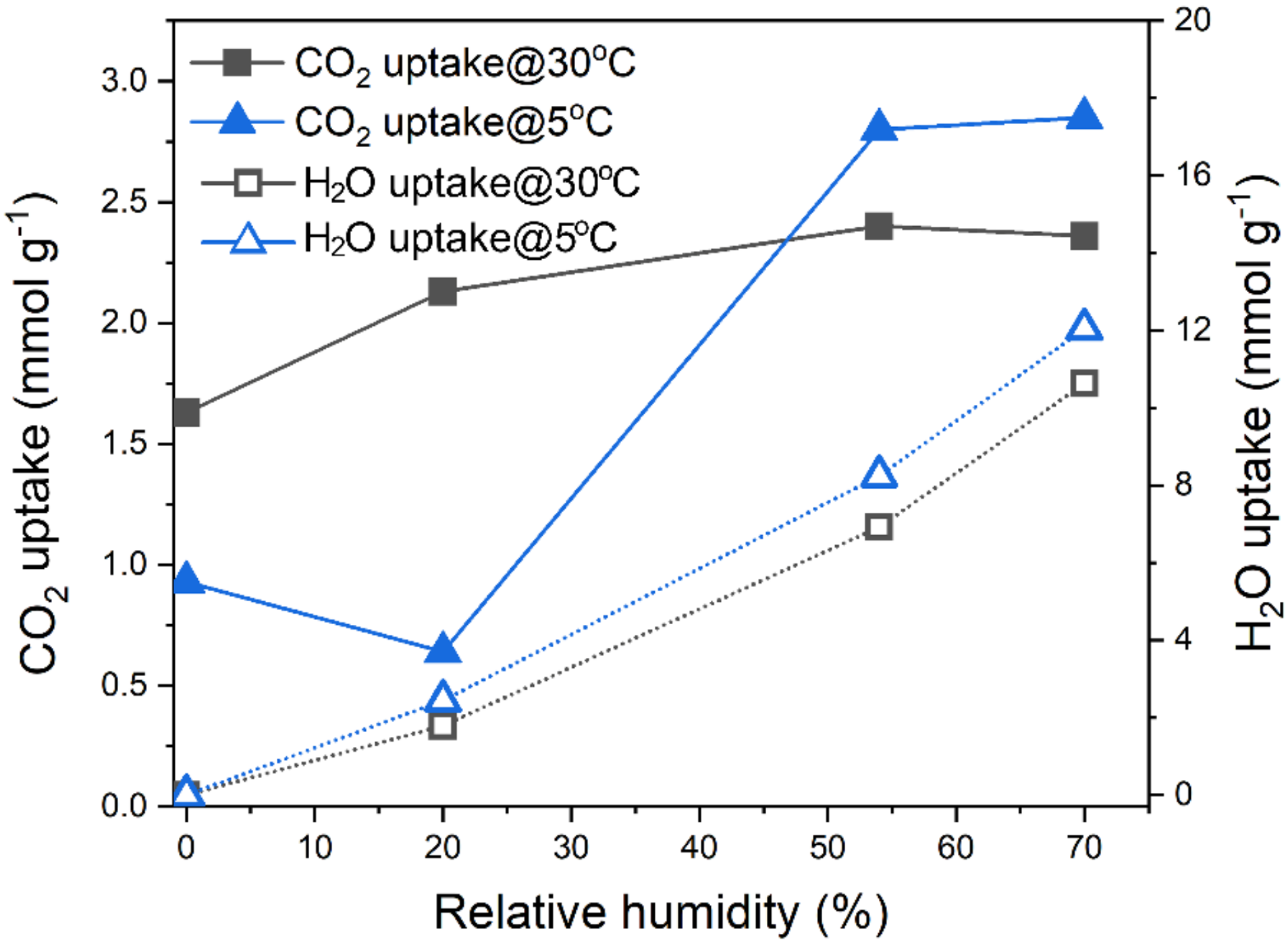

Fig. S19. Dynamic vapor sorption (DVS) CO_2-H_2O equilibrium data analysis of LWT-bPPI5 at different temperatures (30°C, 5°C). All the CO_2 data was collected at 400 ppm CO_2 concentration, 100°C regeneration under N_2.

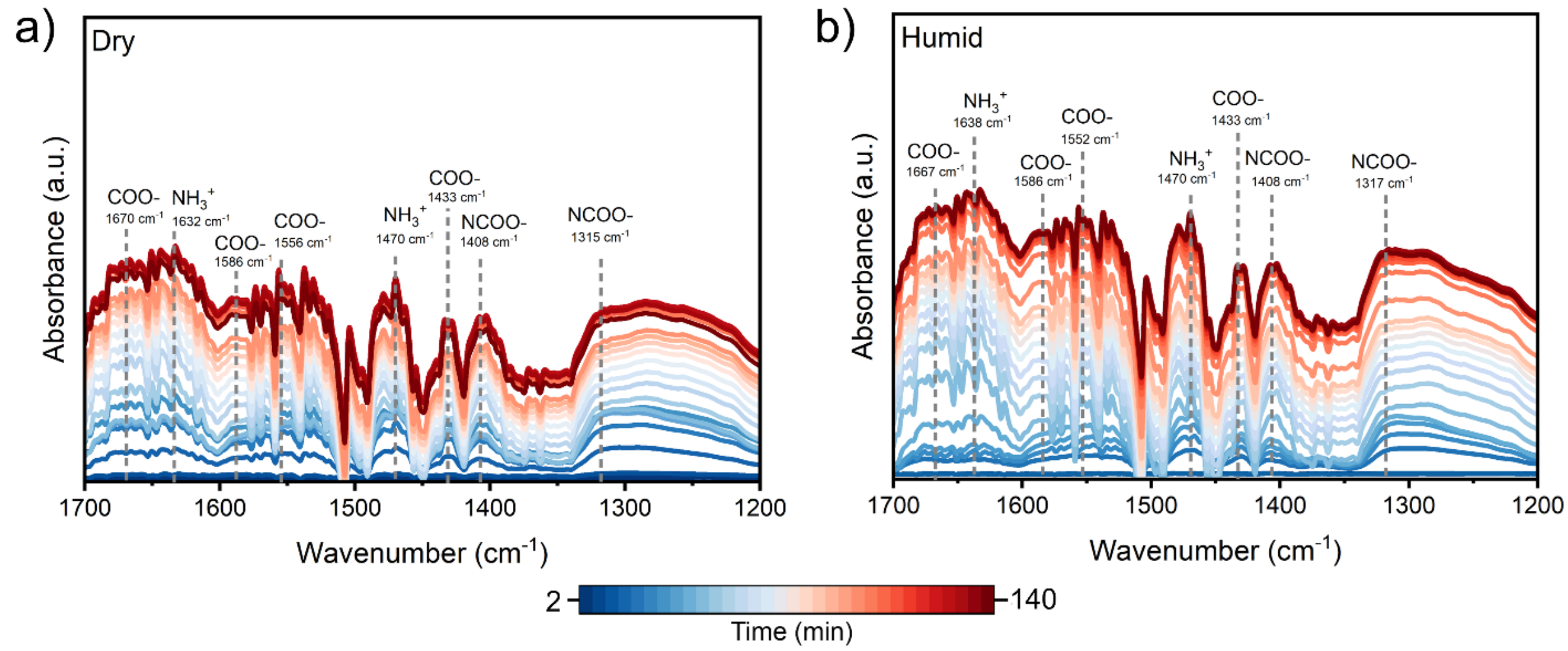

Fig. S20. DRIFTS analysis of LWT. *In situ* DRIFT spectra of LWT as a function of adsorption time at 25°C under **(a)** dry and **(b)** humid conditions (75% RH). Adsorption conditions: 400 ppm CO_2/N_2; flow rate: 40 sccm; activation, 80°C under 60 sccm N_2 for 2 h.

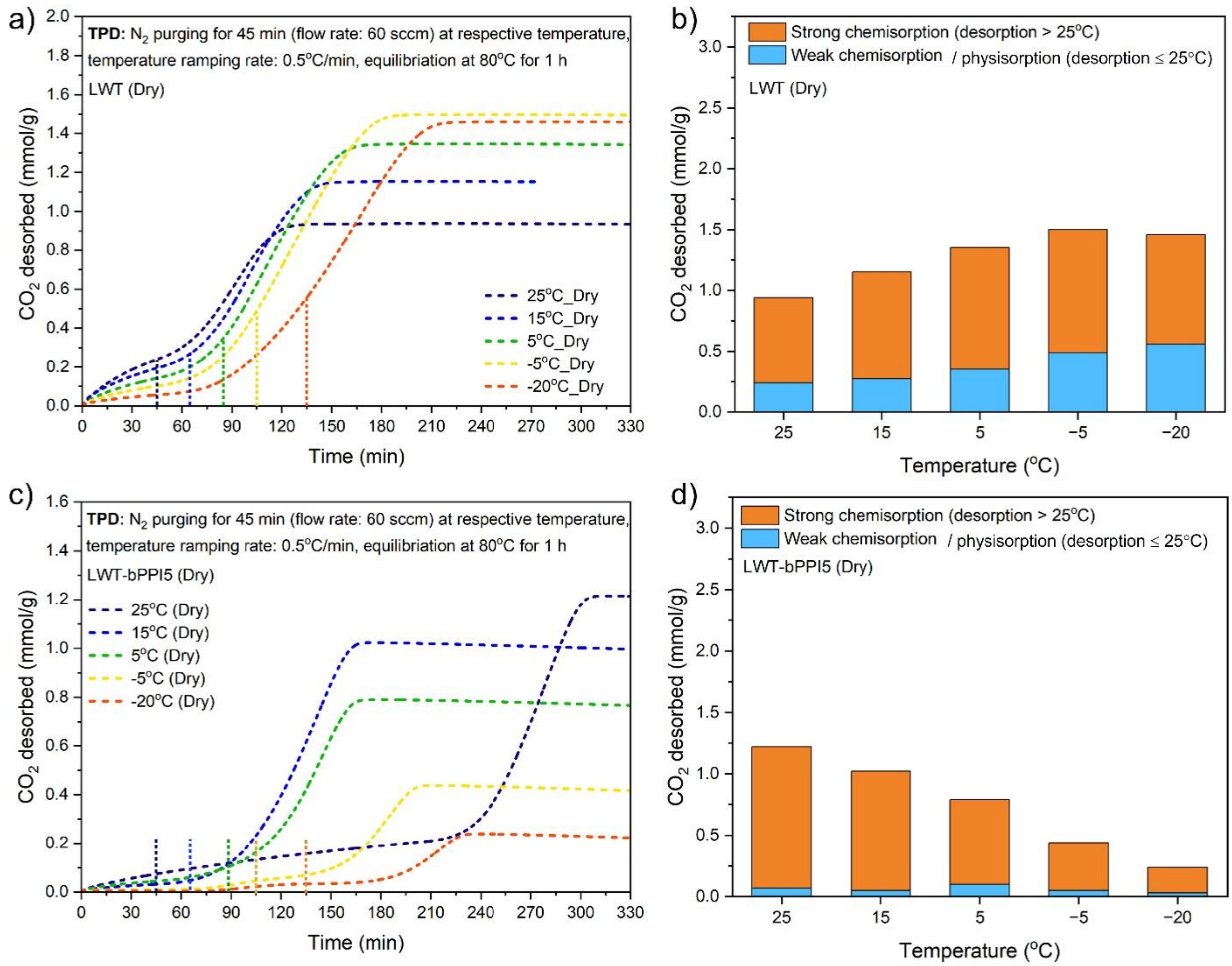

Fig. S21. Qualitative estimation of weak chemisorption/physisorption and strong chemisorption contribution under dry conditions. Quantified CO_2 desorption profiles from temperature programed desorption (TPD) experiments (ramp rate: 0.5°C min^{-1}) at different temperatures (25, 15, 5, -5, -20 °C) under dry (0% RH) conditions using (**a,b**) LWT and (**c,d**) LWT-bPPI5: (**a,c**) CO_2 desorbed (mmol g^{-1}) vs. Time (min) plot, (**b,d**) Contribution of weak chemisorption (including physisorption) and strong chemisorption in the total amount of desorbed dry/humid CO_2 from LWT and LWT-bPPI5 during the TPD.

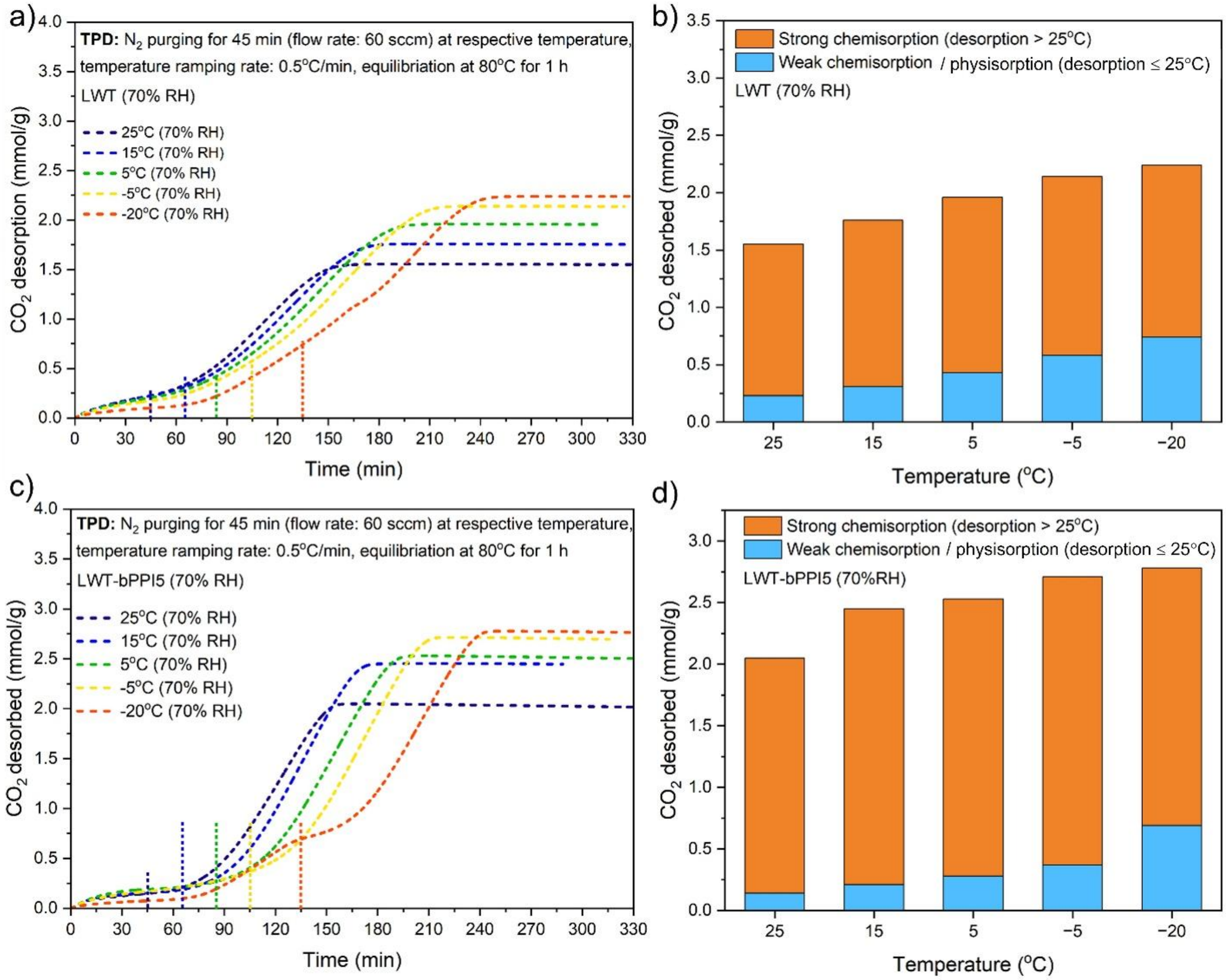

Fig. S22. Qualitative estimation of weak chemisorption/physisorption and strong chemisorption contribution under humid conditions. Quantified CO_2 desorption profiles from TPD experiments (ramp rate: 0.5°C min^{-1}) at different temperatures (25, 15, 5, -5, -20 °C) under humid (70% RH) conditions using (**a,b**) LWT and (**c,d**) LWT-bPPI5: (**a,c**) CO_2 desorbed (mmol g^{-1}) vs. Time (min) plot, (**b,d**) Contribution of weak chemisorption (including physisorption) and strong chemisorption in the total amount of desorbed dry/humid CO_2 from LWT and LWT-bPPI5 during the TPD.

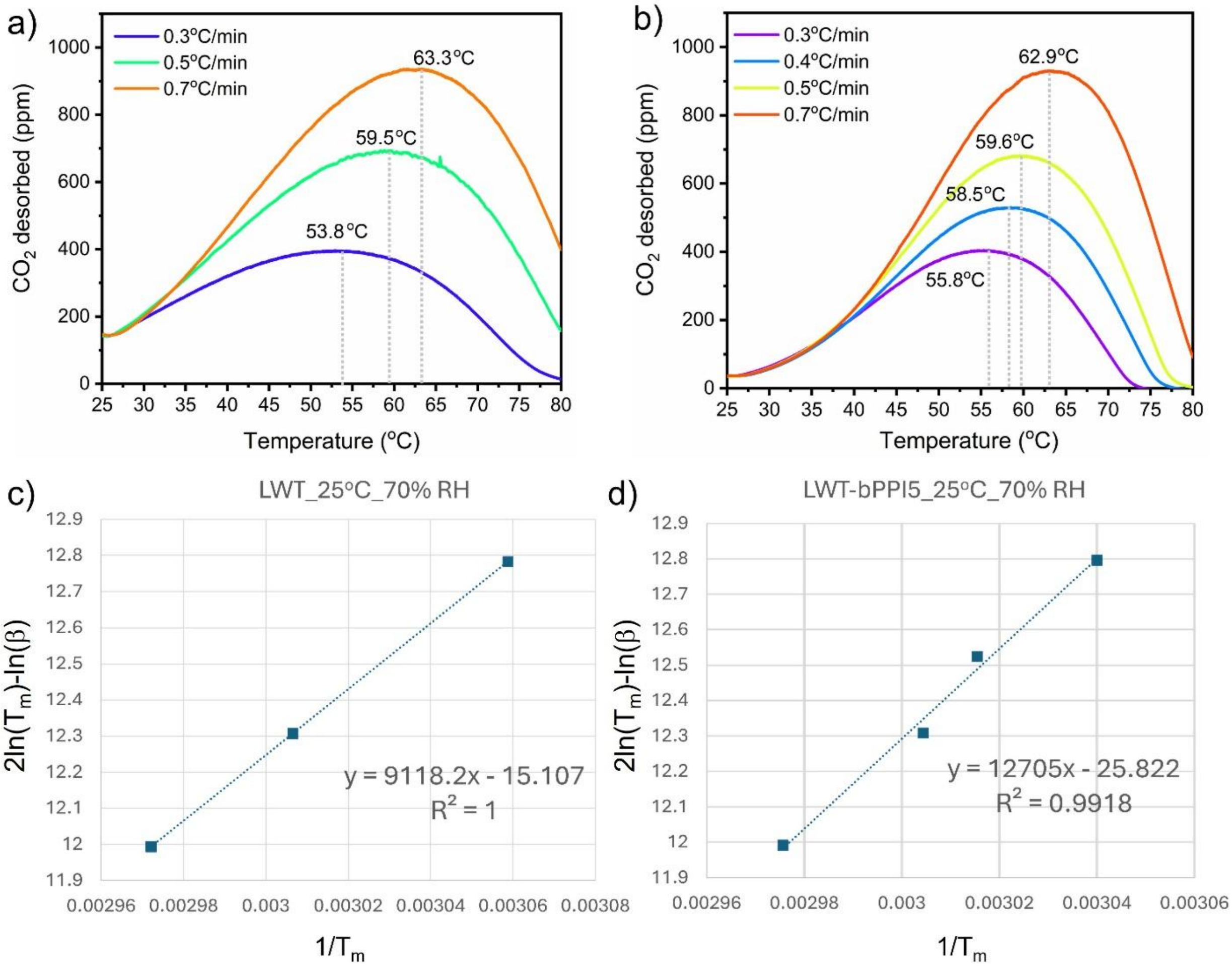

Fig. S23. Estimation of energy of desorption for the CO_2. Temperature-programmed desorption (TPD) study of CO_2 adsorbed on (**a**) LWT and (**b**) LWT-bPPI5 at 25 °C under 70% RH, carried out at heating rates of 0.3, 0.4, 0.5, and 0.7 C min^{-1}. Energy of CO_2 desorption determined from TPD analysis (*69*) for (**c**) LWT (calculated E_d: 75.8 kJ mol^{-1}) and (**d**) LWT-bPPI5 (calculated E_d: 105.6 kJ mol^{-1}) during 400 ppm CO_2/N_2 adsorption at 25ºC and 70% RH.

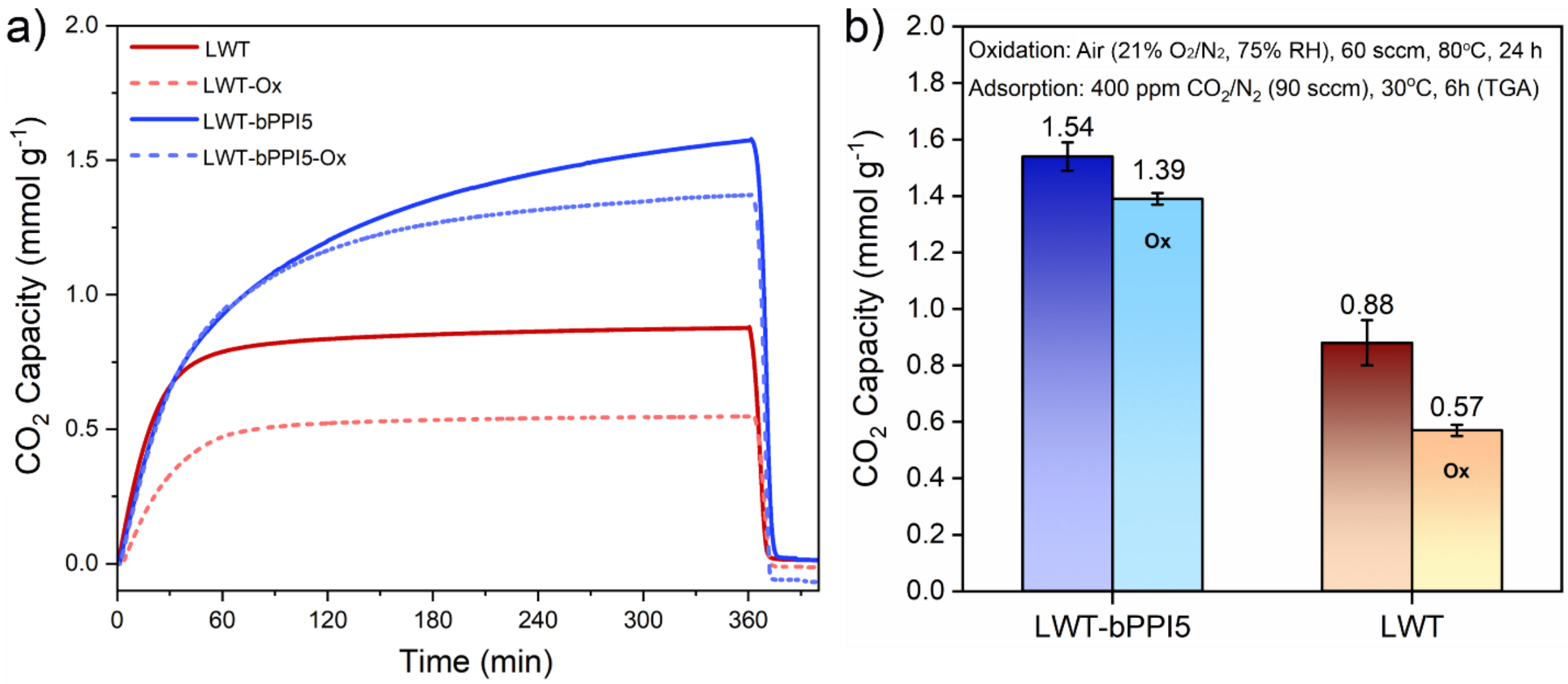

Fig. S24. DAC performance before and after oxidation of resins. (**a**) Thermogravimetric analysis of pristine LWT (solid red), oxidized LWT (dotted red), LWT-bPPI (solid blue) and oxidized LWT-bPPI5 (dotted blue) resins under 400 ppm CO_2/N_2 at 30°C (6 h adsorption, followed by activation at 80 °C under N_2 flow). (**b**) Bar chart showing the CO_2 uptake capacities of pristine and aged LWT and LWT-bPPI5. Aging was performed under humid air (21% O_2/N_2, 75% RH/ ≈ 1.95% absolute humidity) at 80°C for 24 h.

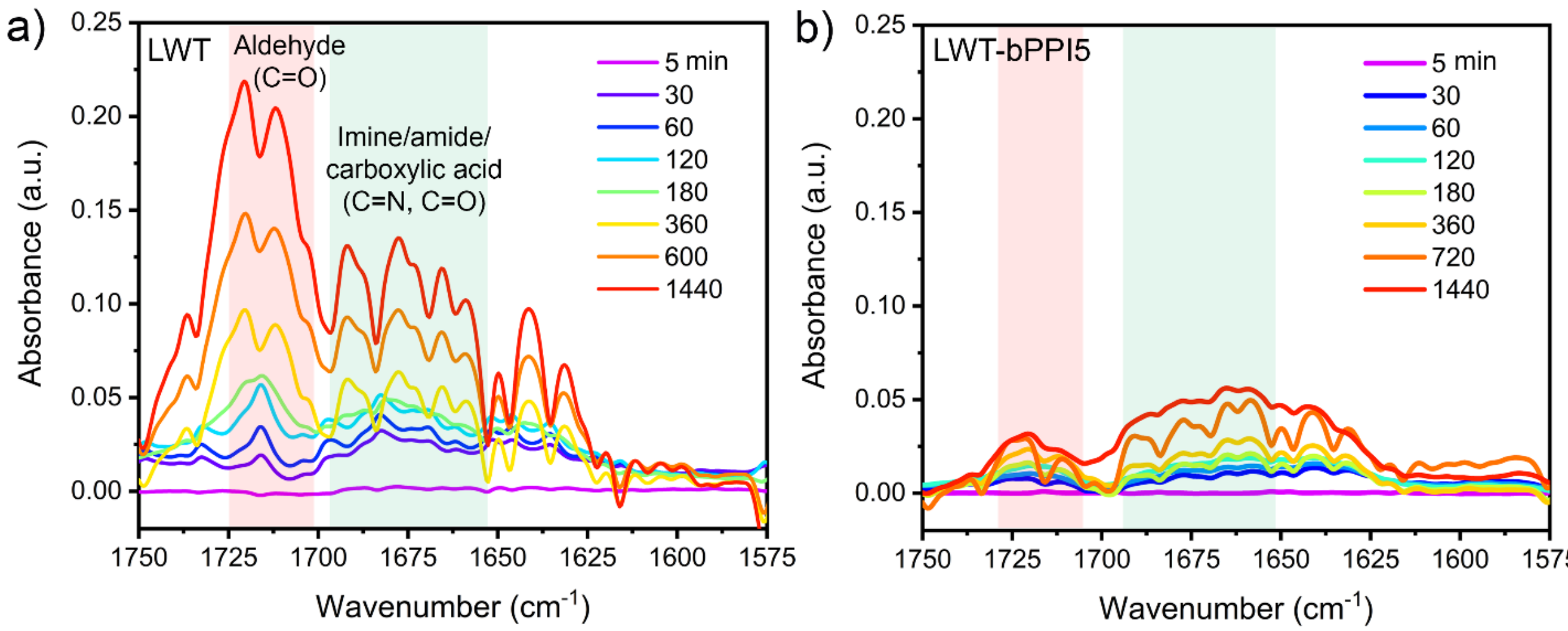

Fig. S25. *In situ* spectroscopic analysis of the resins for the accelerated oxidation under air. *In situ* DRIFT spectra (subtracted from the activated sample spectra) of (**a**) LWT and (**b**) LWT-bPPI5 as a function of time for accelerated oxidation under CO_2-free air (21% O_2/N_2, ≈ 1.95% absolute humidity, flow rate: 60 sccm) at 80°C. For better visualization of the vibrational features associated specifically with CO_2 sorption, each spectrum recorded during CO_2 dosing was baseline-corrected using the spectrum of the activated sample acquired immediately before the introduction of 400 ppm CO_2/N_2.

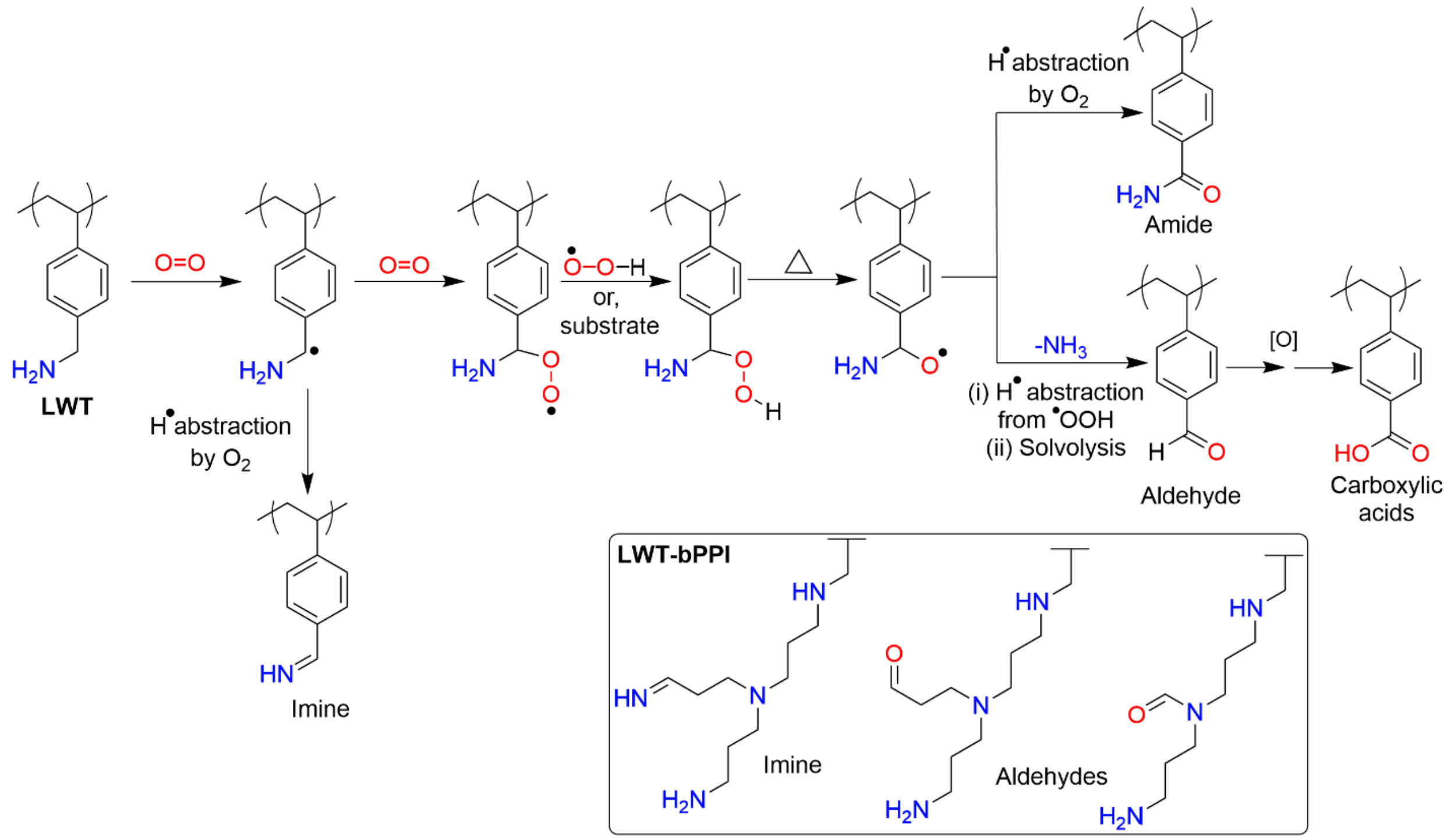

Fig. S26. A plausible mechanism along with the formation of various oxidation products from the LWT and LWT-bPPI resins. The proposed pathway is adapted from references (*40*), (*44*), and (*73*).

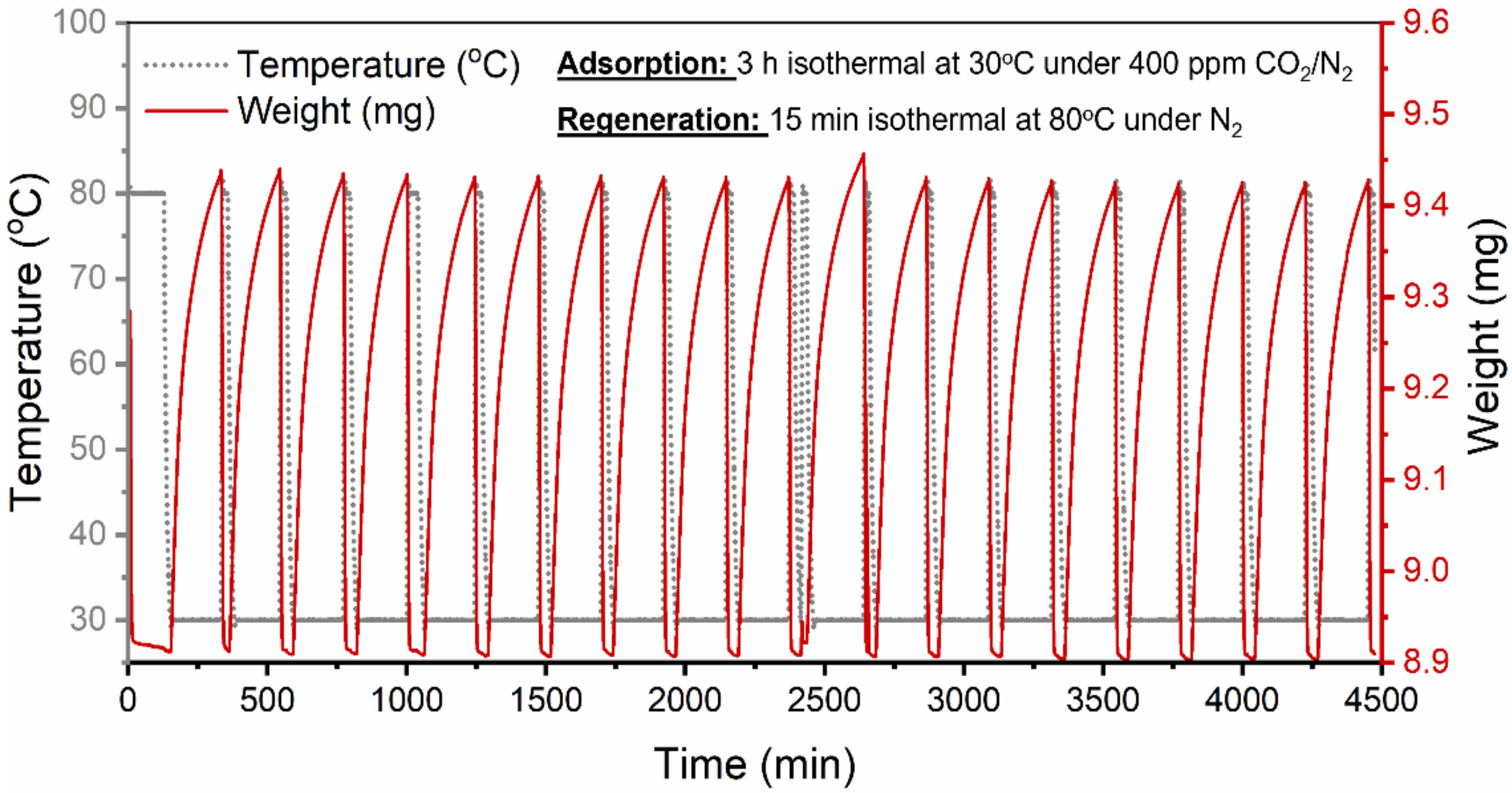

Fig. S27. Recyclability of LWT-bPPI5 under dry DAC ambient conditions using TGA. Cycling performance of LWT-bPPI5 under 400 ppm CO_2/N_2 at 30 °C using thermogravimetric analysis (3 h adsorption under 400 ppm CO_2/N_2 flow followed by 15 min regeneration at 80°C under N_2 flow). N.B.: During TGA recycling, the 3 h isothermal adsorption step at 30°C under 400 ppm CO_2 was inadvertently missed between the 10th and 11th regeneration cycles.

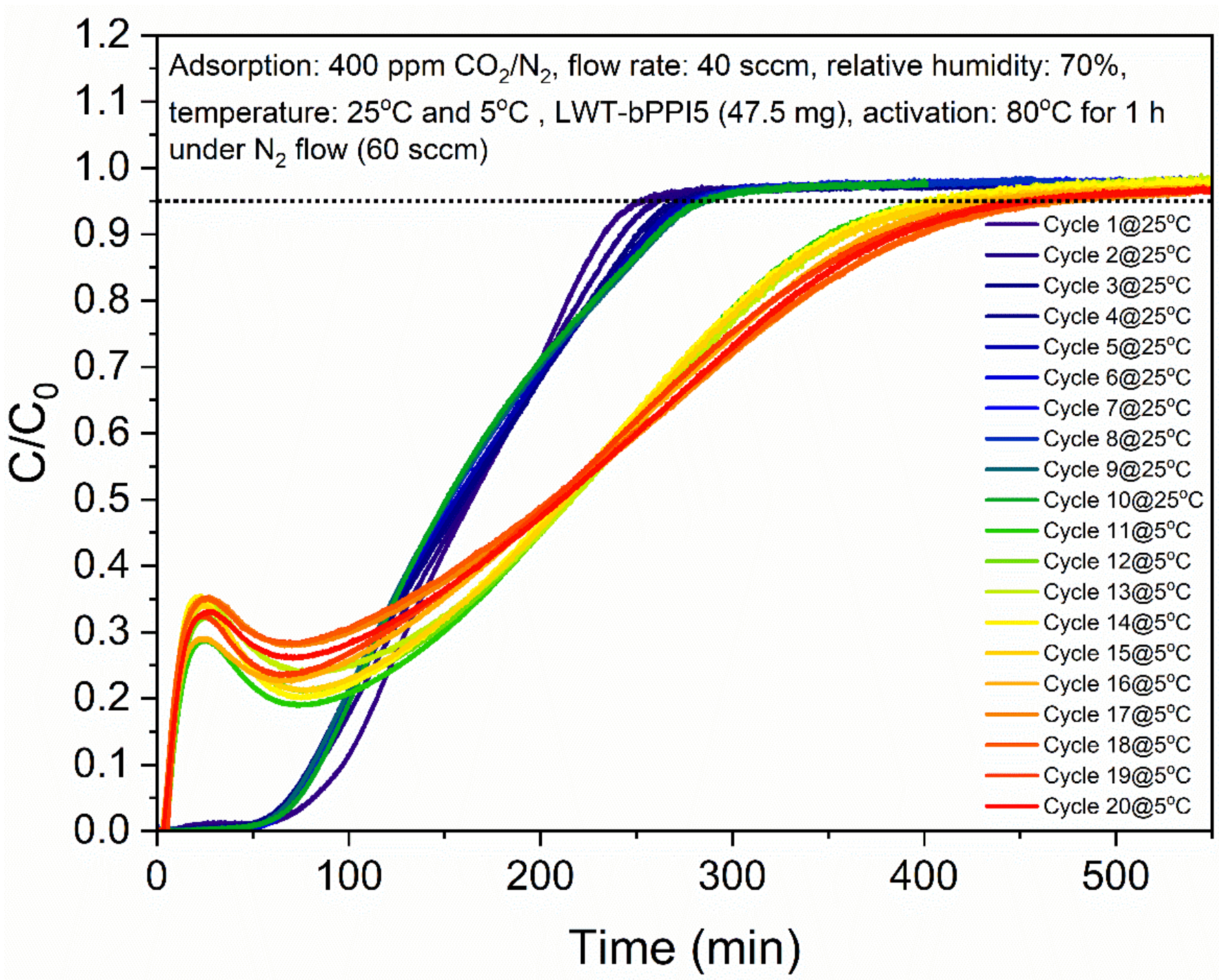

Fig. S28. Recyclability of LWT-bPPI5 under humid ambient and sub-ambient conditions using fixed bed dynamic breakthrough analysis. Breakthrough curves showing the recyclability of LWT-bPPI5 under DAC conditions at 25°C with 70% RH, and at 15°C with 70% RH. Adsorption was carried out using dry/humid ~400 ppm CO_2/N_2 (co-adsorption) at a flow rate of 40 sccm, and each run was terminated when the outlet CO_2 concentration reached 95% of the inlet concentration (C_0). Regeneration was performed after each adsorption cycle at 80°C under N_2 flow (60 sccm) for 1 h. The featured breakthrough curve at 5 °C and 70% RH likely originates from competitive water adsorption during the initial stage; once enough water is taken up, the amines become sufficiently hydrated, resulting in enhanced amine mobility and the onset of CO_2 adsorption.

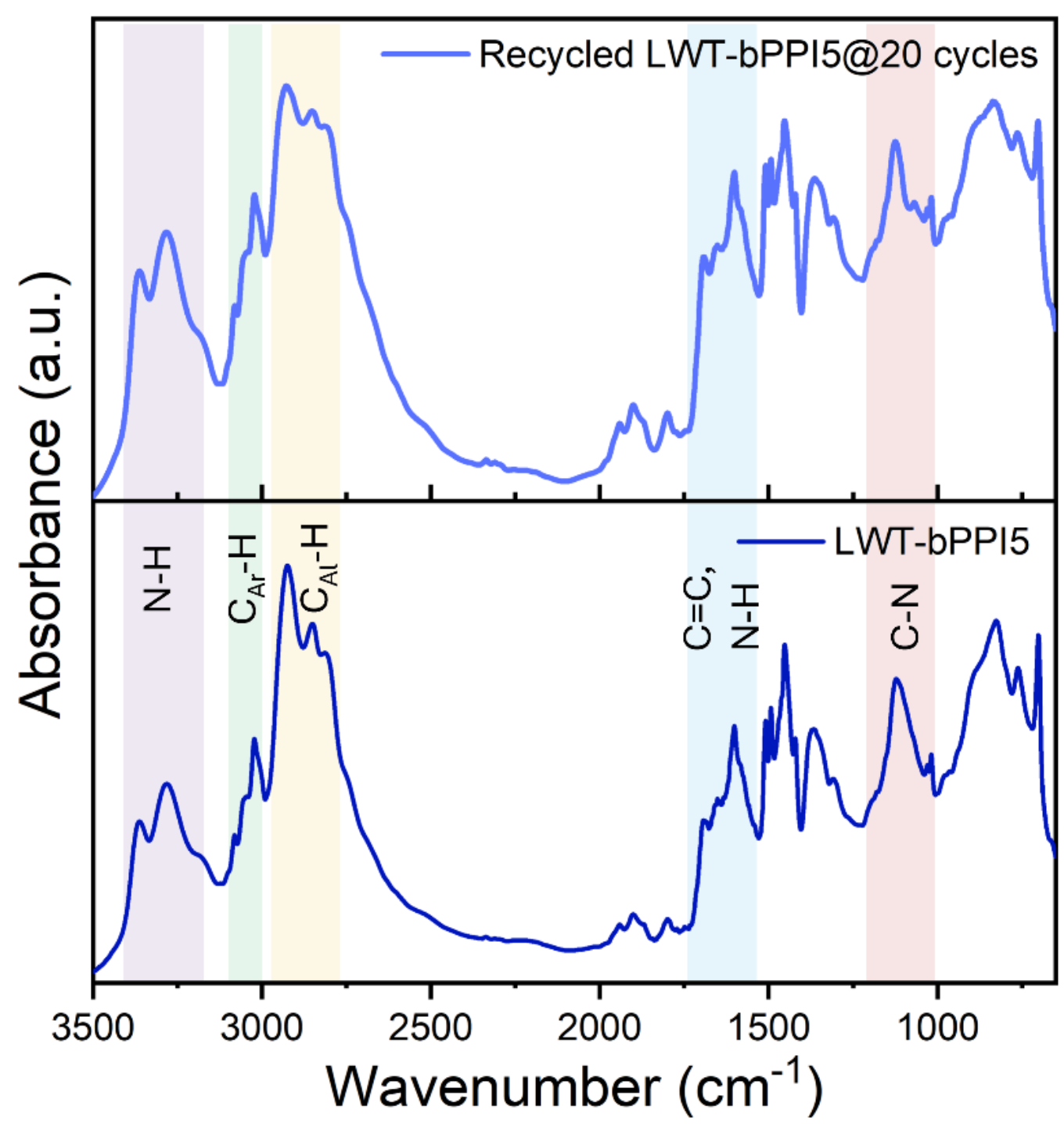

Fig. S29. Structural characterization of recycled LWT-bPPI5. DRIFTS analysis of pristine and recycled LWT-bPPI5 after 20 adsorption-desorption cycles under ambient (25°C) and sub-ambient (5°C) humid conditions (70% RH). The nearly identical spectral features before and after cycling highlights the excellent structural robustness of the LWT-bPPI5 resin.

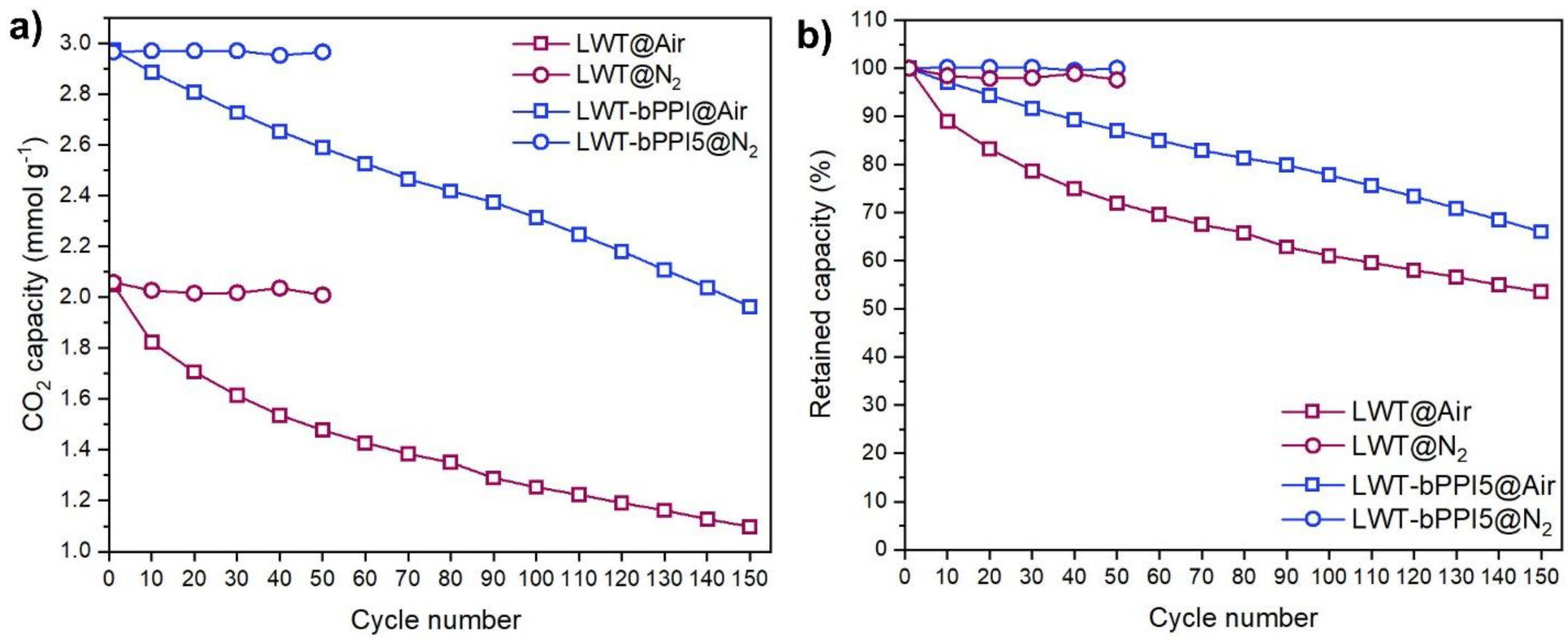

Fig. S30. Recyclability of LWT and LWT-bPPI5 under simulated flue gas conditions and regeneration under air/nitrogen. Thermogravimetric analysis of CO_2 uptake showing **(a)** absolute capacity (mmol g^{-1}) and **(b)** retained capacity (%, in points and lines) of LWT (red square) and LWT-bPPI5 (blue square) under simulated flue gas conditions (15% CO_2/N_2 at 40°C, 0% RH) and regeneration under CO_2-free air at 80°C at different numbers of adsorption-desorption cycles. The capacity of LWT (red circle) and LWT-bPPI5 (blue circle) under simulated flue gas conditions (15% CO_2/N_2 at 40°C, 0% RH) and regeneration under N_2 at 80°C at different numbers of adsorption-desorption cycles.

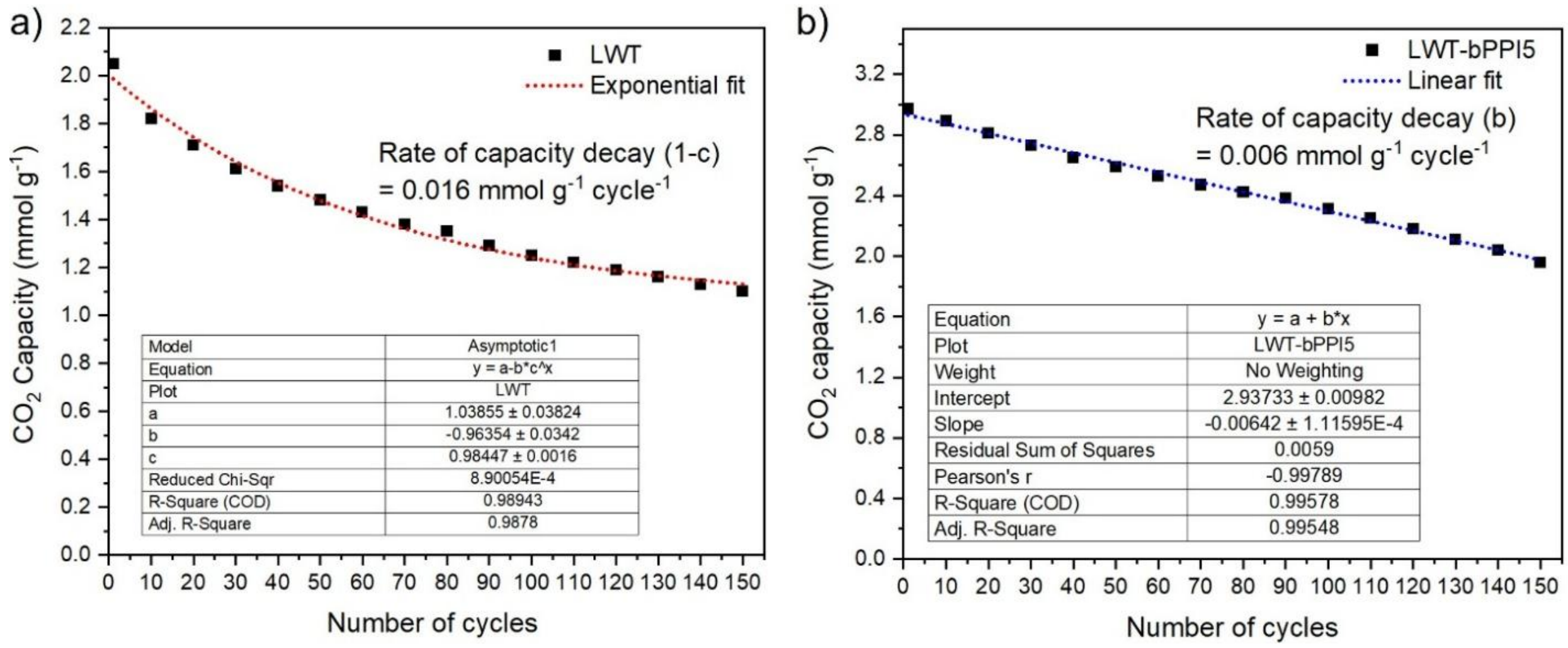

Fig. S31. Recyclability of LWT and LWT-bPPI5 under simulated flue gas conditions and regeneration under air. Thermogravimetric analysis of CO_2 uptake (mmol g^{-1}, in points) of **(a)** LWT and **(b)** LWT-bPPI5 under simulated flue gas conditions (15% CO_2/N_2 at 40°C, 0% RH) at different numbers of adsorption-desorption cycles. The dotted line indicates the CO_2 capacity decay of LWT (exponential fit) and for LWT-bPPI5 (linear fit) as a function of cycle number.

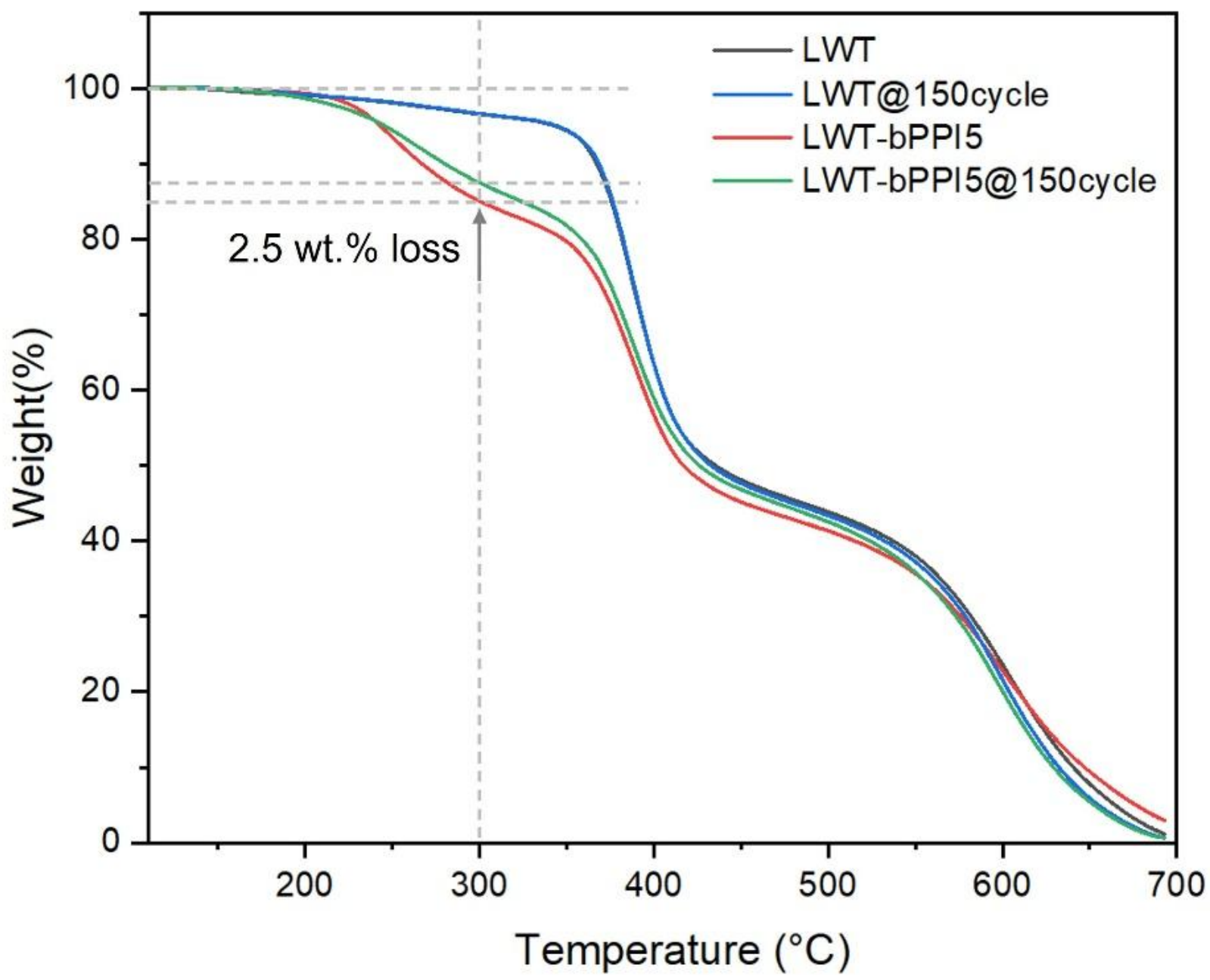

Fig. S32. Pyrolysis thermogravimetric analysis of pristine and 150 times cycled LWT and LWT-bPPI5. Observed ~2.5 wt.% loss in amine loading for LWT-bPPI5, is likely due to oxidation-induced cleavage of the grafted bPPI chains. In contrast, pristine LWT showed no measurable weight loss, which is consistent with the proposed oxidation pathway where benzylic amines are converted to carbonyl and/or imine functionalities without significant loss of mass.

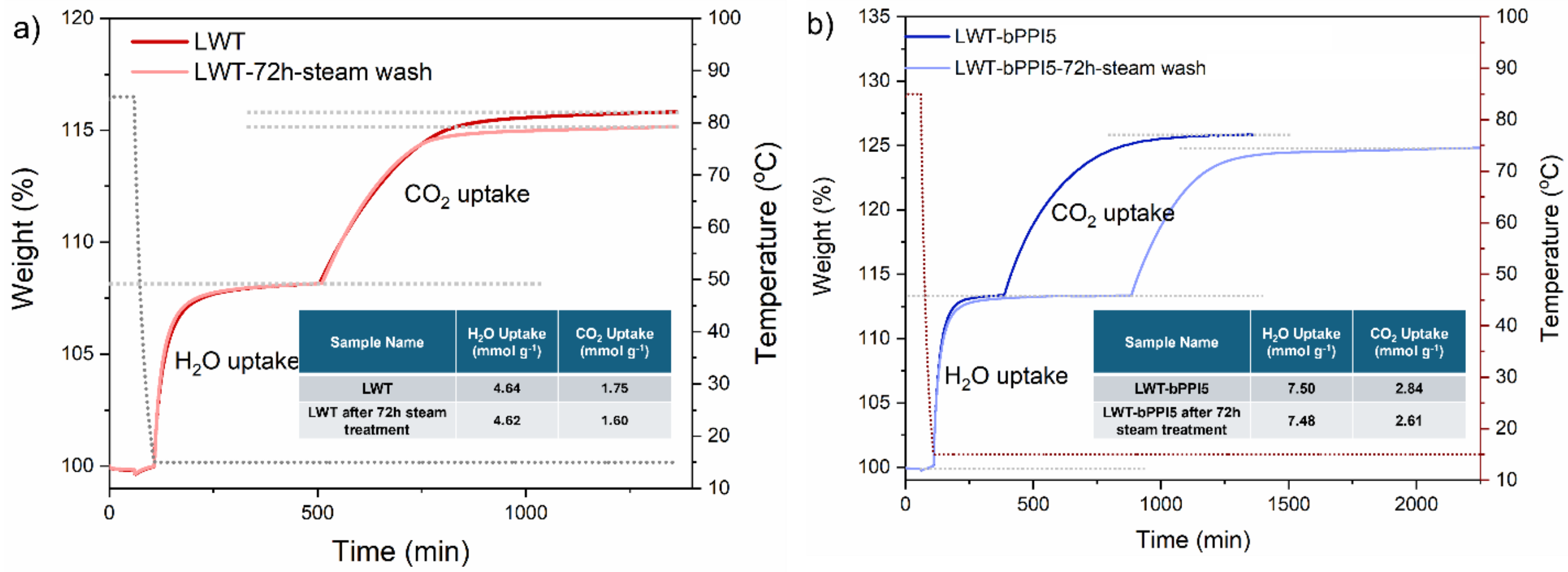

Fig. S33. Stability of LWT-bPPI5 resin under steam. Thermogravimetric analysis of CO_2 sorption on (**a**) pristine LWT (red), and 72h steam-washed LWT (light red); (**b**) pristine LWT-bPPI5 (blue) and 72 h steam-washed LWT-bPPI5 (light blue), under 400 ppm CO_2/N_2 at 15°C and 54% relative humidity (pre-saturation). The sample was placed in a cup made of filter paper and kept in a pre-scribed height from the boiling deionized water for 72 h. The recovered sample was dried overnight at ambient temperature followed by activation at 85°C under N_2 and CO_2 sorption using a TGA TA Q5000.

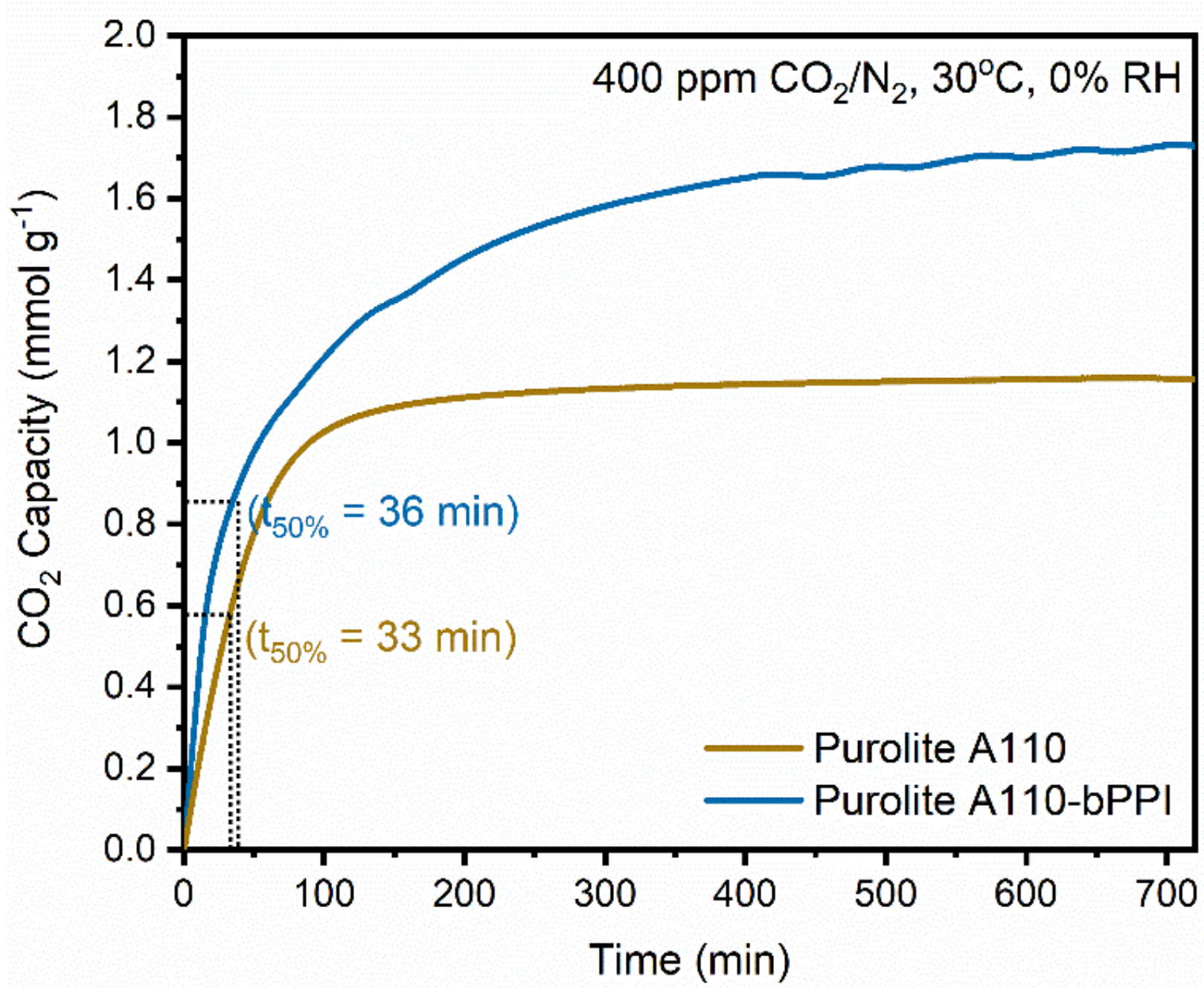

Fig. S34. Comparative DAC performance of pristine and bPPI-grafted Purolite A110 resin. Thermogravimetric analysis of CO_2 adsorption on Purolite A110 and Purolite A110-bPPI, under 400 ppm CO_2/N_2 at 30°C and 0% RH. Prior to each measurement, the sample was activated at 80°C under a N_2 atmosphere for 2 h. $t_{50\%}$ indicates the time (in min) required to reach 50% of the overall capacity.

Table S5. Comparative table based on the CO_2 capture performance of benchmark poly(amine)-functionalized solid sorbents under dry and humid conditions.

Entry	Amine sorbent	Capacity under DAC condition (mmol g^{-1})			Capacity under flue gas (10-15% CO_2) conditions	Cycling stability	Humidity stability / Steam stability	Reference
		Ambient conditions (25°C-30°C)		Sub-ambient (< 20°C) humid conditions (% RH)				
		Dry	Humid (% RH)					
1	LWT-bPPI5	1.41 ± 0.16	2.58 (90%), 2.28 (70%)	3.1 (-5°C, 70%), 2.95 (-20°C, 70%)	3.34 at 40°C (15% CO_2)	19 dry cycles, 20 humid cycles (no capacity loss, DAC conditions), 150 cycles (flue gas conditions, decay rate: 0.006 mmol g^{-1} $cycle^{-1}$ for LWT-bPPI5, 0.016 mmol g^{-1} $cycle^{-1}$ for LWT)	No drop in capacity up to 90% RH, only ~8 wt.% loss in capacity after 72 h steam treatment	*Present work*
	LWT	0.96	1.78 (70%)	2.42 (-5°C, 70%), 2.40 (-20°C, 70%),	2.15 at 40°C (15% CO_2)		Stable under humid conditions (70%), ~9 wt.% loss in capacity after 72 h steam treatment	
**Covalent Organic Frameworks (COFs)**								
2	COF-1000	1.31	2.19 (75%)	-	-	50 cycles (DAC conditions)	No drop in capacity up to 75%	74
3	COF-998	0.38	1.2 (75%)	-	-	-	No drop in capacity up to 75%	74
4	ImCOF-TAEA	0.46	1.09 (70%)	1.25 (15°C, 70%), 1.05 (-5°C, 70%)	1.35 ± 0.15 at 30°C (10% CO_2)	13 cycles (DAC conditions)	No drop in capacity up to 70%	75
5	COF-999	0.96	2.09 (75%)	-	3.24 at 25°C (15% CO_2, 75% RH)	100 cycles (DAC conditions)	No drop in capacity up to 75%	27
6	COF-709	0.48	1.24 (75%)	-	1.77 at 25°C (4% CO_2)	10 cycles (DAC conditions)	No drop in capacity up to 75%	76

7	COF-609	0.30	0.39 (50%)	-	1.50 at 25°C (15% CO_2)	-	No drop in capacity up to 75%	77
				**Metal Organic Frameworks (MOFs)**				
8	TEPA(50)-Mg2(dobpdc), PEI(50)-Mg2(dobpdc)	0.54, 0.59	3.9 (70%), 1.86 (70%)	-	-	11-68% drop in capacity within 3rd cycle	Unstable under humid conditions (70%)	60
9	NUS-110	0.89	1.31 (30%), 0.34 (80%)	-	-	20 cycles (DAC conditions)	~62% drop in capacity at 80%	78
10	Cr-BDC-TAEA-BDE (branched-linear)	-	-	-	2.2 at 0.15 bar CO_2 and 40°C	100 cycles (flue gas conditions)	Stable under humid conditions (80%)	79
11	MOF-808-Lys	0.61	1.21 (50%)	-	-	10 cycles (DAC conditions)	Stable under humid conditions (50%)	80
12	CA/MIL-101/PEI	0.57	-	1.43 (-20°C) (78%)	-	14 cycles (DAC conditions)	Stable under humid sub-ambient conditions (78%)	81
13	MIL-101(Cr)-TEPA (30wt%)	0.11	0.38 (70%)	1.9 (-20°C) (70%)	-	-	Stable under humid conditions (70%)	59
14	2-UiO-EDA	0.04	0.44 (30%)	-	-	10 cycles (15% loss, DAC conditions)	Stable up to 30%	82
15	Ni-MOF/SIL	-	0.58 (2.5%)	-	-	-	Stable up to 10%	83
16	SIFSIX-3-Cu	1.24	-	-	-	-	Stable up to 74%	84
				**Resins**				
17	PS-CH2PEI600	-	-	-	1.24 (15% CO_2/N_2, 80% RH at 25°C)	10 cycles (Flue gas conditions)	Stable under humid conditions (80%)	85
18	PEI-HP20	1.25	1.35 (50%)	-	-	-	Stable under humid conditions (50%)	86
19	PPVAm-3.3	0.9	0.95 (50%)	1.3 (5°C) (0%)	-	-	Stable under humid conditions (50%)	32

20	Purolite A110	1.30	1.76 (33%)	-	-	-	-	38
21	HIPE templated p(NMe_3^+-MS OH^-)	-	0.49 (20%)	-	-	-	Stable under humid conditions (20%)	87
				**Silica**				
22	MAHSM	1.68	1.59 (85%)	-	-	-	Stable under humid conditions (85%)	88
23	DT- PE-MCM-41	0.61	0.54 (73%)	-	-	-	Stable under humid conditions (73%)	89
24	AEAPTMS-SBA-15	0.98	1.72 (100%)	-	-	-	Stable under humid conditions	25
25	ED-Silica gel	0.40	0.44 (40%)	-	-	-	Stable under humid conditions (40%)	90
				**Alumina**				
26	TRI-Alumina	0.48	0.74 (50%)	-	-	-	-	91
27	γ-Alumina@ 40wt%PEI	1.26	2 (70%)	1.8 (-20°C) (70%)	-	5 cycles (sub-ambient DAC conditions)	Stable under humid conditions (70%)	92
28	TRI-PE-MCM-41	0.98	1.53 (64%)	-	-	-	-	24

REFERENCES

1. S. Zhao, Y. Zhang, L. Li, J. Feng, W. Qiu, Y. Wang, Z. Huang, H. Lin, Amine-functionalized macroporous resin for direct air capture with high CO_2 capacity in real atmospheric conditions: Effects of moisture and oxygen. *Sep. Purif. Technol.* **350**, 127999 (2024).

2. Emissions gap report 2017, A UN environment synthesis report, https://www.unep.org/resources/emissions-gap-report-2017 [accessed December 2025].

3. Y. Gao, X. Gao, X. Zhang, The 2°C global temperature target and the evolution of the long-term goal of addressing climate change-from the united nations framework convention on climate change to the paris agreement. *Engineering* **3**, 272–278 (2017).

4. N. S. Council, *Carbon dioxide removal: NOAA State of the Science factsheet* 2024, https://www.climate.gov/news-features/understanding-climate/carbon-dioxide-removal-noaa-state-science-factsheet#:∼:text=The%20State%20of%20Carbon%20Dioxide%20Removal%20report,already%20occurring%2C%20primarily%20through%20conventional%20CDR%20methods [accessed December 2025].

5. E. S. Sanz-Pérez, C. R. Murdock, S. A. Didas, C. W. Jones, Direct capture of CO_2 from ambient air. *Chem. Rev.* **116**, 11840–11876 (2016).

6. A. Majumdar, J. Deutch, Research opportunities for CO_2 utilization and negative emissions at the Gigatonne scale. *Joule* **2**, 805–809 (2018).

7. K. S. Lackner, S. Brennan, J. M. Matter, A.-H. A. Park, A. Wright, B. van der Zwaan, The urgency of the development of CO_2 capture from ambient air. *Proc. Natl. Acad. Sci. U.S.A.* **109**, 13156–13162 (2012).

8. J. Fuhrman, H. McJeon, P. Patel, S. C. Doney, W. M. Shobe, A. F. Clarens, Food-energy-water implications of negative emissions technologies in a +1.5 °C future. *Nat. Clim. Change* **10**, 920–927 (2020).

9. D. Fu, M. E. Davis, Carbon dioxide capture with zeotype materials. *Chem. Soc. Rev.* **51**, 9340–9370 (2022).

10. X. Shi, H. Xiao, H. Azarabadi, J. Song, X. Wu, X. Chen, K. S. Lackner, Sorbents for the direct capture of CO_2 from ambient air. *Angew. Chem. Int. Ed.* **59**, 6984–7006 (2020).

11. L. Qiu, N. Mokhtarinori, H. Liu, D.-e. Jiang, Z. Yang, S. Dai, The carbon challenge: Design, synthesis, and chemisorption behavior of solid sorbents in direct air capture of carbon dioxide. *Mater. Today Energy* **47**, 101740 (2025).

12. R. A. Yang, S. Cho, S. N. Hughes, M. L. Sarazen, Implications of defect density and polymer interactions for CO_2 capture on amine-functionalized MIL-101(Cr). *ChemSusChem* **17**, e202400249 (2024).

13. M. Ozkan, A.-A. Akhavi, W. C. Coley, R. Shang, Y. Ma, Progress in carbon dioxide capture materials for deep decarbonization. *Chem* **8**, 141–173 (2022).

14. X. Zhu, W. Xie, J. Wu, Y. Miao, C. Xiang, C. Chen, B. Ge, Z. Gan, F. Yang, M. Zhang, D. O'Hare, J. Li, T. Ge, R. Wang, Recent advances in direct air capture by adsorption. *Chem. Soc. Rev.* **51**, 6574–6651 (2022).

15. T. M. McDonald, W. R. Lee, J. A. Mason, B. M. Wiers, C. S. Hong, J. R. Long, Capture of carbon dioxide from air and flue gas in the alkylamine-appended metal-organic framework mmen-Mg_2(dobpdc). *J. Am. Chem. Soc.* **134**, 7056–7065 (2012).

16. A. K. Sekizkardes, P. Wang, J. Hoffman, S. Budhathoki, D. Hopkinson, Amine-functionalized porous organic polymers for carbon dioxide capture. *Mater. Adv.* **3**, 6668–6686 (2022).

17. A. J. Huang, A. K. Gupta, H. Z. H. Jiang, H. Zhuang, M. B. Wenny, R. A. Klein, H. Kwon, K. R. Meihaus, H. Furukawa, C. M. Brown, J. A. Reimer, W. A. de Jong, J. R. Long, Phase change-mediated capture of carbon dioxide from air with a molecular triamine network solid. *J. Am. Chem. Soc.* **147**, 10519–10529 (2025).

18. M. Ozkan, S. P. Nayak, A. D. Ruiz, W. Jiang, Current status and pillars of direct air capture technologies. *iScience* **25**, 103990 (2022).

19. A. Grimm, G. J. Kramer, M. Gazzani, How would ideal sorbents improve the technical and economic performance of adsorption-based direct air capture? *Energy Fuel* **38**, 18781–18799 (2024).

20. H. E. Holmes, R. P. Lively, M. J. Realff, Defining targets for adsorbent material performance to enable viable BECCS processes. *JACS Au* **1**, 795–806 (2021).

21. H. E. Holmes, S. Banerjee, A. Vallace, R. P. Lively, C. W. Jones, M. J. Realff, Tuning sorbent properties to reduce the cost of direct air capture. *Energy Environ. Sci.* **17**, 4544–4559 (2024).

22. M. Chintapalli, S. Meckler, G. Iftime, R. Pandey, M. Louie, E. Shin, M. Beh, Tunable, rapid uptake, aminopolymer aerogel sorbent for direct air capture of CO_2. 2024. US patent number: US11944933B2.

23. Report of the Basic Energy Sciences Roundtable on Foundational Science for Carbon Dioxide Removal Technologies. U.S. Department of Energy's Office of Basic Energy Science, 2022.

24. Y. Belmabkhout, R. Serna-Guerrero, A. Sayari, Adsorption of CO_2-containing gas mixtures over amine-bearing pore-expanded MCM-41 silica: Application for gas purification. *Ind. Eng. Chem. Res.* **49**, 359–365 (2010).

25. S. Choi, J. H. Drese, P. M. Eisenberger, C. W. Jones, Application of amine-tethered solid sorbents for direct CO_2 capture from the ambient air. *Environ. Sci. Technol.* **45**, 2420–2427 (2011).

26. J. Wang, H. Huang, M. Wang, L. Yao, W. Qiao, D. Long, L. Ling, Direct capture of low-concentration CO_2 on mesoporous carbon-supported solid amine adsorbents at ambient temperature. *Ind. Eng. Chem. Res.* **54**, 5319–5327 (2015).

27. Z. Zhou, T. Ma, H. Zhang, S. Chheda, H. Li, K. Wang, S. Ehrling, R. Giovine, C. Li, A. H. Alawadhi, M. M. Abduljawad, M. O. Alawad, L. Gagliardi, J. Sauer, O. M. Yaghi, Carbon dioxide capture from open air using covalent organic frameworks. *Nature* **635**, 96–101 (2024).

28. Z. Zhu, H. Tsai, S. T. Parker, J.-H. Lee, Y. Yabuuchi, H. Z. H. Jiang, Y. Wang, S. Xiong, A. C. Forse, B. Dinakar, A. Huang, C. Dun, P. J. Milner, A. Smith, P. G. Martins, K. R. Meihaus, J.

J. Urban, J. A. Reimer, J. B. Neaton, J. R. Long, High-capacity, cooperative CO_2 capture in a diamine-appended metal-organic framework through a combined chemisorptive and physisorptive mechanism. *J. Am. Chem. Soc.* **146**, 6072–6083 (2024).

29. W. R. Lee, S.-Y. Hwang, D. W. Ryu, K. S. Lim, S. S. Han, D. Moon, J. Choi, C. S. Hong, Diamine-functionalized metal-organic framework: Exceptionally high CO_2 capacities from ambient air and flue gas, ultrafast CO_2 uptake rate, and adsorption mechanism. *Energy Environ. Sci.* **7**, 744–751 (2014).

30. Y. Wang, F. Gao, Y. Niu, J. Zhang, K. Chen, Y. Zhou, X. Tang, S. Zhao, H. Yi, Recent advancements in carbon capture materials research: Innovative optimization of materials synthesis and engineering applications. *J. Mater. Chem. A* **13**, 23323–23353 (2025).

31. H. Li, A. Dilipkumar, S. Abubakar, D. Zhao, Covalent organic frameworks for CO_2 capture: From laboratory curiosity to industry implementation. *Chem. Soc. Rev.* **52**, 6294–6329 (2023).

32. J. Baker, P. Muldoon, J. A. Steckel, A. K. Sekizkardes, Porous polyvinylamine adsorbents with low temperature regenerability for direct air capture. *ACS Appl. Polym. Mater.* **6**, 14278–14282 (2024).

33. D. Albani, C. Gropp, T. Aztiria, C. Limone, A. Vargas, Uses of amino sorbents for capturing of CO_2 from gas streams. 2024, International Patent No.: WO 2024/132899 A1.

34. A. P. Tirio, Process and apparatus for carbon dioixde capture via ion exchange resins. United States 2011, US Patent No.: US 2011/0088550 A1.

35. Y. Gao, Solid primary amine and amidine-based materials for adsorptive gas separation of CO_2, with improved air, water and temperature tolerance. 2024, International Patent No.: WO 2024/110902 A1.

36. L. B. Hamdy, R. J. Wakeham, M. Taddei, A. R. Barron, E. Andreoli, Epoxy cross-linked polyamine CO_2 sorbents enhanced via hydrophobic functionalization. *Chem. Mater.* **31**, 4673–4684 (2019).

37. P. Narayanan, S.-Y. Kim, D. Alhazmi, C. W. Jones, R. P. Lively, Self-supported branched poly(ethylenimine) monoliths from inverse template 3D printing for direct air capture. *ACS Appl. Mater. Interfaces* **17**, 10696–10709 (2025).

38. M.-Y. A. Low, D. Danaci, H. Azzan, R. T. Woodward, C. Petit, Measurement of physicochemical properties and CO_2, N_2, Ar, O_2, and H_2O unary adsorption isotherms of Purolite A110 and Lewatit VP OC 1065 for application in direct air capture. *J. Chem. Eng. Data* **68**, 3499–3511 (2023).

39. W. R. Alesi Jr., J. R. Kitchin, Evaluation of a primary amine-functionalized ion-exchange resin for CO_2 capture. *Ind. Eng. Chem. Res.* **51**, 6907–6915 (2012).

40. W. Buijs, Direct air capture of CO_2 with an amine resin: A molecular modeling study of the oxidative deactivation mechanism with O_2. *Ind. Eng. Chem. Res.* **58**, 17760–17767 (2019).

41. G. N. Tomaraei, S. Binney, R. Stratton, H. Zhuang, J. L. Wade, H_2O and CO_2 sorption in ion-exchange sorbents: Distinct interactions in amine versus quaternary ammonium materials. *ACS Appl. Mater. Interfaces* **17**, 53547–53562 (2025).

42. S. B. Petersen, E. Forbes, J. A. Sawada, T. H. Pedersen, Lewatit 1065 VP OC for direct air capture; an analysis of adsorption characteristics using dynamic column breakthrough. *Sep. Purif. Technol.* **380**, 135224 (2026).

43. R. Hunt, J. Gillbanks, J. Czapla, Z. Wan, C. Karmelich, C. White, C. Wood, Representative longevity testing of direct air capture materials. *Chem. Eng. J.* **481**, 148901 (2024).

44. S. H. A. M. Leenders, G. Pankratova, J. Wijenberg, J. Romanuka, F. Gharavi, J. Tsou, M. Infantino, L. van Haandel, S. van Paasen, P.-E. Just, Amine adsorbents stability for post-combustion CO_2 capture: Determination and validation of laboratory degradation rates in a multi-staged fluidized bed pilot plant. *ChemSusChem* **16**, e202300930 (2023).

45. S. H. Pang, L.-C. Lee, M. A. Sakwa-Novak, R. P. Liyely, C. W. Jones, design of aminopolymer structure to enhance performance and stability of CO_2 sorbents: Poly(propylenimine) vs poly(ethylenimine). *J. Am. Chem. Soc.* **139**, 3627–3630 (2017).

46. S. H. Pang, R. P. Lively, C. W. Jones, Oxidatively-stable linear poly(propylenimine)-containing adsorbents for CO_2 capture from ultradilute streams. *ChemSusChem* **11**, 2628–2637 (2018).

47. J. Hoffman, L. Proaño, C. W. Jones, Role of polymer architecture in CO_2 capture from air using supported poly(alkylenimine)s: Linear vs branched polymers. *ACS Appl. Polym. Mater.* **7**, 15671–15681 (2025).

48. J. Deng, Z. Liu, Z. Du, W. Zou, C. Zhang, Fabrication of PEI-grafted porous polymer foam for CO_2 capture. *J. Appl. Polym. Sci.* **136**, 47844 (2019).

49. E. H. Schacht, E. J. Goethals, Cationic polymerization of cyclic amines, 3. Polymerization of azetidine. *Macromol. Chem.* **175**, 3447–3459 (1974).

50. H. Oike, M. Washizuka, Y. Tezuka, Cationic ring-opening polymerization of *N*-phenylazetidine. *Macromol. Chem. Phys.* **201**, 1673–1678 (2000).

51. M. L. Sarazen, C. W. Jones, Insights into azetidine polymerization for the preparation of poly(propylenimine)-based CO_2 adsorbents. *Macromolecules* **50**, 9135–9143 (2017).

52. T. Gleede, L. Reisman, E. Rieger, P. C. Mbarushimana, P. A. Rupar, F. R. Wurm, Aziridines and azetidines: Building blocks for polyamines by anionic and cationic ring-opening polymerization. *Polym. Chem.* **10**, 3257–3283 (2019).

53. M. L. Sarazen, M. A. Sakwa-Novak, E. W. Ping, C. W. Jones, Effect of different acid initiators on branched poly(propylenimine) synthesis and CO_2 sorption performance. *ACS Sustain. Chem. Eng.* **7**, 7338–7345 (2019).

54. W. Chaikittisilp, S. A. Didas, H. J. Kim, C. W. Jones, Vapor-phase transport as a novel route to hyperbranched polyamine-oxide hybrid materials. *Chem. Mater.* **25**, 613–622 (2013).

55. H.-J. Kim, W. Chaikittisilp, K.-S. Jang, S. A. Didas, J. R. Johnson, W. J. Koros, S. Nair, C. W. Jones, Aziridine-functionalized mesoporous silica membranes on polymeric hollow fibers: Synthesis and single-component CO_2 and N_2 Permeation properties. *Ind. Eng. Chem. Res.* **54**, 4407–4413 (2015).

56. C. Rosu, S. H. Pang, A. R. Sujan, M. A. Sakwa-Novak, E. W. Ping, C. W. Jones, Effect of extended aging and oxidation on linear poly(propylenimine)-mesoporous silica composites for CO_2 capture from simulated air and flue gas streams. *ACS Appl. Mater. Interfaces* **12**, 38085–38097 (2020).

57. A. H. Sharifabad, T. Behzad, M. Salami-Kalajahi, Glucose-responsive modified poly(propylene imine) dendrimer for self-regulated insulin delivery. *Macromol. Mater. Eng.* **311**, e00265 (2026).

58. R. V. Law, D. C. Sherrington, C. E. Snape, I. Ando, H. Kurosu, Solid-state ^{13}C MAS NMR studies of hyper-cross-linked polystyrene resins. *Macromolecules* **29**, 6284–6293 (1996).

59. G. Rim, P. Priyadarshini, M. Song, Y. Wang, A. Bai, M. J. Realff, R. P. Lively, C. W. Jones, Support pore structure and composition strongly influence the direct air capture of CO_2 on supported amines. *J. Am. Chem. Soc.* **145**, 7190–7204 (2023).

60. M. Song, G. Rim, G. Mirzazadeh, J. Hoffman, H. J. Moon, J. E. Leisen, O. G. Nik, R. P. Lively, C. W. Jones, Amine-dependent CO_2 sorption on amine-impregnated Mg_2(dobpdc) MOF under humid conditions. *Ind. Chem. Mater.* **4**, 52–64 (2026).

61. G. Rim, M. Song, L. Proaño, O. G. Nik, S. Parker, R. P. Lively, C. W. Jones, Humidity effects on sub-ambient direct air capture of CO_2 with amine functionalized Mg-Al LDHs and MMOs. *ACS EST Eng.* **5**, 204–214 (2025).

62. G. Rim, F. Kong, M. Song, C. Rosu, P. Priyadarshini, R. P. Lively, C. W. Jones, Sub-ambient temperature direct air capture of CO_2 using amine-impregnated MIL-101(Cr) enables ambient temperature CO_2 recovery. *JACS Au* **2**, 380–393 (2022).

63. M. Mourshed, Climatic parameters for building energy applications: A temporal-geospatial assessment of temperature indicators. *Renew. Energy* **94**, 55–71 (2016).

64. K. A. McColl, L. I. Tang, An analytic theory of near-surface relative humidity over land. *J. Clim.* **37**, 1213–1230 (2024).

65. A.-Y. Song, J. Young, J. Wang, S. N. Fricke, K. Piscina, R. Giovine, S. Garcia, M. van der Spek, J. A. Reimer, Discerning molecular-level CO_2 adsorption behavior in amine-modified sorbents within a controlled CO_2/H_2O environment towards direct air capture. *J. Mater. Chem. A* **12**, 25875–25886 (2024).

66. J. M. Kolle, M. Fayaz, A. Sayari, Understanding the effect of water on CO_2 adsorption. *Chem. Rev.* **121**, 7280–7345 (2021).

67. S. A. Didas, M. A. Salcwa-Novak, G. S. Foo, C. Sievers, C. W. Jones, Effect of amine surface coverage on the co-adsorption of CO_2 and water: Spectral deconvolution of adsorbed species. *J. Phys. Chem. Lett.* **5**, 4194–4200 (2014).

68. J. J. Lee, C.-H. Chen, D. Shimon, S. E. Hayes, C. Sievers, C. W. Jones, Effect of humidity on the CO_2 adsorption of tertiary amine grafted SBA-15. *J. Phys. Chem. C* **121**, 23480–23487 (2017).

69. R. J. Cvetanović, Y. Amenomiya, Application of a temperature-programmed desorption technique to catalyst studies, in *Advances in Catalysis*, D. D. Eley, H. Pines, P. B. Weisz, Eds. (Academic Press, New York, ed. 2, 1967), vol. 17, pp. 103–149.

70. R. A. Yang, D. R. Ganza, M. R. Smith, S. Cho, J. A. Vandermel, E. Jiang, M. L. Sarazen, Investigation of storage environments on aminopolymer stabilization within UiO-67(Zr) for CO_2 capture. *Mater. Adv.* **4**, 901–909 (2023).

71. S. Li, M. R. Cerón, H. V. Eshelman, A. J. Varni, A. Maiti, S. Akhade, S. H. Pang, Probing the kinetic origin of varying oxidative stability of ethyl- vs. propyl-spaced amines for direct air capture. *ChemSusChem* **16**, e202201908 (2023).

72. M. Song, S.-Y. Kim, G. Rim, R. P. Lively, C. W. Jones, Suppression of water adsorption in direct air capture using polyol/amine co-impregnated MIL-101(Cr) adsorbents. *Sep. Purif. Technol.* **381**, 135627 (2026).

73. J. S. A. Carneiro, G. Innocenti, H. J. Moon, Y. Guta, L. Proaño, C. Sievers, M. A. Sakwa-Novak, E. W. Ping, C. W. Jones, Insights into the oxidative degradation mechanism of solid

amine sorbents for CO_2 capture from air: Roles of atmospheric water. *Angew. Chem. Int. Ed.* **62**, e202302887 (2023).

74. Z. Zhou, T. Ma, H. Zhang, N. S. Sabeva, O. M. Yaghi, Fast and selective CO_2 capture from outdoor air by covalent organic frameworks. *Nat. Sustain.* **9**, 431–438 (2026).

75. A. Giri, J. Zhang, X. Deng, C. W. Jones, Humidity-enhanced direct air capture of carbon dioxide using amine-grafted covalent organic frameworks under ambient and sub-ambient temperatures. *Chem. Mater.* **38**, 2743–2754 (2026).

76. H. Li, Z. Zhou, T. Ma, K. Wang, H. Zhang, A. H. Alawadhi, O. M. Yaghi, Bonding of polyethylenimine in covalent organic frameworks for CO_2 capture from air. *J. Am. Chem. Soc.* **146**, 35486–35492 (2024).

77. H. Lyu, H. Li, N. Hanikel, K. Wang, O. M. Yaghi, Covalent organic frameworks for carbon dioxide capture from air. *J. Am. Chem. Soc.* **144**, 12989–12995 (2022).

78. J. Ren, Z. Zhang, N. Zhu, K. Yu, X. Shi, H. Li, Q. L. Yogesh, V. Joshi, S. Abubakar, D. Zhao, Bioinspired reconfiguration of alkylamines into metal-organic frameworks for robust direct air capture. *J. Am. Chem. Soc.* **147**, 43517–43528 (2025).

79. J. Espín, A. Justin, A. Hueber, A. Belin, S. Venkatachalam, H. D. Singh, E. Oveisi, W. L. Queen, A ship-in-a-bottle strategy: Crosslinking amines and epoxides inside MOF pores for enhanced CO_2 capture performance. *Adv. Mater.* **37**, e10138 (2025).

80. O. I.-F. Chen, C.-H. Liu, K. Wang, E. Borrego-Marin, H. Li, A. H. Alawadhi, J. A. R. Navarro, O. M. Yaghi, Water-enhanced direct air capture of carbon dioxide in metal–organic frameworks. *J. Am. Chem. Soc.* **146**, 2835–2844 (2024).

81. Y. Wang, G. Rim, M. Song, H. E. Holmes, C. W. Jones, R. P. Lively, Cold temperature direct air CO_2 capture with amine-loaded metal-organic framework monoliths. *ACS Appl. Mater. Interfaces* **16**, 1404–1415 (2024).

82. H. Dong, L.-H. Li, Z. Feng, Q.-N. Wang, P. Luan, J. Li, C. Li, Amine-functionalized quasi-MOF for direct air capture of CO_2. *ACS Mater. Lett.* **5**, 2656–2664 (2023).

83. L. Qiu, L. Peng, D. Moitra, H. Liu, Y. Fu, Z. Dong, W. Hu, M. Lei, D. Jiang, H. Lin, J. Hu, K. A. McGarry, I. Popovs, M. Li, A. S. Ivanov, Z. Yang, S. Dai, Harnessing the hybridization of a metal-organic framework and superbase-derived ionic liquid for high-performance direct air capture of CO_2. *Small* **19**, e2302708 (2023).

84. O. Shekhah, Y. Belmabkhout, Z. Chen, V. Guillerm, A. Cairns, K. Adil, M. Eddaoudi, Made-to-order metal-organic frameworks for trace carbon dioxide removal and air capture. *Nat. Commun.* **5**, 4228 (2014).

85. J. He, B. Wang, Y. Cao, H. Jin, Y. Wang, Q. Teng, Q. Meng, Grafted polystyrene resins with mixed-amine composition for enhanced CO_2 capture efficiency and stability. *Appl. Surf. Sci.* **732**, 166489 (2026).

86. Y. Wang, G. K. Li, The impact of co-adsorbed water on energy consumption and CO_2 productivity in direct air capture systems. *Sep. Purif. Technol.* **354**, 129415 (2025).

87. H. He, W. Li, M. Zhong, D. Konkolewicz, D. Wu, K. Yaccato, T. Rappold, G. Sugar, N. E. David, K. Matyjaszewski, Reversible CO_2 capture with porous polymers using the humidity swing. *Energy Environ. Sci.* **6**, 488–493 (2013).

88. K. A. S. Abhilash, T. Deepthi, R. A. Sadhana, K. G. Benny, Functionalized polysilsesquioxane-based hybrid silica solid amine sorbents for the regenerative removal of CO_2 from air. *ACS Appl. Mater. Interfaces* **7**, 17969–17976 (2015).

89. A. Wagner, B. Steen, G. Johansson, E. Zanghellini, P. Jacobsson, P. Johansson, Carbon dioxide capture from ambient air using amine-grafted mesoporous adsorbents. *Int. J. Spectrosc.* **2013**, 690186 (2013).

90. J. A. Wurzbacher, C. Gebald, A. Steinfeld, Separation of CO_2 from air by temperature-vacuum swing adsorption using diamine-functionalized silica gel. *Energy Environ. Sci.* **4**, 3584–3592 (2011).

91. Q. Grossmann, P. A. Saenz-Cavazos, N. Ferru, D. R. Williams, M. Mazzotti, Measuring and modeling water and carbon dioxide adsorption on amine functionalized alumina under direct air capture conditions. *Ind. Eng. Chem. Res.* **64**, 7165–7175 (2025).

92. P. Priyadarshini, G. Rim, C. Rosu, M. Song, C. W. Jones, Direct air capture of CO_2 using amine/alumina sorbents at cold temperature. *ACS Environ. Au* **3**, 295–307 (2023).
